# Low‐Temperature Isolation of a Labile Silylated Hydrazinium‐yl Radical Cation, [(Me_3_Si)_2_N−N(H)SiMe_3_]^.+^

**DOI:** 10.1002/chem.202200854

**Published:** 2022-04-29

**Authors:** Fabian Reiß, Alexander Villinger, Harald Brand, Wolfgang Baumann, Dirk Hollmann, Axel Schulz

**Affiliations:** ^1^ Institut für Chemie Universität Rostock Albert-Einstein-Straße 3a 18059 Rostock Germany; ^2^ Leibniz-Institut für Katalyse e.V. an der Universität Rostock Albert-Einstein-Straße 29a 18059 Rostock Germany

**Keywords:** hydrazine, nitrogen, radicals, silylium, synthesis

## Abstract

The oxidation of silylated hydrazine, (Me_3_Si)_2_N−N(H)SiMe_3_, with silver salts led to the formation of a highly labile hydrazinium‐yl radical cation, [(Me_3_Si)_2_N−N(H)SiMe_3_]^.+^, at very low temperatures (decomposition > −40 °C). EPR, NMR, DFT and Raman studies revealed the formation of a nitrogen‐centered radical cation along the N−N unit of the hydrazine. In the presence of the weakly coordinating anion [Al{OCH(CF_3_)_2_}_4_]^−^, crystallization and structural characterization in the solid state were achieved. The hydrazinium‐yl radical cation has a significantly shortened N−N bond and a nearly planar N_2_Si_3_ framework, in contrast to the starting material. According to DFT calculations, the shortened N−N bond has a total bond order of 1.5 with a π‐bond order of 0.5. The π bond can be regarded as a three‐π‐electron, two‐center bond.

## Graphical abstract

**Radical hydrazinium**: A highly labile hydrazinium‐yl radical cation, [(Me_3_Si)_2_N−N(H)SiMe_3_]˙^+^, formed by a one‐electron oxidation of tris(trimethylsilyl) hydrazine, (Me_3_Si)_2_N−N(H)SiMe_3_, with silver salts is presented. The structural and electronic parameters of the hydrazinium‐yl radical cation were elucidated, revealing that the spin density in the isolated radical is mainly localized along the N−N unit of the hydrazine moiety.

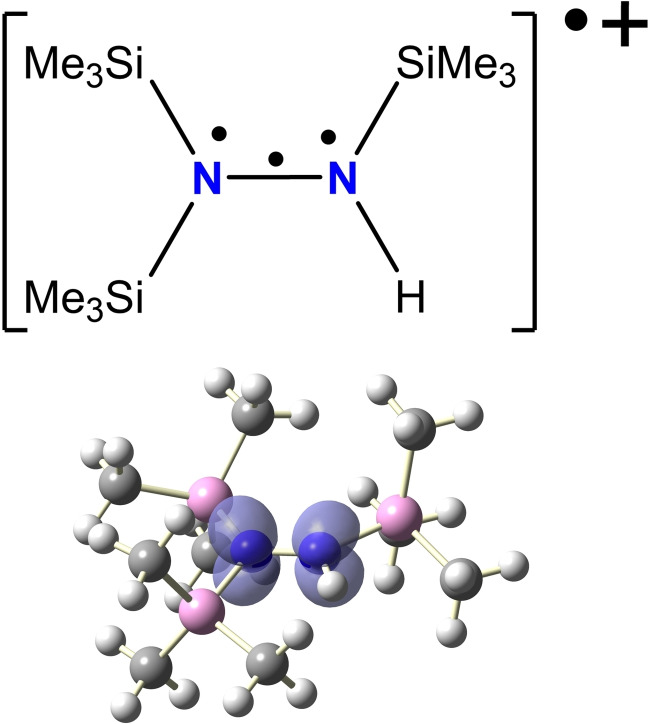

As early as 1875,[^1^, ^2^] Fischer first described aromatic‐substituted hydrazines and proposed the name *hydrazine* for this class of compounds, although the parent compound, N_2_H_4_, was not isolated until 1898 by Curtius (as a sulfate salt).^[3]^ Since its discovery, hydrazine has been a basic material for organic synthesis and is used primarily for the production of dyes, pharmaceuticals and crop protection agents.[^4^, ^5^] But hydrazine also occurs in the nitrogen cycle with its exchange between organic matter and the atmosphere and is essential for all life on Earth. Two of the most important natural processes, nitrogen fixation^[6]^ and the anammox process,[^7^, ^8^] carried out by specialized bacteria, pass through hydrazine, an extremely reactive substance. As such processes forming hydrazine in situ often involve one‐electron steps, corresponding radicals of hydrazine play a crucial role as intermediates, although very little is known experimentally so far.^[9]^ From a broader perspective, the chemistry of persistent and reactive radicals is diverse and is increasingly used in (in)organic synthesis,[^10^, ^11^] photo redox catalysis,^[12]^ molecular spintronics,^[13]^ optoelectronic and biological fields.^[14]^ In particular, there are a number of theory papers that deal with the bonding situation and thermodynamics of hydrazine‐based radicals.[^15^, ^16^, ^17^, ^18^, ^19^, ^20^, ^21^, ^22^, ^23^, ^24^] In most cases, the π‐radical type (unpaired spin distribution perpendicular to the plane of the N‐radical) proves to be the most stable.^[23]^ Starting from hydrazine, three radicals can be formally formed (Scheme 1): i) Formal oxidation gives the hydrazinium‐yl radical cation (**A**). ii) Bond breaking of the N−H or N−N bond gives rise to the hydrazinyl (**B**) or two aminyl radicals (**C**), respectively. Because the hydrogen‐substituted radicals **A**, **B** and **C** are extremely reactive, they cannot be easily synthesized and isolated. For example, Adams and Thomas showed that hydrazine can be chemically oxidized in aqueous sulfuric acid solution with ceric ammonium sulfate to the radical cation **A**, which was identified by in‐situ EPR spectroscopy.^[25]^ Furthermore, several studies show that both in solution and in solids, radical species **A** can be produced and spectroscopically studied by means of hard gamma irradiation.[^26^, ^27^, ^28^, ^29^, ^30^, ^31^, ^32^, ^33^, ^34^, ^35^, ^36^] In further in‐situ investigations, chemical oxidation as well as electrochemical oxidation were transferred to organically (Me, Ph) substituted hydrazine derivatives.[^28^, ^37^, ^38^] In a series of papers since the 1970s, Nelsen et al. reported sterically protected nitrogen‐based radical cations in which,[^39^, ^40^, ^41^, ^42^, ^43^, ^44^] for example, an [NN]^.+^ unit is incorporated into a bicyclic system, and later on the very stable [(*i*Pr)_4_N_2_]^.+^ with nitrate (*T*_m_=140 °C)^[44]^ and tosylate anions.

**Scheme 1:**
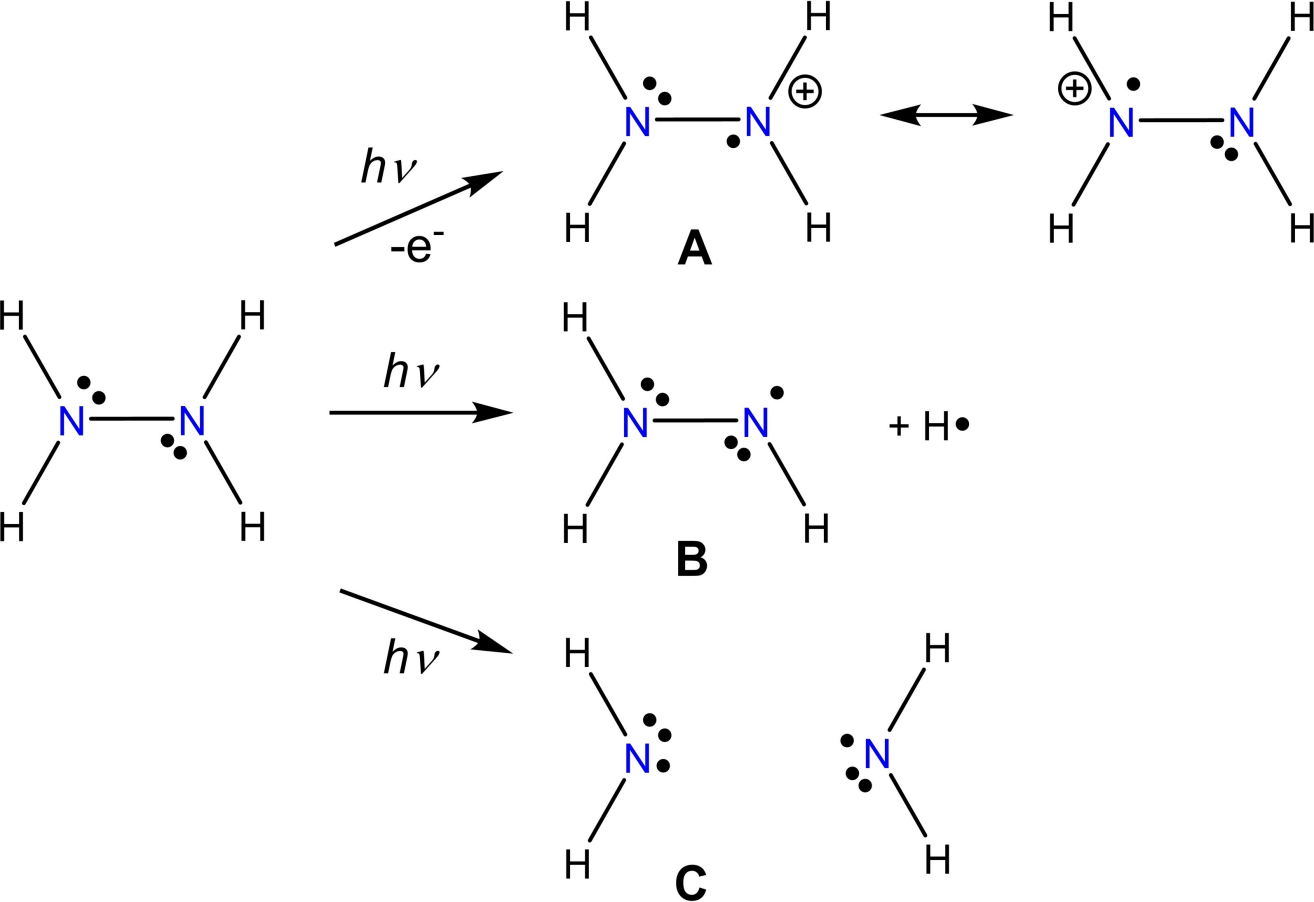
Hydrazine‐based radicals: **A** hydrazinium‐yl, **B** hydrazinyl, and **C** aminyl. Instead of N_2_H_4_, N_2_H_5_^+^ ions were often used as a source for the generation of type‐**A** radicals.

We were intrigued by the idea of preparing a silylated hydrazinium‐yl ion of the type [T_2_N−N(H)T]^.+^ (T=Me_3_Si) with a residual N−H bond, which might be the closest species to the parent [H_2_N−NH_2_]^.+^ radical cation, especially as we know that a Me_3_Si moiety can be understood as the “big brother” of the proton, as has been shown in a number of publications.[^45^, ^46^, ^47^, ^48^, ^49^, ^50^] For example, in analogy to the protonated species, such as [H−X−H]^+^ (X=halogen,[^51^, ^52^] pseudohalogen),[^53^, ^54^] [H_*n*+1_E]^+^] (E=group 15 element[^55^, ^56^] for *n*=3 and 16[^55^, ^57^, ^58^, ^59^] for *n*=2) or arenium ions^[60]^ in aromatic systems, also the silylated species[^48^, ^61^, ^62^, ^63^, ^64^, ^65^] can be isolated in the presence of a weakly coordinating anion.[^66^, ^67^] Like the protonated species, the silylated analogs can be used as T^+^‐transfer reagents or can migrate along bonds.^[68]^ The chemistry of silylated hydrazines has been extensively studied by Wiberg, Wannagat, West and others in the 1960s–1970s.[^69^, ^70^, ^71^, ^72^, ^73^, ^74^, ^75^, ^76^, ^77^, ^78^, ^79^] For example, an organosilylated hydrazinyl radical^[80]^ and phosphinyl‐substituted radical^[81]^ were even generated in situ and characterized by EPR spectroscopy (both are type **B** radicals, Scheme 1). Here we report the first isolation of a silylated hydrazinium‐yl, [T_2_N−N(H)T]^.+^, radical cation (type **A** radical in Scheme 1) stabilized by a weakly coordinating anion (wca).

For the synthesis of a silylated type **A** radical cation (Scheme 1), we first had to synthesize an appropriate silylated hydrazine, choosing T_2_N−N(H)T because it promised maximum steric protection while allowing reactivity at the one left N−H bond. Tri‐silylated hydrazine T_2_N−N(H)T (**1**) is best synthesized in a two‐step synthesis (Scheme 2): Starting from the hydrazinium hydrochloride, [N_2_H_5_]Cl, this is treated with Me_3_Si−Cl (T−Cl) in the presence of a strong base such as 1,2‐diaminoethane to give a mixture of the two di‐silylated species, 1,1‐T_2_N−NH_2_ and 1,2‐T(H)N−N(H)T, as well as tri‐silylated T_2_N−N(H)T. The mixture of the 1,1‐ and 1,2‐di‐silylated species can easily be separated from the tri‐silylated species by distillation. The separated mixture of 1,1‐ and 1,2‐T_2_N_2_H_2_ is then treated with *n*BuLi to give a mixture of lithium hydrazides, Li[T_2_N−NH] and Li[T(H)N−NT], which can also be isolated (see the Supporting Information). However, this mixture of lithium hydrazides can also be further converted in situ to T_2_N−N(H)T (**1**) by adding two equivalents of T−Cl. By this procedure T_2_N−N(H)T can be generated in over 70 % yield and obtained in high purity by repeated distillation (b.p.: 138 °C (100 mbar); 71 °C (6 mbar)).

**Scheme 2:**
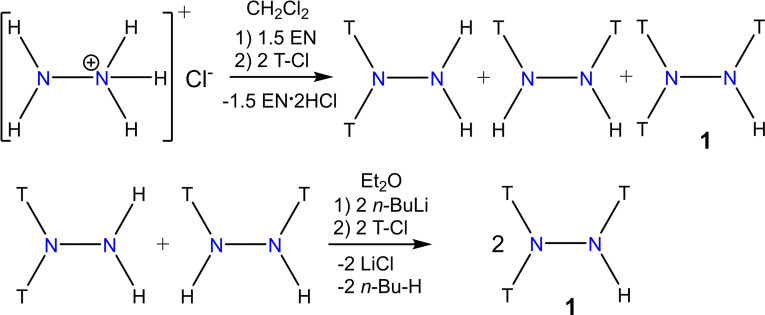
Synthesis of tri‐silylated hydrazine **1** (EN=1,2‐diaminoethane).

With tri‐silylated hydrazine **1** in hand, we treated it with different silver salts of the type Ag[wca] ([wca]^−^=[GaCl_4_]^−^, [SbF_6_]^−^ [F_3_CSO_3_]^−^, [BF_4_]^−^, [B(C_6_H_5_)_4_]^−^, [B(C_6_F_5_)_4_]^−^, [CHB_11_H_5_Br_6_]^−^, and [Al{OCH(CF_3_)_2_}_4_]^−^)^[82]^ in order to oxidize **1** to the radical cation [T_2_N−N(H)T]^.+^ (**1** ^.+^) as illustrated in Scheme 3. In the case of the anions [GaCl_4_]^−^ and [B(C_6_H_5_)_4_]^−^, no reaction at all was observed, whereas for [B(C_6_F_5_)_4_]^−^, [F_3_CSO_3_]^−^ and [CHB_11_H_5_Br_6_]^−^ mainly the corresponding hydrazinium salts, for example, [T_2_NN(T)H_2_][CHB_11_H_5_Br_6_] (**2**[CHB_11_H_5_Br_6_]) or [T_2_NN(H)T_2_][B(C_6_F_5_)_4_] (**3**[B(C_6_F_5_)_4_]) were observed or isolated (Schemes S3 and S4 in the Supporting Information). For both [wca]^−^=[SbF_6_]^−^ and [Al{OCH(CF_3_)_2_}_4_]^−^, only the desired product **1**⋅[wca] was observed. However, it should be mentioned that only the combination [T_2_N−N(H)T]^.+^ with the [Al{OCH(CF_3_)_2_}_4_]^−^ anion is suitable for isolation (see the Supporting Information), of which yellow crystals could be obtained from CH_2_Cl_2_ at −80 °C. The problem stems from the fact that the yellow salts with the radical cation **1** ^.+^ decompose already above −20 °C in the solid as demonstrates by LT‐Raman studies (Figure S13), in solution the decomposition starts even earlier, so that these salts have to be kept at temperatures < −40 °C. Decomposition probably begins with the deprotonation of **1** ^.+^, producing the hydrazinyl radical T_2_N−NT⋅ (radical of type **B** in Scheme 1), which decomposes further. In addition to deprotonation, **1** ^.+^ can also act as a T^+^ transfer reagent, which then leads to an in‐situ‐generated T_2_N−NH⋅ radical. Therefore, it is not surprising that we were able to isolate simple silylated hydrazinium salts, with the cation [T_2_NN(T)H_2_]^+^ (**2** ^+^) or [T_2_NN(H)T_2_]^+^ (**3** ^+^) very frequently in many reactions (see above). The lability of **1** ^.+^ contrasts to Nelsen's fully protected compounds, which are stable at room temperature (see above).

**Scheme 3:**
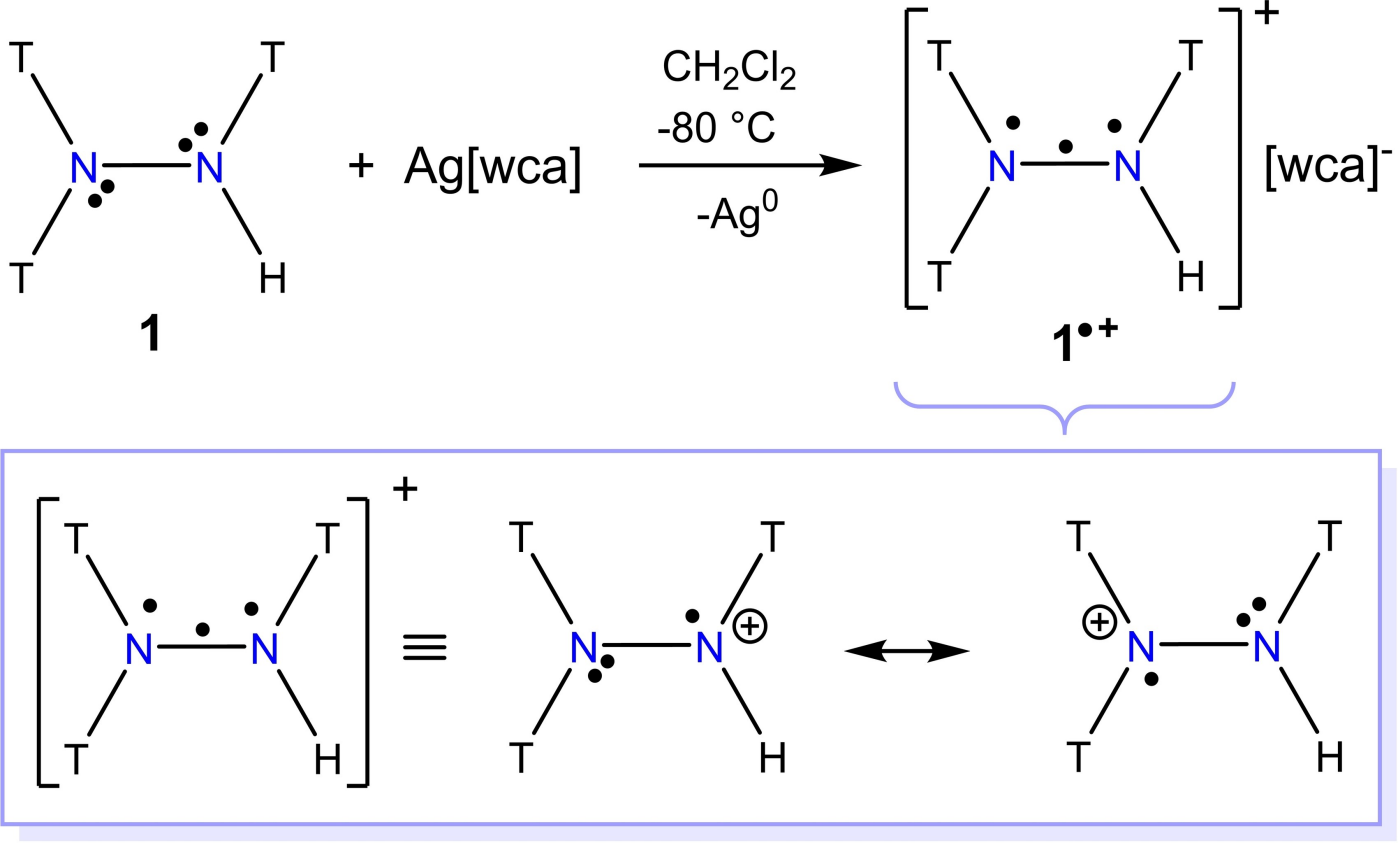
Synthesis of salts containing the radical cation **1** ^.+^ (observed for [wca]^−^=[SbF_6_]^−^ and [Al{OCH(CF_3_)_2_}_4_]^−^). Below: resonance scheme of the three‐π‐electron, two‐center bond.

The existence of the radical cation **1** ^.+^ in solution as well as solid state was unequivocally proven by X‐ray, EPR and Raman studies. For EPR analysis (Figure 1), isolated crystals were dissolved at a temperature of −40 °C and then cooled down to −73 °C. The EPR spectra of **1** ^.+^ in CH_2_Cl_2_ solution confirm their radical nature. The hyperfine structure is consistent with a hyperfine coupling to the two N atoms and the one H atom. The coupling constant of *A*_H_ = 11.7 G and *A*_N_ = 9.1 G are in the same range as for the transient [N_2_H_4_]^.+^ radical cation (*A*_H_=11.0 G and *A*_N_ = 11.5 G).[^25^, ^30^] Also in the low‐temperature ^1^H solution NMR experiment at −50 °C in CD_2_Cl_2_, the radical is also recognized because, signals due to **1** are absent (**1**: *δ*[^1^H]=2.11 N−H, 0.08 C−H) and a very broad peak, shifted to higher frequency (**1** ^.+^: *δ*[^1^H]=11.8 with *ν*_1/2_≈5300 Hz) shows up. No discrimination of individual atom sites is possible. In line with this observation, only signals of the anion show up in the ^13^C NMR spectrum.

**Figure 1:**
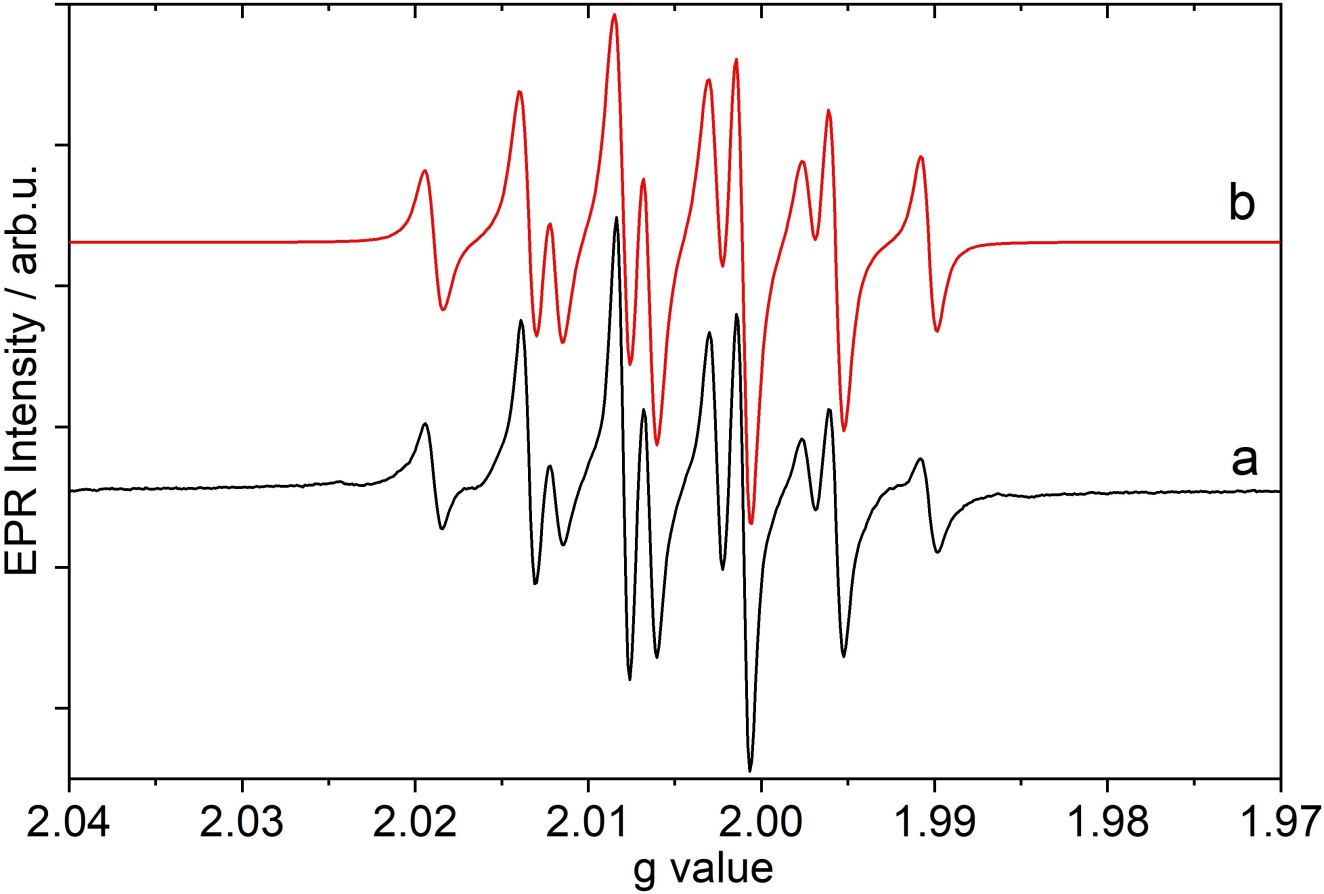
EPR spectrum of **1** ^.+^ at 200 K in CH_2_Cl_2_, a) measured and b) simulated by using the parameters *g*=2.0045, *A*_H_=11.7 G, 2 x *A*_N_=9.1 G, line width Δ*B*=1.5 G. Simulation by EPRSim32.[^83^, ^84^]

The Raman spectrum of **1** ^.+^ also shows large differences compared to the neutral starting material **1**. For example, the N−H stretching vibration shifts to a smaller wavenumber (**1**: 3348 vs. **1** ^.+^: 3303 cm^−1^), while the N−N vibration is shifted by about 250 cm^−1^ to a higher wavenumber (**1**: 1072 vs. **1** ^.+^: 1321 cm^−1^). The latter implies a significant enhancement of the N−N bond strength and indicates a significant N−N double bond character in **1** ^.+^ (cf. 1555 in diazene T−N=N−T, 1570 in [T_2_N=N−T]^+^, 1062 cm^−1^ in Hg[T−N−NT_2_]_2_).^[49]^

To experimentally investigate the structural changes upon oxidation of **1** to the radical cation **1** ^.+^, some structures along the reaction pathway were determined by single‐crystal X‐ray structural analysis (Figure 2), including the decomposition products [T_2_N−N(T)H_2_][CHB_11_H_5_Br_6_] (**2**[CHB_11_H_5_Br_6_]) or [T_2_N−N(H)T_2_][B(C_6_F_5_)_4_] (**3**[B(C_6_F_5_)_4_]) and the lithium hydrazide salt [Li(TN−NT_2_)]_2_ ([Li**4**]_2_, see the Supporting Information). Selected structural parameter are summarized in Table 1. The radical cation salt **1**⋅[Al{OCH(CF_3_)_2_}_4_] crystallizes like the neutral parent compound **1** in the monoclinic space group *P*2_1_/*c* with Z=4, but the volume of the unit cell has more than doubled (1646 vs. 3917 Å^3^), which is due to the larger space requirement of the anion in **1**⋅[Al{OCH(CF_3_)_2_}_4_]. There are only very weak interactions between the radical cation and the alkoxy‐aluminate counterion (cf. smallest F_anion_⋅⋅⋅H1_cation_=2.68 Å), but these do not affect significantly the molecular structure of the radical cation. Probably the most striking structural motif is the N_2_Si_3_ skeleton, which is almost planar (as in the [T_2_N=NT]^+^ cation **5** ^+^ with Si−N−N−Si dihedral angles close to 180 or 0°), while in the neutral hydrazine **1** both NR_2_ planes are almost orthogonal to each other (∢(Si1−N1−N2−Si3)=−179.0 in **1** ^.+^ vs. 96.9° for **1**, Figure 2, Table 1). The latter is also true for the anion **4** ^−^, all of which have one free electron pair per N atom. The Pauli repulsion between these two lone pairs leads to this energetically more favorable orthogonal arrangement of the NR_2_ planes with respect to each other. Upon the oxidation of **1**, an electron is removed from one electron pair and a partial N−N π bond is formed, which in turn leads to a favorable planar N_2_Si_3_ framework. This can also be seen from the significantly shorter N−N distance of 1.343(2) Å in **1** ^.+^ that is about 0.115 Å shorter than in **1** (1.458(3) Å, cf. Σ*r*_cov_(N−N)=1.42 and Σ*r*_cov_(N=N)=1.20 Å)^[85]^ and is well in line with the N−N distance of 1.333(4) Å in [(*i*Pr)_4_N_2_]^.+^.^[44]^ This mediate value indicates a partial double bond character which is in **1** ^.+^ somewhat smaller as in **5** ^+^ (1.254(2) Å).^[49]^ Finally, it should be noted that all tri‐coordinated silylated nitrogen atoms in all considered species are almost trigonal planar due to hyperconjugative effects between the lone pairs located at the N atoms (in p‐type atomic orbitals) and antibonding σ*(Si−C) bond orbitals (see the Supporting Information). In contrast to **1** ^.+^, [(*i*Pr)_4_N_2_]^.+[44]^ as well as [H_2_N−NH_2_]^.+^ feature a larger deviation from planarity due to the absence of significant hyperconjugation in both species in accordance with computations that predict a *C*_2h_ symmetric gas‐phase structure of the parent system [H_2_N−NH_2_]^.+^.^[86]^

**Figure 2:**
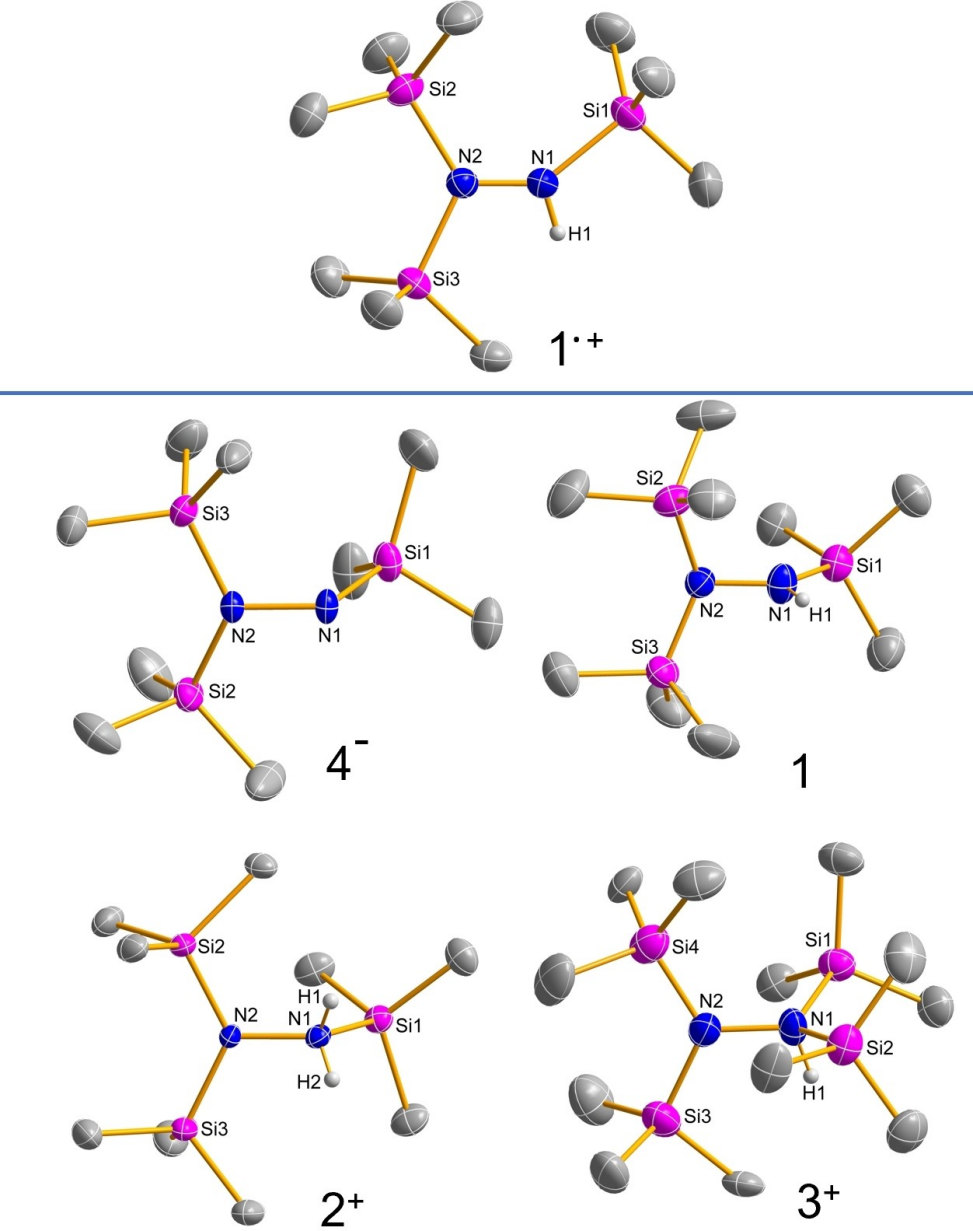
Top: Molecular structure of the radical cation **1** ^.+^ in the crystal. Ellipsoids are drawn at 50 % probability at 123(2) K; bottom: the molecular structures of **1**, **2** ^+^, **3** ^+^ and **4^−^** are shown for comparison. All counter ions and methyl protons are omitted for clarity. Selected structural data are listed in Table 1.

**Table 1:** Selected experimental structural parameters (Figure 2) of **1**, **1** ^.+^, **2** ^+^, **3** ^+^, **4^−^** and for comparison [T_2_N=NT]^+^ (**5** ^+^). In addition, calculated values are given in italics (M06/def2tzvpp).

	Bond length [Å]		Bond angle [°]	
	N1−N2	N1−Si1	Si1N1N2Si3	Si3N2Si2
T_2_NN(H)T^[a]^ (**1**)	1.458(3) *1.437*	1.718(6) *1.737*	96.9(5) *89.59*	135.7(3) *133.8*
[T_2_NN(H)T]^.+^ (**1** ^.+^)	1.343(2) *1.332*	1.822(2) *1.839*	−179.0(1) *−173.09*	122.74(8) *121.7*
[T_2_NN(T)H_2_]^+^ (**2** ^+^)	1.467(3) *1.444*	1.891(2) *1.915*	85.6(2) *85.15*	127.8(1) *126.9*
[T_2_NN(H)T_2_]^+^ (**3** ^+^)	1.499(5) *1.465*	1.875(5) *1.892*	104.8(3) *107.21*	123.6(2)^[b]^ *122.3*
[T_2_NNT]^−^ (**4** ^−^)	1.517(2) *1.480*	1.697(2) *1.706*	−68.1(2) *−70.13*	126.2(1) *124.0*
[T_2_NNT]^+^ (**5** ^+^)^[c]^	1.254(2)	1.829(1)	−175.3(1)	124.14(6)

[a] Only values of the main part (A) are listed. [b] Si3N2Si4 is listed. [c] Data were taken from ref. [49].

To better understand the structure, bonding situation and charge distribution, DFT calculation was performed using the M06 functional in combination with the def2tzvpp basis set. As shown in Table S13, the quantum mechanical calculations also reveal a nearly planar N_2_Si_3_H framework for **1** ^.+^ as well as a significantly shortened N−N bond, in agreement with the experimental SC‐XRD data. The agreement for all other species **1**, **2** ^+^, **3** ^+^ and **4** ^−^ is also very good (Table 1). The calculated Mulliken spin density is mainly localized at both N atoms, with a slightly smaller value for N1 (0.43) than for N2 (0.45).^[87]^ The spin densities at the three Si atoms are much smaller, about 0.02, while they are negligible for the C and H atoms. That is, the radical cation **1** ^.+^ can be described in good approximation as a nitrogen‐centered radical. The N−N bonds are nearly covalent according to the NBO analysis (NBO=natural bond analysis), with a N−N σ bond and a one‐electron π bond localized besides a one electron lone pair at each N atom; this agrees with the EPR measurements, showing a distinct coupling to both nitrogen atoms. This description corresponds to a three‐π‐electron, two‐center bond in the MO picture, where the bonding π‐MO is doubly and the antibonding π* MO is singly occupied (Figure 3).^[88]^ This gives a formal π bond order of 0.5 (calcd: 0.46), which is also consistent with the calculated total NLMO bond order for the N−N bond of 1.44 (cf. 0.99 for **1**, see the Supporting Information, NLMO=natural localized molecular orbital), in accord with the rather short N−N bond. This also makes it clear that the spin density has local π symmetry along the N−N axis (Figure 3).

**Figure 3:**
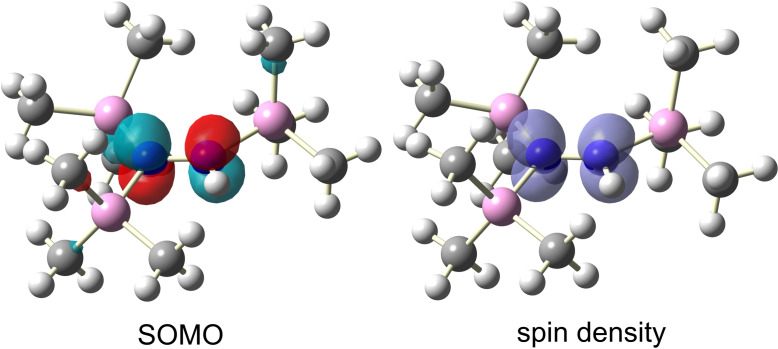
Left: Plot of the SOMO of **1** ^.+^ (isosurfaces set at 0.068 a.u). Right: Plot of the spin density of **1** ^.+^ (isosurfaces set at 0.006 a.u.). Optimization of structures/calculations at the M06/def2tzvpp level of theory in the gas phase.

In summary, upon oxidation of tris(trimethylsilyl)hydrazine **1** with silver salts, the highly labile trisilylated radical cation [T_2_N−N(H)T]^.+^ (**1** ^.+^ ) was observed in solution. The high lability of **1** ^.+^ is largely determined by the remaining N−H bond. The radical character was studied by EPR experiments, which clearly indicated the presence of a nitrogen–nitrogen‐centered radical cation. Calculations confirmed that the spin density is mainly localized at both nitrogen atoms. In the presence of the alkoxy‐aluminate anion, **1**⋅[Al{OCH(CF_3_)_2_}_4_] could be crystallized and structurally characterized. [T_2_N−N(H)T]^.+^ represents a silylated hydrazinium‐yl ion that could be structurally characterized, thus closing a gap in hydrazine/nitrogen chemistry.

## Experimental Section

A full set of analytical and theoretical data along with the detailed description of the synthesis are given in the Supporting Information.

**X‐ray crystallography**: Deposition Numbers 2154462 (for **1**), 2154465 (for **1**[Al{OCH(CF_3_)_2_}_4_]), 2154463 (for **2**[CHB_11_H_5_Br_6_]), 2154466 (for **3**[B(C_6_F_5_)_4_]), 2154464 (for [Li**4**]_2_) contain the supplementary crystallographic data for this paper. These data are provided free of charge by the joint Cambridge Crystallographic Data Centre and Fachinformationszentrum Karlsruhe Access Structures service.

## Conflict of interest

The authors declare no conflict of interest.

## Supporting information

As a service to our authors and readers, this journal provides supporting information supplied by the authors. Such materials are peer reviewed and may be re‐organized for online delivery, but are not copy‐edited or typeset. Technical support issues arising from supporting information (other than missing files) should be addressed to the authors.

Supporting Information

## Data Availability

The data that support the findings of this study are available from the corresponding author upon reasonable request.
