# Supplementary material for: Low‐Temperature Isolation of a Labile Silylated Hydrazinium‐yl Radical Cation, [(Me3Si)2N−N(H)SiMe3].+
Source: Chemistry. 2022 Apr 29;28(33):e202200854. doi: 10.1002/chem.202200854 (PMC9321631; doi:10.1002/chem.202200854)
Supplement: Supplementary file 1 — Supporting Information [file CHEM-28-0-s001.pdf]

Chemistry–A European Journal

Supporting Information

Low-Temperature Isolation of a Labile Silylated Hydrazinium-yl Radical Cation, $[(Me_3Si)_2N{-}N(H)SiMe_3]^{\bullet +}$

Fabian Reiß, Alexander Villinger, Harald Brand, Wolfgang Baumann, Dirk Hollmann, and Axel Schulz*

This file includes:

1 General Experimental Information 2

2 Structure elucidation 4

3 Comparison of selected structural features 13

4 Synthesis of Starting Materials 14

5 Synthesis of compounds 16

6 NMR Spectra 18

7 IR, Raman and Vis Spectra 23

8 EPR – Spectrum of **1·[Al{OCH(CF_3)$_2$}$_4$]** 30

9 Computational Details 31

10 References 86

1 General Experimental Information

All manipulations were carried out under oxygen- and moisture-free conditions under argon using standard Schlenk or drybox techniques. Dichloromethane was purified according to a literature procedure,[1] dried over P_4O_{10}, followed by CaH_2, freshly distilled and degassed prior to use. Diethyl ether, *n*-hexane and *n*-pentane were dried over Na/benzophenone and freshly distilled prior to use. Hydrazinium chloride H_5N_2Cl (Sigma-Aldrich, 97%) was recrystallized from dry methanol prior to use. Ethylenediamine was dried over NaOH followed by CaH_2 and freshly distilled prior to use. Trimethylsilyl chloride (Merck, 98%) was dried over CaH_2 and freshly distilled prior to use. Silver hexafluoroantimonate $AgSbF_6$ (abcr, 98%), silver *closo*-7,8,9,10,11,12-hexabromocarboranate $Ag[CHB_{11}H_5Br_6]$,[2] silver triflate $Ag[CF_3SO_3]$,[3] silver tetrakis(1,1,1,3,3,3-hexafluoro-2-isopropoxy)aluminate $Ag[Al(OCH(CF_3)_2)_4]$,[4] silver tetrakis(pentafluorophenyl)borate toluene trisolvate $Ag(toluene)_3[B(C_6F_5)_4]$,[5] and silver tetrachloridogallate $Ag[GaCl_4]$[6] were prepared according to literature procedures.

NMR: ^{29}Si-INEPT, ^{1}H,^{29}Si HMQC, ^{13}C{^{1}H}, ^{11}B{^{1}H}, ^{1}H,^{15}N HMBC and ^{1}H NMR spectra were obtained on a Bruker ARX 300 or AVANCE 400 spectrometer and were referenced internally to the deuterated solvent (^{13}C, CD_2Cl_2, $\delta_{reference}$ = 53.8 ppm, C_7D_8, $\delta_{reference}$ = 20.4 ppm) or to protic impurities in the deuterated solvent (^{1}H, $CDHCl_2$, $\delta_{reference}$ = 5.32 ppm, ^{1}H, $CHCl_3$, $\delta_{reference}$ = 7.26 ppm, C_7D_7H, $\delta_{reference}$ = 2.03 ppm). The ^{1}H-^{15}N-HMBC spectra were obtained on a Bruker AVANCE 400 spectrometer, chemical shifts of nitrogen-15 are given relative to a reference frequency calculated from the actual ^{1}H reference frequency and $\Xi(^{15}N)$ = 10.136 767 MHz (which corresponds to neat nitromethane at 0 ppm).

IR: Nicolet 6700 FT-IR spectrometer with a Smart Endurance ATR device or Nicolet 380 FT-IR (HeNe laser, 633nm) with Smart Orbit ATR device.

Raman: Horiba LabRAM HR Raman microscope with Olympus optics (10x NA 0.25), 784 nm Laser diode (100 mW, air-cooled) or 473 nm Ar^+ Laser (20 mW, air-cooled) and 800 mm focal length spectrograph. Linkam THMS600 System and PE95/T95 System Controller was used for low temperature Raman measurements. Samples were prepared under N_2 atmosphere in a low temperature mounting device, cooled with an ENRAF NONIUS FR558-S.

MS: Finnigan MAT 95-XP from Thermo Electron was used (CI^+, *isobutene*; EI^+, T = 200 °C, 70.0 V).

CHN analyses: Analysator Flash EA 1112 from Thermo Quest.

Decomposition points: Decomposition point for solid **1·[Al{OCH(CF$_3$)$_2$}$_4$]** was estimated with the Raman microscope during slow warming the Linkam THMS600 System. Approximately Heating-rate 10 °C/min. The Decomposition point of **1·[Al{OCH(CF$_3$)$_2$}$_4$]** in CH_2Cl_2 solution was estimated by low Temperature UV/Vis measurements.

UV/Vis: Lambda 19 Perkin Elmer, Software Lambda-SPX 1. Samples were measured in CH_2Cl_2 at different temperatures using an O.K. Tec 101-231 optrode. Settings: Scan: 200-800 nm, Interval 0.5 nm, Scan speed 120 nm/min, Smooth 1, Slit 1 nm

DSC: DSC 823e from Mettler-Toledo (Heating-rate 5 °C/min) was used, uncorrected melting points were reported.

EPR: Bruker EMXCW-microspectrometer equipped with ER 4119HS-WI high-sensitivity optical resonator.

2 Structure elucidation

X-ray Structure Determination: X-ray quality crystals of all compounds were selected in Galden-HT230 oil (Solvay Solexis) at 173 K and prepared under N_2 atmosphere in a low temperature mounting device, cooled with an ENRAF NONIUS FR558-S. All samples were cooled to 173 (2) K during measurement. The data were collected on a Bruker Apex Kappa II CCD diffractometer using graphite monochromated Mo Kα radiation (λ = 0.71073). The structures were solved by direct methods (*SHELXS-97*)[7] and refined by full-matrix least squares procedures (*SHELXL-97*).[8] Semi-empirical absorption corrections were applied (SADABS).[9] All non hydrogen atoms were refined anisotropically, hydrogen atoms were included in the refinement at calculated positions using a riding model. Refinement details can be found in the cif files.

Table S 1: Crystallographic details of **1**, **1[[Al{OCH(CF_3)$_2$}$_4$** and **2**

	**1**	**1·[Al{OCH(CF_3)$_2$}$_4$]**	**2[CHB$_{11}$H$_5$Br$_6$]**
Chem. Formula	$C_9H_{28}N_2Si_3$	$C_{21}H_{32}AlF_{24}N_2O_4Si_3$	$C_{10}H_{35}B_{11}Br_6N_2Si_3$
Form. Wght. [g mol^{-1}]	248.60	943.73	866.04
Colour	Colourless	Yellow	Colourless
Cryst. system	Monoclinic	Monoclinic	Monoclinic
Space group	P21/c	P21/c	P21/c
a [Å]	14.8783(5)	10.5644(4)	13.9342(7)
b [Å]	8.9868(3)	12.1707(5)	11.8110(6)
c [Å]	12.3301(4)	30.7482(11)	19.1766(9)
α [°]	-	-	-
β [°]	92.915(2)	97.793(1)	93.443(3)
γ [°]	-	-	-
V [Å^3]	1646.52(9)	3917.0(3)	3150.3(3)
Z	4	4	4
$\rho_{calc.}$ [g cm^{-3}]	1.003	1.600	1.826
μ [mm^{-1}]	0.265	0.289	7.768
$\lambda_{MoK\alpha}$ [Å]	0.71073	0.71073	0.71073
T [K]	173(2)	173(2)	173(2)
Measured reflections	22726	30226	37619
Independent	3965	8521	9200
Reflections with I >	2993	6591	6143
$R_{int.}$	0.0488	0.0295	0.0580
F(000)	552	1900	1664
R_1 (R [F^2 > 2σ(F^2)])	0.0362	0.0382	0.0351
wR_2 (F^2)	0.1036	0.1013	0.0721
GooF	1.037	1.012	1.014
Parameters	191	755	309
CCDC #	2154462	2154465	2154463

Table S 2: Crystallographic details of **3** and **4_2**.

	**3[B(C_6F_5)$_4$]**	**[Li4]$_2$**
Chem. Formula	$C_{36}H_{37}BF_{20}N_2Si_4$	$C_{18}H_{54}Li_2N_2Si_3$
Form. Wght. [g mol^{-1}]	1000.84	509.07
Colour	Colourless	Colourless
Cryst. system	Monoclinic	Monoclinic
Space group	P21/c	P21/c
a [Å]	12.2983(6)	13.265(3)
b [Å]	28.5494(13)	9.923(2)
c [Å]	12.4023(5)	14.669(6)
α [°]	-	-
β [°]	101.935(3)	123.56(2)
γ [°]	-	-
V [Å^3]	4260.4(3)	1609.0(9)
Z	4	2
$\rho_{calc.}$ [g cm^{-3}]	1.560	1.051
μ [mm^{-1}]	0.258	0.271
$\lambda_{MoK\alpha}$ [Å]	0.71073	0.71073
T [K]	173(2)	173(2)
Measured reflections	38324	15153
Independent	7499	3318
Reflections with *I* >	4428	2297
$R_{int.}$	0.0784	0.0598
F(000)	2032	560
R_1 (R [F^2 > 2σ(F^2)])	0.0548	0.0409
wR_2 (F^2)	0.1419	0.1033
GooF	1.013	1.013
Parameters	636	145
CCDC #	2154466	2154464

Scheme S 1: Numbering scheme of **1**.

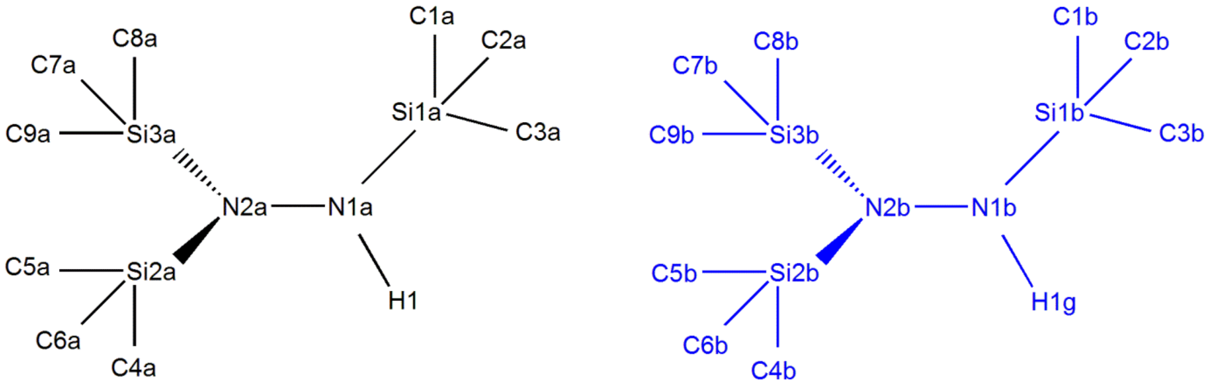

Table S 3: Selected bond lenghts (Å), angles and torsion angles (°) of (**1**).

**N1A—N2A**	1.458 (3)	**Si2A—C4A**	1.862 (3)
**N1B—N2B**	1.454 (8)	**Si3A—C7A**	1.856 (2)
**N1A—Si1A**	1.719 (2)	**Si3A—C8A**	1.857 (3)
**N1B—Si1B**	1.721 (8)	**Si3A—C9A**	1.864 (3)
**N1A—H1**	0.83 (2)	**N2A—N1A—Si1A**	128.1 (3)
**N2A—Si2A**	1.733 (2)	**N2A—N1A—H1**	112.3 (14)
**N2B—Si2B**	1.728 (7)	**Si1A—N1A—H1**	119.3 (14)
**N2A—Si3A**	1.733 (2)	**N1A—N2A—Si3A**	111.3 (2)
**N2B—Si3B**	1.732 (7)	**N1A—N2A—Si2A**	108.7 (2)
**Si1A—C1A**	1.862 (2)	**Si3A—N2A—Si2A**	135.7 (2)
**Si1A—C3A**	1.861 (2)	**Si1A—N1A—N2A—Si3A**	96.9 (5)
**Si1A—C2A**	1.865 (3)	**Si1A—N1A—N2A—Si2A**	−102.4 (5)
**Si2A—C5A**	1.859 (3)	**N2A—N1A—Si1A—C1A**	8.4 (5)
**Si2A—C6A**	1.861 (3)		
		**∑ (∠ N1A)**	359.7
		**∑ (∠ N2A)**	355.7

Scheme S 2: Numbering scheme of **1·[Al{OCH(CF_3)$_2$}$_4$]**.

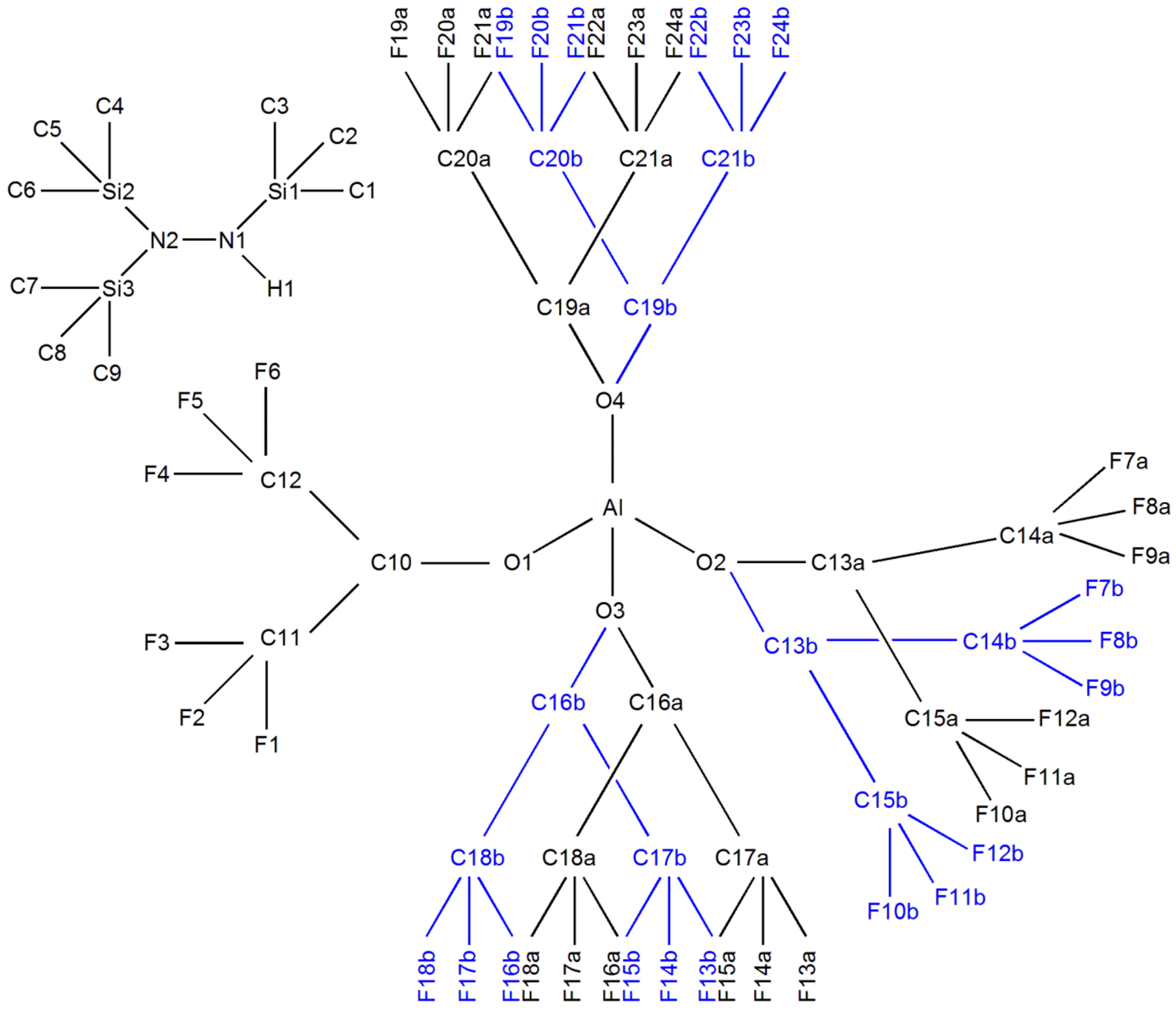

Table S 4: Selected bond lenghts (Å), angles and torsion angles (°) of **1·[Al{OCH(CF_3)$_2$}$_4$]**.

**N1—N2**	1.343 (2)	**O4—C19B**	1.32 (3)
**N1—Si1**	1.8224 (16)	**O4—C19A**	1.378 (3)
**N1—H1**	0.87 (2)	**N2—N1—Si1**	140.43 (13)
**N2—Si2**	1.8209 (15)	**N2—N1—H1**	107.4 (14)
**N2—Si3**	1.8397 (14)	**Si1—N1—H1**	112.2 (14)
**Al—O4**	1.7296 (13)	**N1—N2—Si2**	122.04 (11)
**Al—O2**	1.7326 (14)	**N1—N2—Si3**	115.02 (11)
**Al—O3**	1.7335 (13)	**Si2—N2—Si3**	122.74 (8)
**Al—O1**	1.7385 (12)	**N1—Si1—C1**	98.29 (9)

**O1—C10**	1.374 (2)	**Si1—N1—N2—Si2**	5.9 (3)
**O2—C13B**	1.31 (2)	**Si1—N1—N2—Si3**	−179.05 (14)
**O2—C13A**	1.371 (3)	**N2—N1—Si1—C1**	−171.6 (2)
**O3—C16B**	1.39 (3)	**N1—N2—Si3—C9**	−8.94 (15)
**O3—C16A**	1.371 (3)		
		**∑ (∠ N1)**	360.03
		**∑ (∠ N2)**	359.8

Scheme S 3: Numbering scheme of **2**[$CHB_{11}H_5Br_6$].

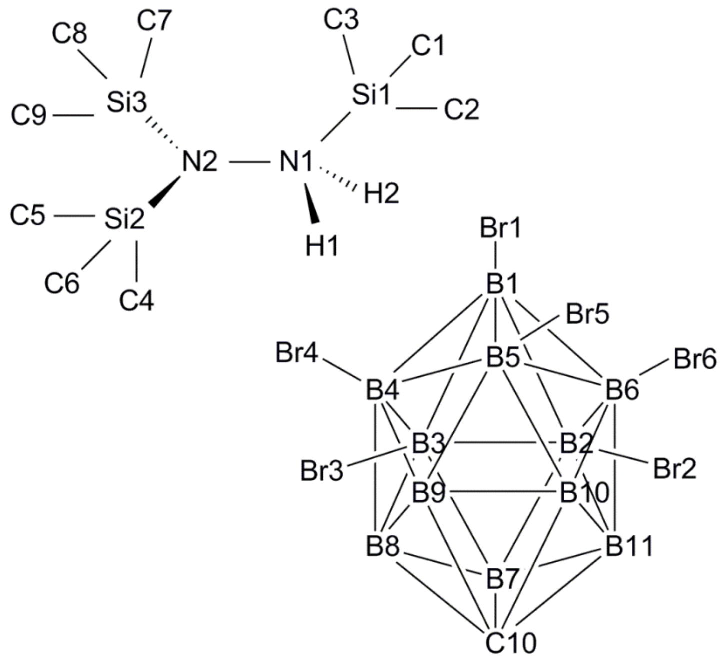

Table S 5: Selected bond lenghts (Å), angles and torsion angles (°) of **2**[$CHB_{11}H_5Br_6$].

**N1—N2**	1.467 (3)	**N2—N1—H2**	108 (2)
**N1—Si1**	1.891 (2)	**Si1—N1—H1**	109.8 (19)
**N1—H1**	0.86 (3)	**Si1—N1—H2**	107 (2)
**N1—H2**	0.81 (3)	**H1—N1—H2**	106 (3)
**N2—Si2**	1.764 (2)	**N1—N2—Si2**	116.17 (17)
**N2—Si3**	1.776 (2)	**N1—N2—Si3**	116.03 (17)
**Br1—B1**	1.954 (3)	**Si2—N2—Si3**	127.78(12)
**Br2—B2**	1.949 (3)	**Si1—N1—N2—Si2**	-96.1 (2)
**Br3—B3**	1.950 (3)	**Si1—N1—N2—Si3**	85.6(2)
**Br4—B4**	1.946 (3)	**N2—N1—Si1—C3**	20.8(3)
**Br5—B5**	1.962 (3)	**N1—N2—Si3—C9**	148.1(2)
**N2—N1—Si1**	119.42 (18)		
**N2—N1—H1**	106.1 (19)	**∑ (∠ N2)**	359.98

Scheme S 4: Numbering scheme of **3**[B(C_6F_5)$_4$].

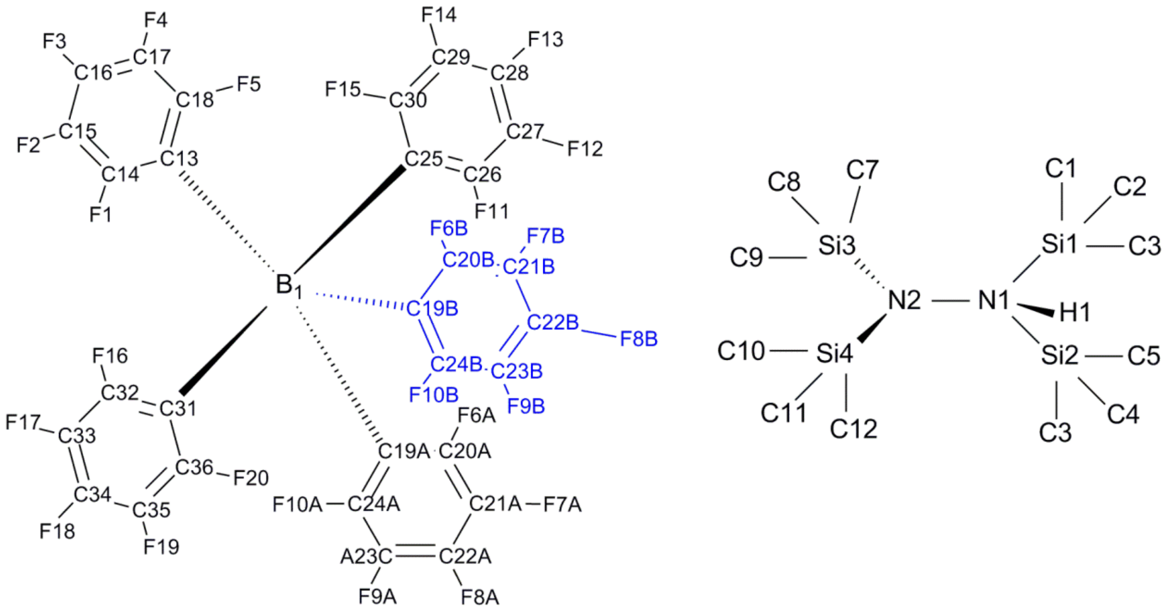

Table S 6: Selected bond lenghts (Å), angles and torsion angles (°) of **3**[B(C_6F_5)$_4$].

**N1—N2**	1.499 (5)	**N2—N1—H1**	107(3)
**N1—Si1**	1.875 (3)	**Si1—N1—H1**	88(3)
**N1—Si2**	1.879 (4)	**Si1—N1—Si2**	122.02(19)
**N1—H1**	1.03 (5)	**Si2—N1—H1**	100 (3)
**N2—Si3**	1.791 (4)	**N1—N2—Si3**	115.0 (3)
**N2—Si4**	1.776 (4)	**N1—N2—Si4**	120.7(3)
**B1—C19A**	1.643 (8)	**Si3—N2—Si4**	123.6(2)
**B1—C31**	1.654 (6)	**Si1—N1—N2—Si3**	104.8 (3)
**B1—C25**	1.655 (6)	**Si1—N1—N2—Si4**	-66.0(4)
**B1—C13**	1.660 (6)	**N2—N1—Si1—C3**	-156.7(3)
**B1—C19B**	1.681 (15)	**N1—N2—Si3—C9**	-122.3(3)
**N2—N1—Si1**	116.5 (2)		
**N2—N1—Si2**	115.4(2)	**∑ (∠ N2)**	359.3

Scheme S 5: Numbering scheme of **[Li4]₂**.

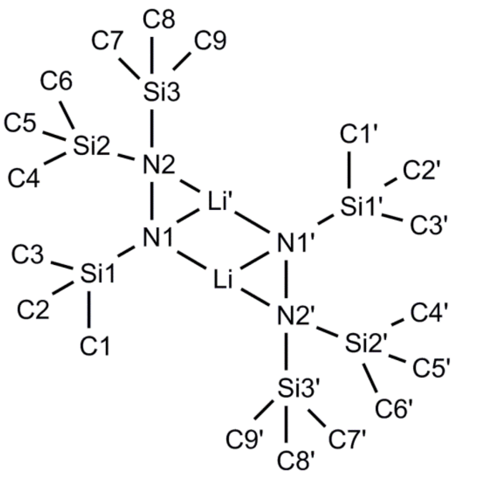

Table S 7: Selected bond lenghts (Å), angles and torsion angles (°) of **[Li4]₂**.

**N1—N2**	1.517 (2)	**N2—N1—Li**	120.91 (17)
**N1—Si1**	1.6968 (19)	**N1—N2—Si2**	114.26 (12)
**N1—Li'**	1.918 (4)	**N1—N2—Si3**	116.00 (13)
**N1—Li**	1.975 (4)	**Si3—N2—Si2**	126.24 (10)
**N2—Si2**	1.7417 (18)	**Si1—N1—N2—Si3**	-68.07 (18)
**N2—Si3**	1.7343 (19)	**Li—N1—N2—Si3**	142.66 (16)
**N2—Li'**	2.146 (4)	**Li—N1—N2—Si2**	-57.1 (2)
**Li—Li'**	2.286 (8)		
**N2—N1—Si1**	124.85 (12)	**∑ (∠ N1)**	322.09
**N2—N1—Li'**	76.33 (16)	**∑ (∠ N2)**	356.5

3 Comparison of selected structural features

Table S 8: Comparison of selected structural features.

Compound	**N1N2**	**Si1N1N2Si3**	**N1Si1**	**Si3N2Si2**
**1** (TTNNTH)[a]	1.458 (3)	96.9 (5)	1.719 (2)	135.7 (3)
$\mathbf{1}^{\cdot+}$ (TTNNTH$^{\cdot+}$)	1.343 (2)	−179.05 (14)	1.822 (2)	122.74 (8)
$\mathbf{2}^{+}$ (TTNNTHH^{+})	1.467 (3)	85.6(2)	1.891 (2)	127.8(1)
$\mathbf{3}^{+}$ (TTNNTTH^{+})[b]	1.499 (5)	104.8 (3)	1.875 (3)	123.6(2)
$\mathbf{4}^{-}$ (TTNT^{-})	1.517 (2)	-68.1 (2)	1.697 (2)	126.2 (1)
$\mathbf{5}^{+}$ **(**TTNNT^{+})[c]	1.254 (2)	−175.33 (11)	1.829 (1)	124.14 (6)

[a] Values of the main part (A) were reported here, [b] The corresponding Si3N2Si was reported here, [c] Data were taken from literature [10].

4 Synthesis of Starting Materials

4.1 Synthesis of *N,N*-Bis(trimethylsilyl)hydrazine, *N,N'*-Bis(trimethylsilyl)hydrazine *and N,N,N'*-Tris(trimethylsilylhydrazine (1).

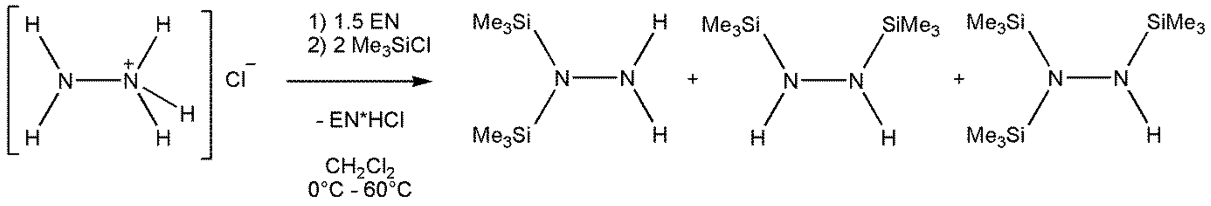

To a stirred suspension of hydrazinium chloride (12 g, 0.18 mol) in dichloromethane (200mL), 1,2-diaminoethane (EN, 15.8 g, 0.26 mol) was added dropwise at 0°C over a period of 10 minutes. The resulting colourless suspension was allowed to warm to ambient temperature within 40 min. To the resulting colourless emulsion, trimethylsilyl chloride (38 g, 0.35 mol) was added dropwise. The reaction mixture was stirred under reflux conditions for further two hours. The resulting suspension was filtered (F4), concentrated to a quarter the volume and stored in the fridge overnight. The resulting suspension was filtered (F4) and separated by fractional vacuum distillations. An isomer mixture of *N,N*-bis(trimethylsilyl)hydrazine and *N,N'*-bis(trimethylsilyl)hydrazine (**A**) 6.3 g (20%) can be separated at 54 °C (12 mbar), a raw product of *N,N,N'*-tris(trimethylsilylhydrazine (**1**) 8.3 g (19%) was collected at 75-87°C (12 mbar). The raw product can be purified by fractional column distillation where **1** was collected at 138 °C (100 mbar) as colorless liquid.

A : **Bp.:** 54 °C (12 mbar). **Anal. calc.** % (found): C, 40.85 (40.63); H, 11.43 (11.07); N, 15.88 (15.78). **^{1}H NMR** (25 °C, $CDCl_3$, 300.13 MHz): δ = 0.02 (s, 18H, C*H*$_3$, $^{1}J(^{1}H-^{13}C)$ = 118 Hz, $^{2}J(^{1}H-^{29}Si)$ = 6.6 Hz), 0.08 (s, 18H, C*H*$_3$, $^{1}J(^{1}H-^{13}C)$ = 118 Hz, $^{2}J(^{1}H-^{29}Si)$ = 6.4 Hz) 2.34 (broad s, 2H, N*H*, $^{1}J(^{1}H-^{15}N)$ = 76 Hz) 2.80 (broad s, 2H, N*H*, $^{1}J(^{1}H-^{15}N)$ = 66 Hz). **^{29}Si NMR** (25 °C, CD_2Cl_2, 59.62 MHz): 5.09 (m). **IR** (ATR, 16 scans): 3344 (w), 2953 (w), 2899 (w), 1571 (w), 1437 (w), 1398 (w), 1292 (w), 1246 (s), 1065 (w), 999 (m), 870 (m), 823 (s), 744 (m), 681 (m), 650 (w), 617 (m). **Raman (**25 °C, 473 nm, 5 mW, 20 sec., 6 acc., cm^{-1}): 3329 (1), 3255 (1), 2943 (5), 2886 (10), 1395 (1), 1232 (1), 1052 (1), 989 (1), 867 (1), 820 (1), 730 (1), 667 (1), 634 (2), 592 (2), 467 (2), 311 (1), 222 (1), 200 (1), 171 (1).

4.2 Synthesis of *N,N,N'*-Tris(trimethylsilyl)hydrazine (1).

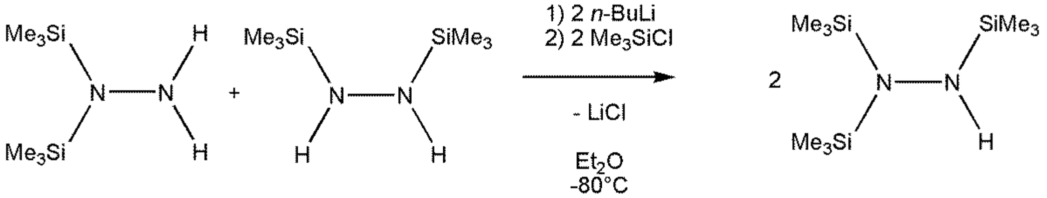

To a stirred solution of bis(trimethylsilyl)hydrazine **A** (10 g, 0.058 mol) in diethyl ether (55mL), a solution of *n*-butyl lithium (2.5 mol/L (hexane), 23 mL, 0.058 mol) was added dropwise at –80 °C over a period of 10 minutes. The resulting colorless solution was slowly warmed up to 0 °C followed by addition of trimethylsilyl chloride (6.9 g, 0.064 mol). The resulting suspension was allowed to warm to ambient temperature within one hour. The solvent was removed *in vacuo* and 30 mL *n*-pentane was added to the resulting precipitate. The resulting suspension was filtered (F4), concentrated to a quarter the volume and stored in the fridge overnight. The resulting suspension was filtered (F4) and purified by fractional vacuum distillation; yield 10g of **1** (72%).

1: **Bp.:** 138 °C (100 mbar); 71 °C (6 mbar). **Anal. calc.** % (found): C, 43.48 (43.23); H, 11.35 (10.82); N, 11.27 (11.16). ^{1}**H NMR** (25 °C, CD_2Cl_2, 400.13 MHz): δ = 0.08 (s, 27H, C*H*$_3$, $^{1}J(^{1}H\text{-}^{13}C)$ = 118 Hz), 2.11 (broad s, 1H, N*H*, $^{1}J(^{1}H\text{-}^{15}N)$ = 79 Hz). ^{13}**C NMR** (25 °C, CD_2Cl_2, 100.63 MHz): 0.85 (s, 3C, Si(*C*H$_3$)$_3$), 1.21 (s, 6C, Si(*C*H$_3$)$_3$). ^{29}**Si NMR** (25 °C, CD_2Cl_2, 79.49 MHz): 5.57 (m, 2Si, *Si*(CH$_3$)$_3$), 6.27 (m, 1Si, *Si*(CH$_3$)$_3$). ^{1}**H,**^{15}**N HMBC** (25 °C, CD_2Cl_2, 400.13 MHz, 40.55 MHz): -328 (s, H*N*Si(CH$_3$)$_3$, $^{1}J(^{1}H\text{-}^{15}N)$ = 79 Hz), -325 (s, *N*(Si(CH$_3$)$_3$)$_2$). ^{1}**H NMR** (25 °C, $CDCl_3$, 300.13 MHz) 0.068 (s, 18H, C*H*$_3$), 0.072 (s, 9H, C*H*$_3$), 2.03 (broad s, 1H, N*H*). **IR** (ATR, 16 scans): 3354 (w), 2953 (w), 2899 (w), 1437 (w), 1398 (w), 1379 (w), 1296 (w), 1246 (s), 1072 (w), 959 (m), 829 (s), 816 (s), 766 (m), 748 (m), 675 (m), 617 (m). **Raman (**25 °C, 632 nm, 12 mW, 10 sec., 10 acc., cm^{-1}): 3348 (1), 2953 (4), 2897 (10), 1585 (1), 1406 (1), 1257 (1), 1072 (1), 959 (1), 831 (1), 739 (1), 675 (2), 645 (7), 602 (3), 512 (3), 432 (1), 368 (2), 288 (1), 226 (2), 192 (2).

Crystals suitable for X-ray crystallographic analysis were obtained, by slow cooling neat **1** to –80 °C.

5 Synthesis of compounds

5.1 Synthesis of *N,N',N'*-tris(trimethylsilyl)hydrazinylium tetrakis(1,1,1,3,3,3-hexafluoro-2-propoxy)aluminate (1·[Al{OCH(CF₃)₂}₄])

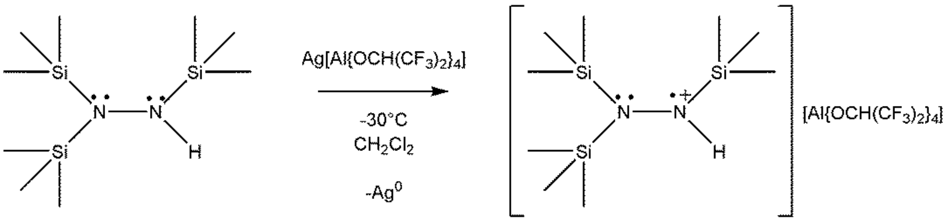

N,N,N'-Tris(trimethylsilyl)hydrazine (**1**) (0.099 g, 0.4 mmol) was added at –70 °C over a period of 5 min to a stirred suspension of silver tetrakis(1,1,1,3,3,3-hexafluoro-2-propoxy)aluminate (0.342 g, 0.43 mmol) in CH_2Cl_2 (6 mL). The resulting colorless suspension was then warmed to –30 °C within 40 min and stirred for further 30 min at this temperature. The resulting orange solution contains a black silver precipitate which was then removed by low temperature filtration (F4, –60 °C). The solution was then concentrated to a volume of 2 mL under vacuum, storage at –80 °C over a period of 12 h, which resulted in the deposition of yellow crystals. Removal of the supernatant by syringe and short drying of the crystals under vacuum at –30 °C yielded **1·[Al{OCH(CF$_3$)$_2$}$_4$]** as yellow crystals, which were stored below –40 °C. An exact yield could not be determined because compound **1·[Al{OCH(CF$_3$)$_2$}$_4$]** decomposes above –20 °C, but the conversion was estimated to be almost complete.

M($C_{21}H_{32}AlF_{24}N_2O_4Si_3$ (**1·[Al{OCH(CF$_3$)$_2$}$_4$]**)= 943.69 g/mol. **Mp.** –20 °C (dec. of the solid); –6 °C (dec. in CH_2Cl_2 solution) **Anal. calc.** % (found): not determined due to its low decomposition temperature **^{1}H NMR** (–50 °C, CD_2Cl_2, 400.13 MHz): δ = 4.47 (broad, 4H, CH_{anion}, $\nu_{1/2}\approx$ 56 Hz), 11.9 (v. broad, ≈28H, H_{cation} , $\nu_{1/2}\approx$ 5300 Hz) **^{1}H NMR** (–30 °C, CD_2Cl_2, 400.13 MHz): δ = 4.48 (broad, 4H, CH_{anion}, $\nu_{1/2}\approx$ 38 Hz), 10.8 (v. broad, ≈28H, H_{cation}, $\nu_{1/2}\approx$ 4300 Hz), **^{1}H NMR** (0 °C, CD_2Cl_2, 400.13 MHz): δ = 4.51 (broad, 4H, CH_{anion}, $\nu_{1/2}\approx$ 26 Hz), 9.8 (v. broad, ≈28H, H_{cation}, $\nu_{1/2}\approx$ 5100 Hz), **^{1}H NMR** (24 °C, CD_2Cl_2, 400.13 MHz): δ = 4.53 (broad, 4H, CH_{anion}, $\nu_{1/2}\approx$ 23 Hz), 8.8 (v. broad, ≈28H, H_{cation}, $\nu_{1/2}\approx$ 7600 Hz) **^{13}C NMR** (–50 °C, CD_2Cl_2, 100.63 MHz): 69.9 (broad s, CH_{anion}) 122.4 (q, CF_3, $^1J(^{13}C-^{19}F)$ = 280 Hz). **^{29}Si NMR** (–50 °C, CD_2Cl_2, 79.49 MHz): not observed. **IR** (ATR): not determined due to its low decomposition temperature. **Raman** (–80 °C, 473 nm, 5 mW, 20 sec., 8 acc., cm^{-1}): 3303 (1), 2965 (2), 2909 (3), 2714 (1), 2633 (1), 1670 (1), 1412 (1), 1371 (1), 1321 (10), 1277 (1), 1257 (1), 1200 (1), 1186 (1), 1091 (1), 850 (1), 810 (1), 756 (1), 725 (1), 688 (1), 642 (3), 622 (1), 586 (1), 563 (1), 528 (1), 514 (1), 480 (1), 343 (1), 323 (1), 304 (1), 289 (1), 262 (1). **EPR** (-73 °C, CH_2Cl_2): g= 2.0045, A_H=11.7 G, 2x A_N=9.1G, line width ΔB=1.5G.

5.2 Attempted Synthesis of *N,N',N'*-tris(trimethylsilyl)hydrazinylium salts

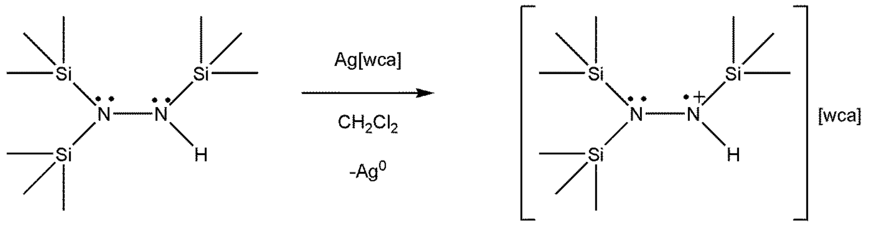

According to the described reaction procedure for **1·[Al{OCH(CF$_3$)$_2$}$_4$]** we were interested in the formation of salts bearing the hydrazinylium radical cation with different counter anions **1·[wca]**. In a series of experiments, we examined the reaction behavior of silver salts with different weakly coordinating anions ([wca]). In a general procedure the silver salts were dissolved in cooled dichloromethane (–80 °C) at this temperature *N,N,N'*-tris(tri-methylsilyl)hydrazine (**1**) was added dropwise. In all cases the reaction mixtures show no observable reaction at this temperature. In the most cases slow warming of these mixtures led to the deposition of a blackish silver precipitate. The observed reaction temperatures and the identified reaction products are listed in ***Table S 9***. It should be mentioned here that, except in the case of [Al(OR$_F$)$_4$], no uniform reaction product could be isolated. With luck, a few crystals could be isolated that were suitable for single crystal structure determination. For this reason, no further analytical data are provided for the Hydrazinium salts.

Table S 9: Summary of the attempted Synthesis of [(Me$_3$Si)$_2$NN(H)SiMe$_3$][wca] salts.

**[wca]$^-$**	**T$_{reaction}$ / °C**	**identified product**
[GaCl$_4$]$^-$	no reaction	-
[B(C$_6$H$_5$)$_4$]$^-$	no reaction	-
[CHB$_{11}$H$_5$Br$_6$]$^-$	+10 °C	Hydrazinium salt (**2**$^+$)
[B(C$_6$F$_5$)$_4$]$^-$	–30 °C	Hydrazinium salt (**3**$^+$) contains approximately 6% hydrazinium-yl radical cation (**1**$^{\cdot+}$)
[F$_3$CSO$_3$]$^-$	–35 °C	Hydrazinium and ammonium salts
[Al(OR$_F$)$_4$]$^-$	–50 °C	Hydrazinium-yl radical cation (**1**$^{\cdot+}$)
[BF$_4$]$^-$	–50 °C	unidentified compounds
[SbF$_6$]$^-$	–60 °C	Hydrazinium-yl radical cation (**1**$^{\cdot+}$)

6 NMR Spectra

6.1 NMR Spectra of 1

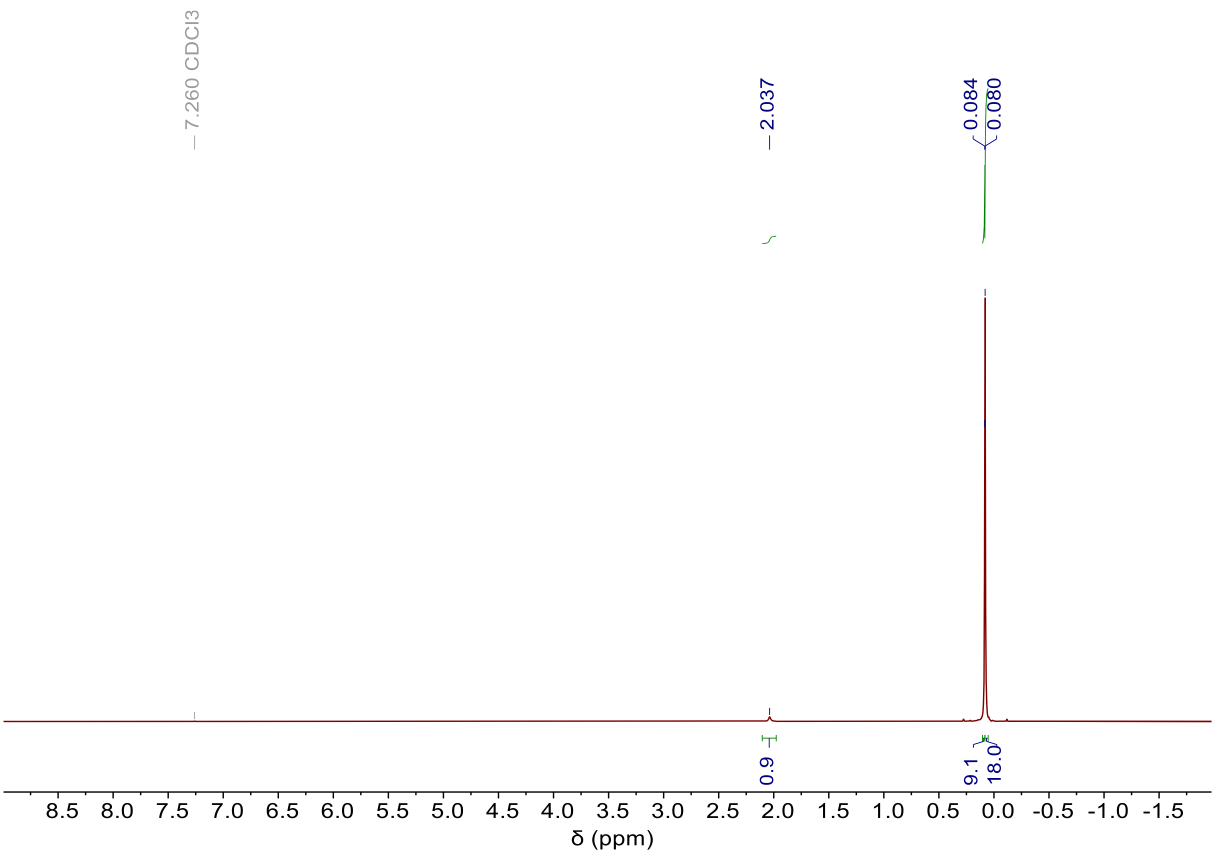

Figure S 1: Representation of the **^{1}H NMR** (25 °C, $CDCl_3$, 300.13 MHz) spectrum of pure compound **1**. Analysis pure **1** was obtained via a slit-tube column distillation of combined pre-distillates from several syntheses. An unusually large amount of about 60 mg of **1** was chosen here to check purity and therefore the chemical shifts differ slightly from those reported above. For the follow-up chemistry studies, **1** was used with samples of this purity.

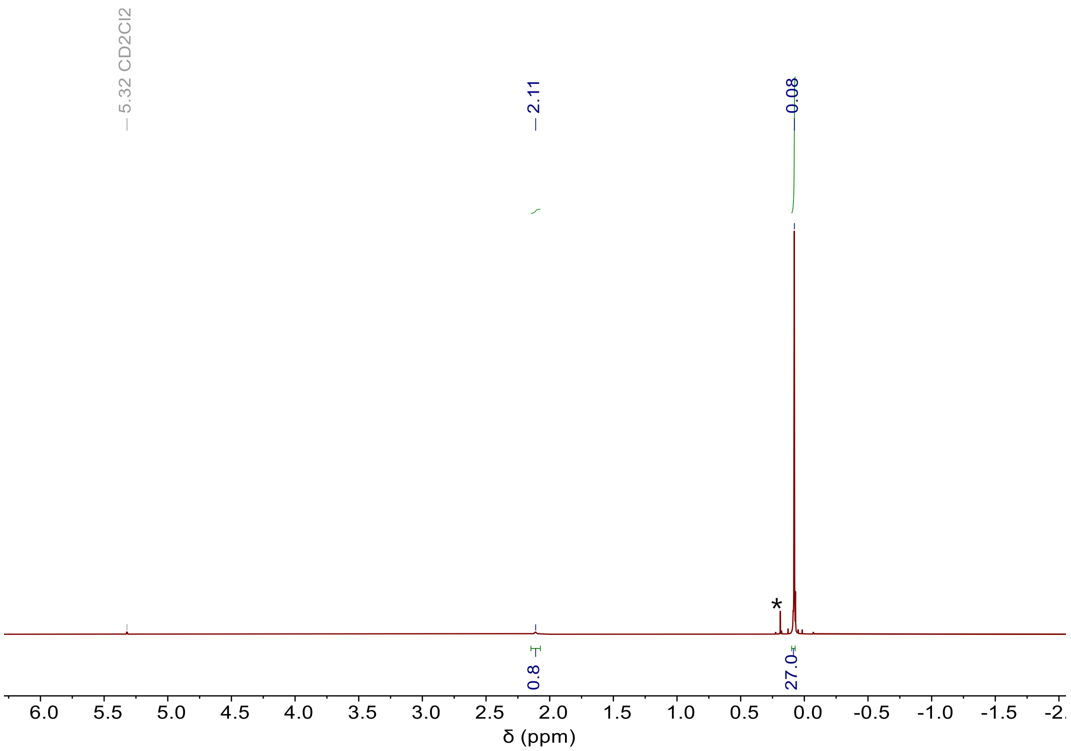

Figure S 2: Representation of the **^{1}H NMR** (25 °C, CD_2Cl_2, 300.13 MHz) spectrum of almost pure compound **1** before slit-tube distillation. The asterisk marks minor portions of unidentified impurities.

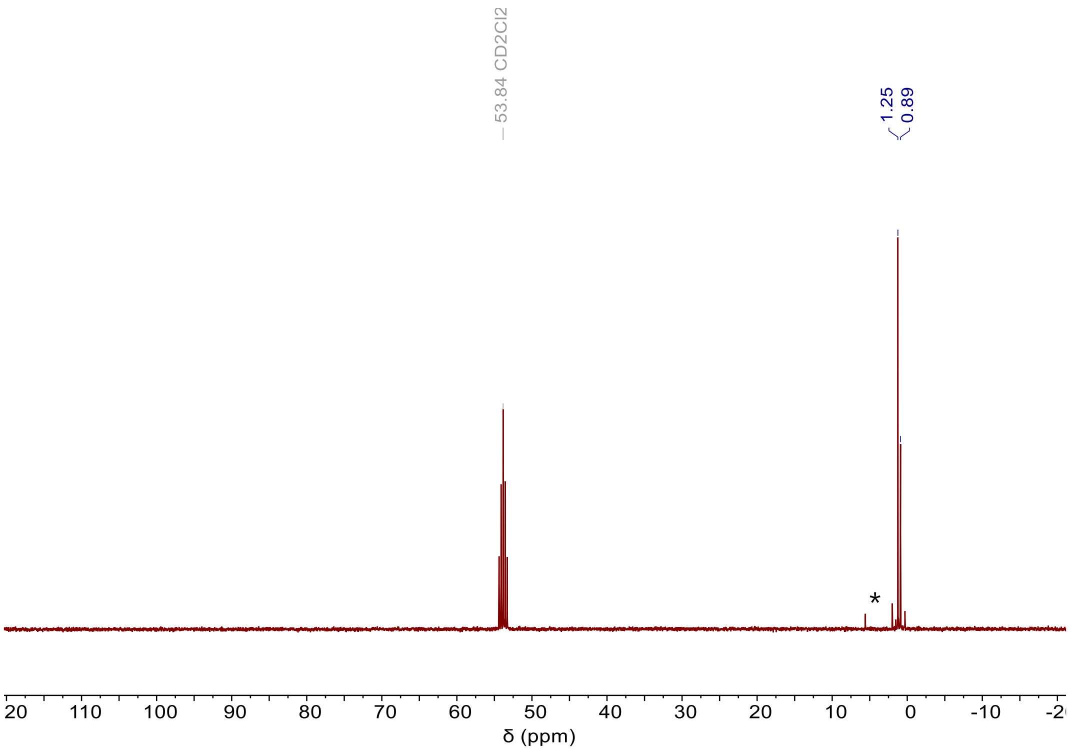

Figure S 3: Representation of the **^{13}C NMR** (25 °C, CD_2Cl_2, 100.63 MHz) spectrum of almost pure compound **1** before slit-tube distillation. The asterisk marks minor portions of unidentified impurities.

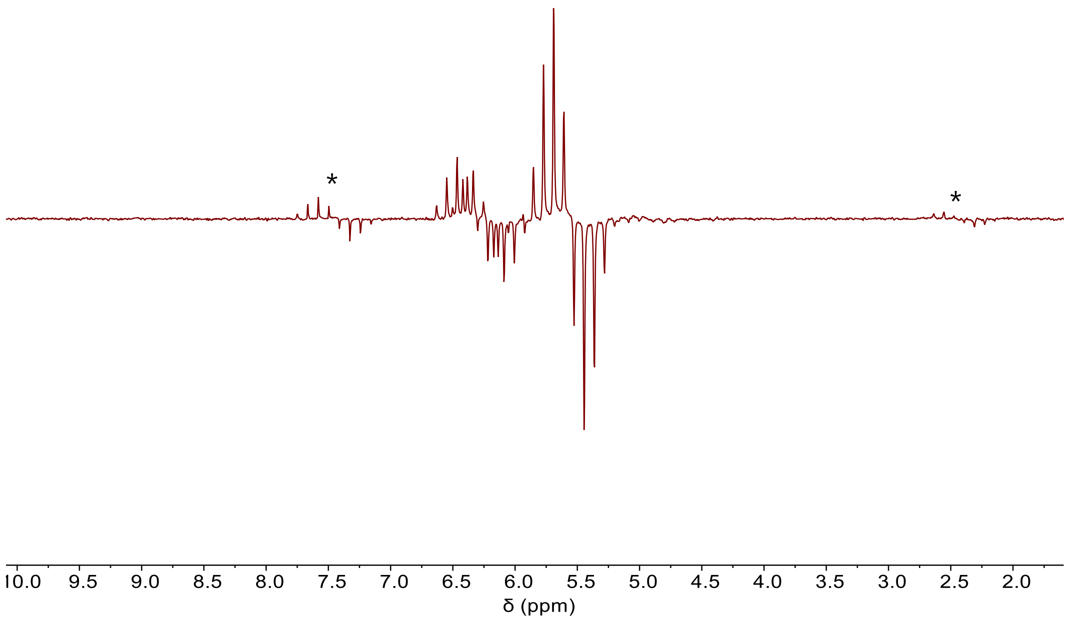

Figure S 4: Representation of the **^{29}Si-inept NMR** (25 °C, CD_2Cl_2, 79.49 MHz) spectrum of almost pure compound **1** before slit-tube distillation. The asterisk marks minor portions of unidentified impurities.

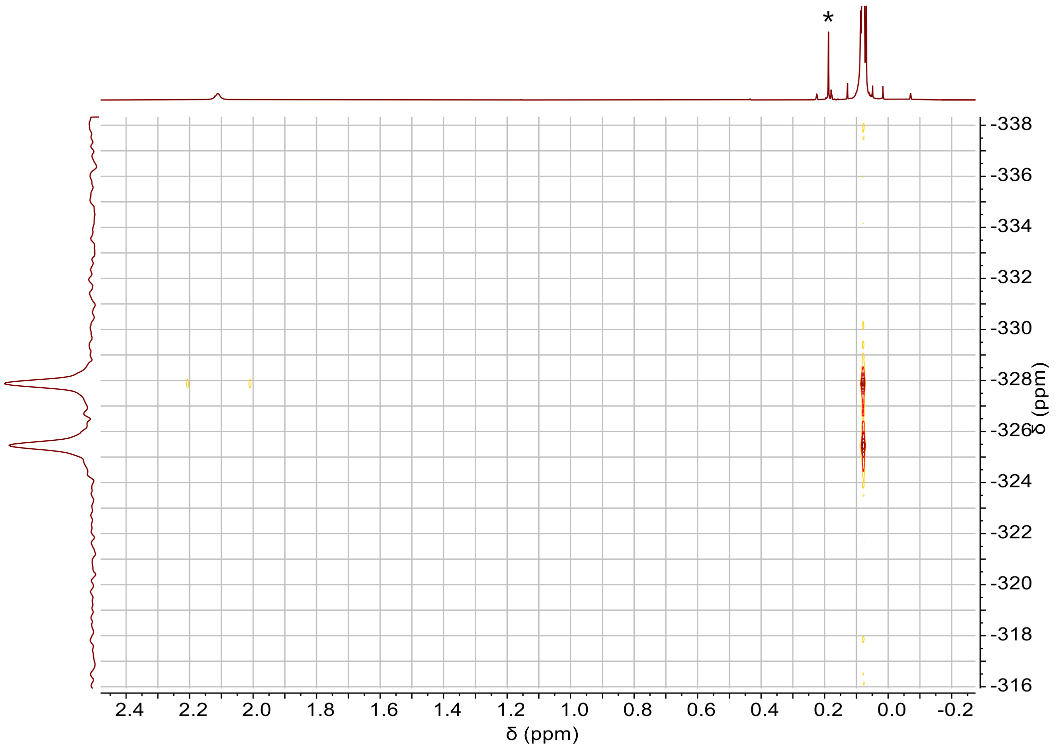

Figure S 5: Representation of the **^{1}H,^{15}N HMBC** (25 °C, CD_2Cl_2, 400.13 MHz, 40.55 MHz) spectrum of almost pure compound **1** before slit-tube distillation. The asterisk marks minor portions of unidentified impurities.

6.2 NMR Spectra of 1[Al{OCH(CF_3)$_2$}$_4$]

For NMR analysis, isolated crystals of **1[Al{OCH(CF_3)$_2$}$_4$]** were dissolved at a temperature below -40°C in CD_2Cl_2 and transferred to a cooled NMR tube at this temperature. As far as possible, the sample was kept strictly below the estimated decomposition temperature.

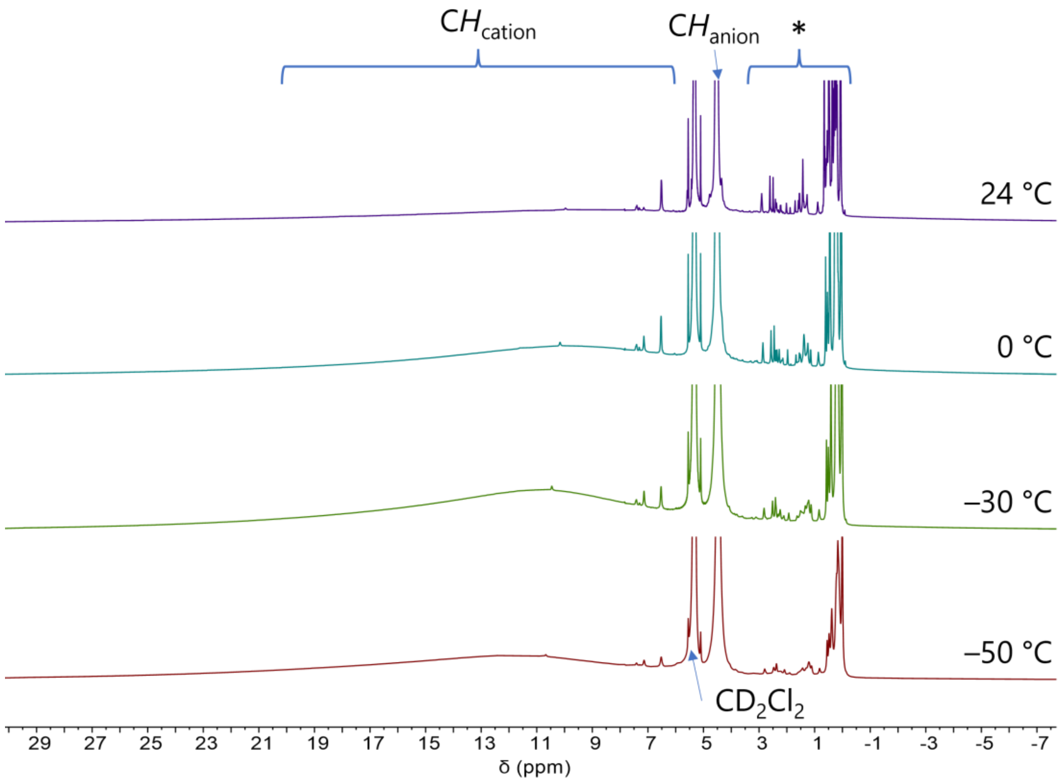

Figure S 6: Representation of the variable temperature [1]**H NMR** (CD_2Cl_2, 400.13 MHz) experiment of **1[Al{OCH(CF_3)$_2$}$_4$]**. The asterisk marks unidentified decomposition products.

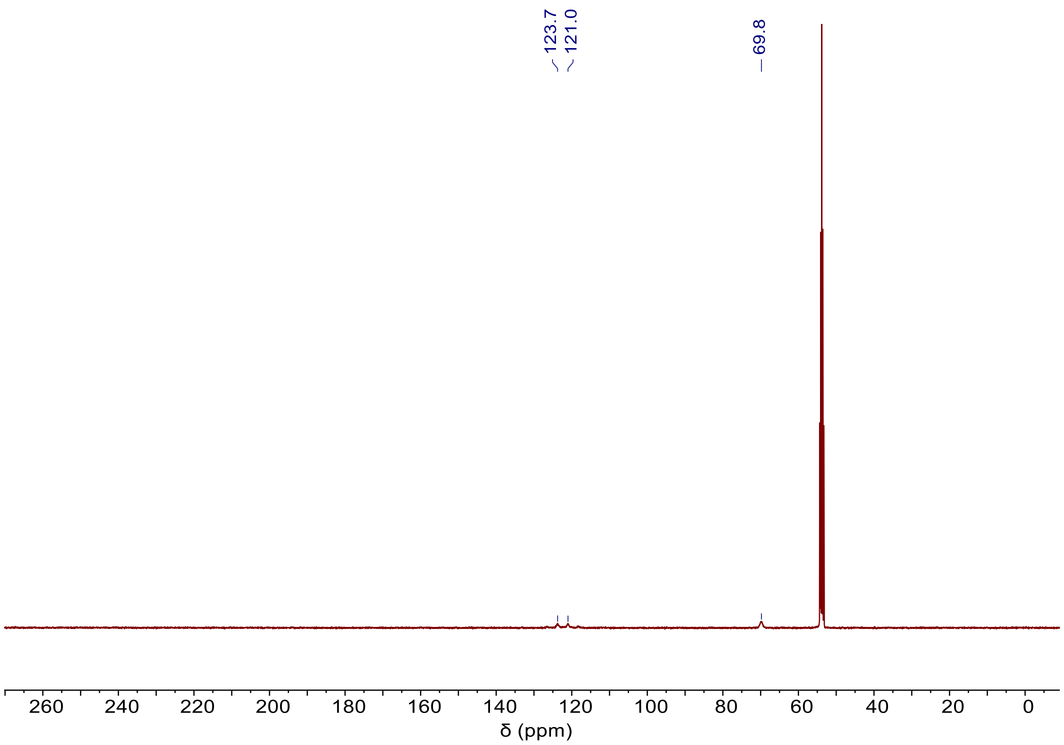

Figure S 7: Representation of the **^{13}C NMR** (–50 °C, CD_2Cl_2, 100.63 MHz) spectrum of compound **1[Al{OCH(CF_3)$_2$}$_4$].**

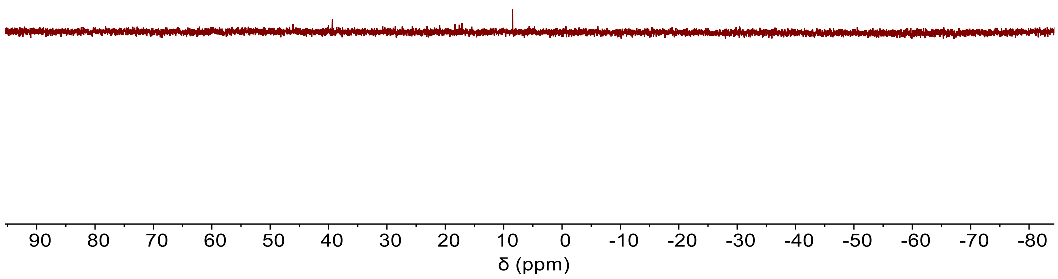

Figure S 8: Representation of the **^{29}Si NMR** (–50 °C, CD_2Cl_2, 79.49 MHz) spectrum of compound **1[Al{OCH(CF_3)$_2$}$_4$]** only shows resonances of unidentified decomposition products.

7 IR, Raman and Vis Spectra

The assignments of the vibrational modes were carried out with the help of quantum mechanical calculations at M06/def2tzvpp level of theory. The reported calculated vibrational modes are uncorrected.

Table S 10: Assignment of selected $(Me_3Si)_2NN(H)SiMe_3$ (**1**) vibrations.

Vibration	Atoms	$\nu_{calc.}$	$\nu_{exp.IR}$	$\nu_{exp.Raman}$
ν	NH	3521	3354	3348
$\nu_{out\text{-}of\text{-}phase}$	CH_3	3143-3108	2953	2953
$\nu_{in\text{-}phase}$	CH_3	3044-3027	2899	2897
$\delta_{in\text{-}plane}$	NH	1400	-	-
$\delta_{out\text{-}of\text{-}phase}$	CH_3	1468-1427	1437-1296	1406
$\delta_{in\text{-}phase}$	CH_3	1279-1260	1246	1257
ν	NN	1140	1072	1072
wagging	CH_3	884-654	816-617	739-602
$\delta_{out\text{-}plane}$	NH	427	-	-

Table S 11: Assignment of selected $[(Me_3Si)_2NN(H)SiMe_3]^+$ (**1**$^{\cdot+}$) vibrations.

Vibration	Atoms	$\nu_{calc.}$	$\nu_{exp.IR}$	$\nu_{exp.Raman}$
ν	NH	3477	-	3303
$\nu_{out\text{-}of\text{-}phase}$	CH_3	3176-3111	-	2965-2909
$\nu_{in\text{-}phase}$	CH_3	3054-3033	-	2714
$\delta_{in\text{-}plane}$	NH	1525	-	1412
$\delta_{out\text{-}of\text{-}phase}$	CH_3	1468-1419	-	1371
ν	NN	1394	-	1321
$\delta_{in\text{-}phase}$	CH_3	1296-1276	-	1200-1186
wagging	CH_3	882-654	-	850-622
$\nu_{in\text{-}phase}$	$Si\text{-}CH_3$	646-630	-	642
$\delta_{out\text{-}plane}$	NH	614	-	-

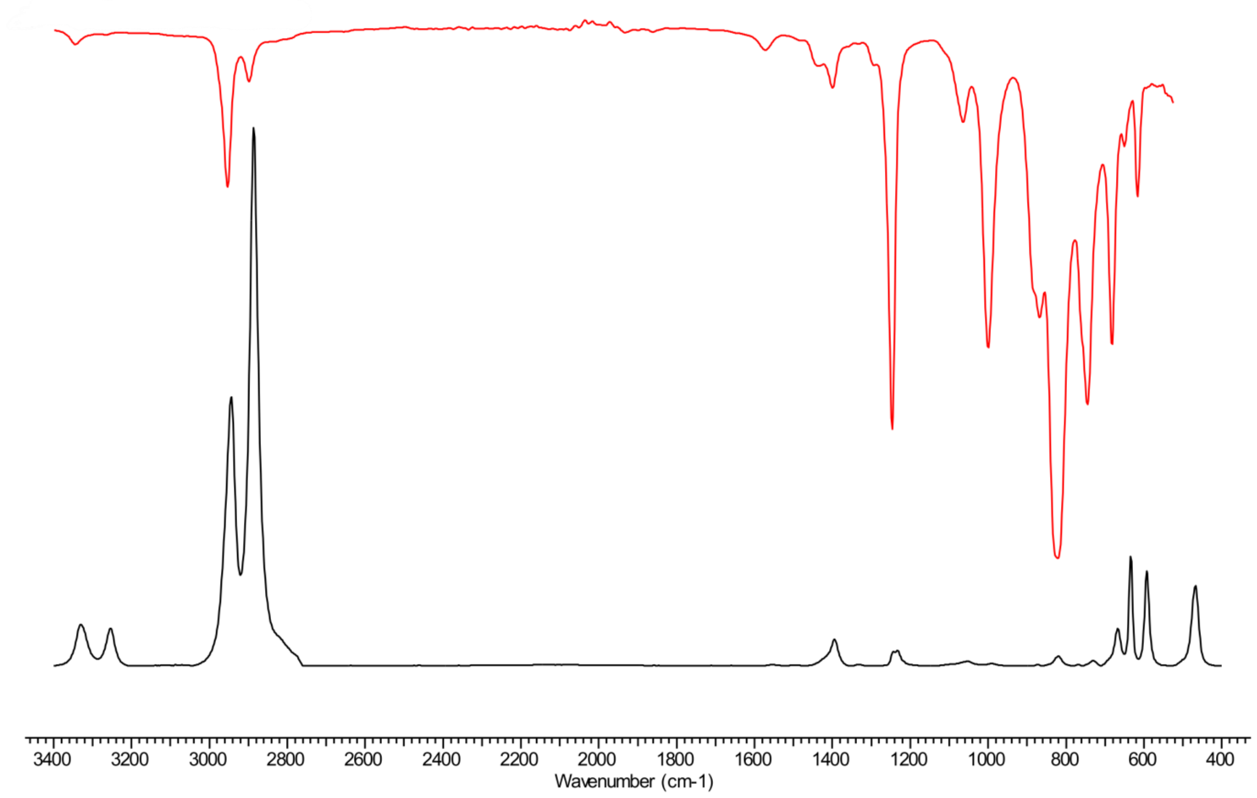

Figure S 9: IR (red) and Raman (black) spectrum of bis(trimethylsilyl)hydrazine (**A**) isomer mixture.

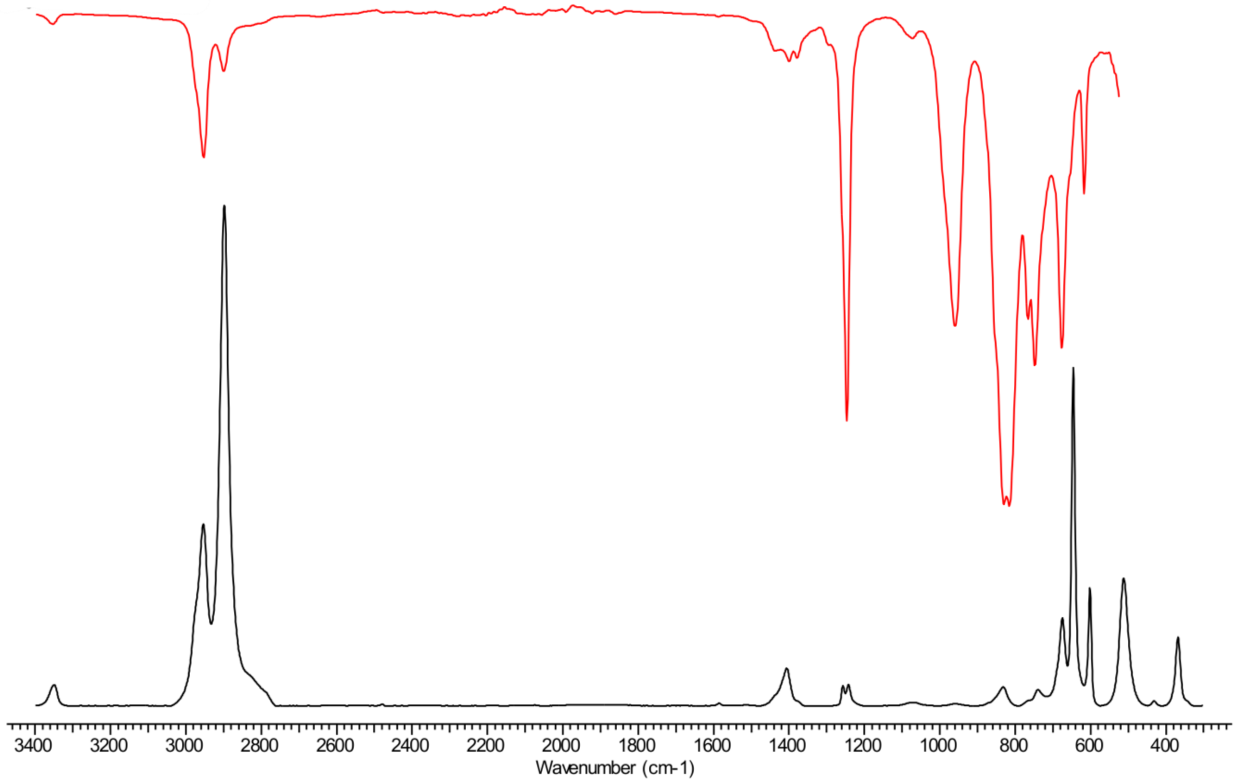

Figure S 10: IR (red) and Raman (black) spectrum of tris(trimethylsilyl)hydrazine (**1**).

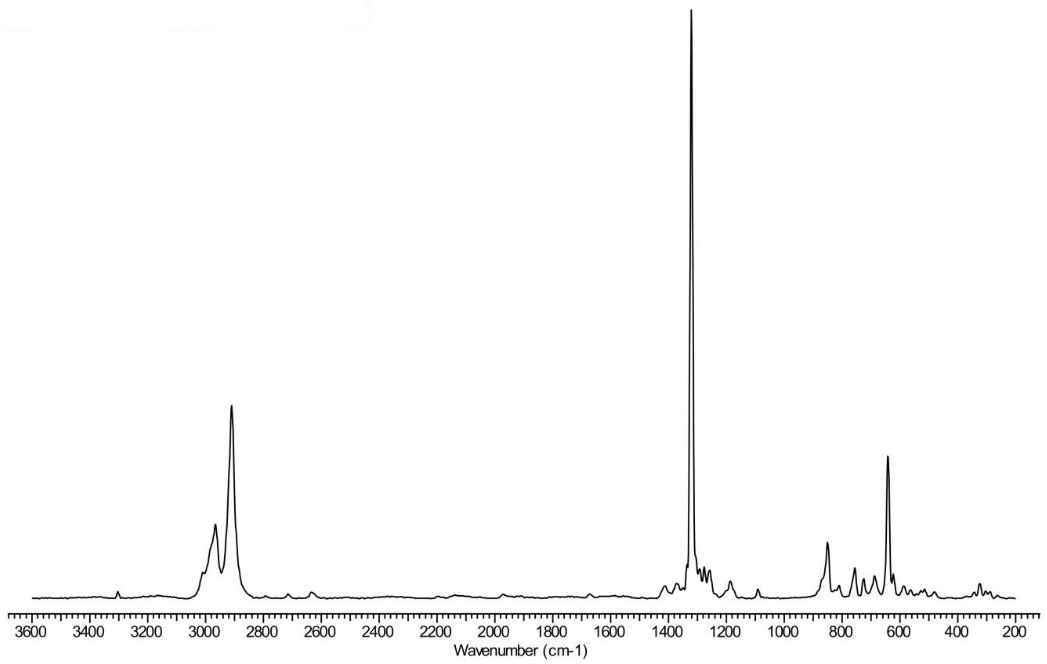

Figure S 11: Raman spectrum of (**1**·**[Al{OCH(CF$_3$)$_2$}$_4$]**) measured at –80 °C.

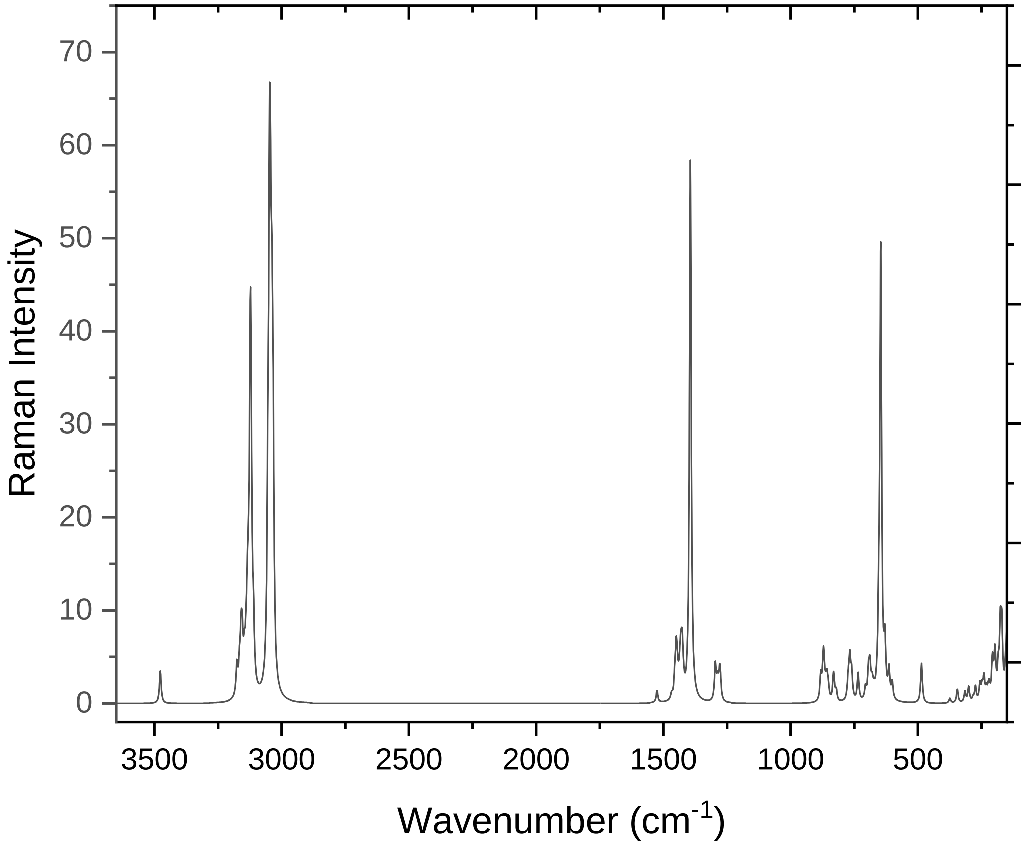

Figure S 12: Calculated RAMAN Spectrum of **1**$^{\cdot+}$ cation (level of theory M06/def2tzvpp).

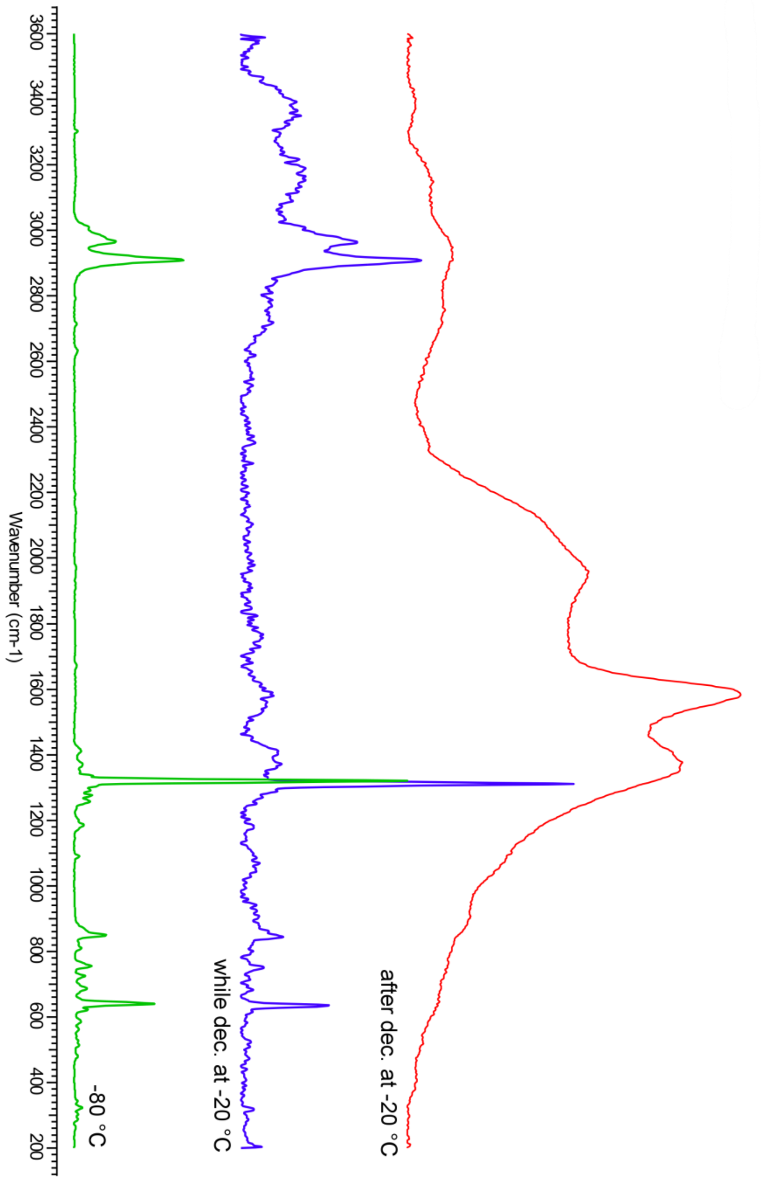

Figure S 13: Raman spectra of **1·[Al{OCH(CF_3)$_2$}$_4$]** measured at –80 °C (green), decomposition of **1·[Al{OCH(CF_3)$_2$}$_4$]** at –20 °C during dec. (blue), after dec. (red) with 432 nm excitation source.

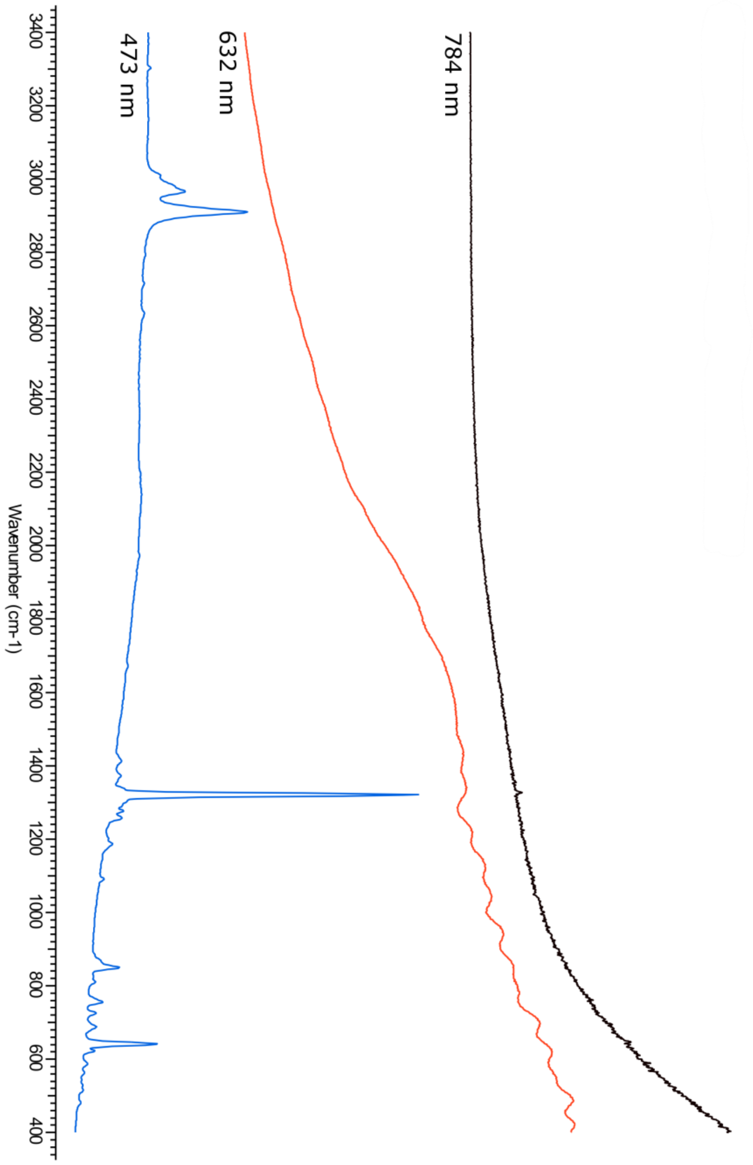

Figure S 14:Raman spectra of **1**·**[Al{OCH(CF_3)$_2$}$_4$]** measured at –80 °C show the Resonance Raman Effect with best results using 473 nm Laser excitation.

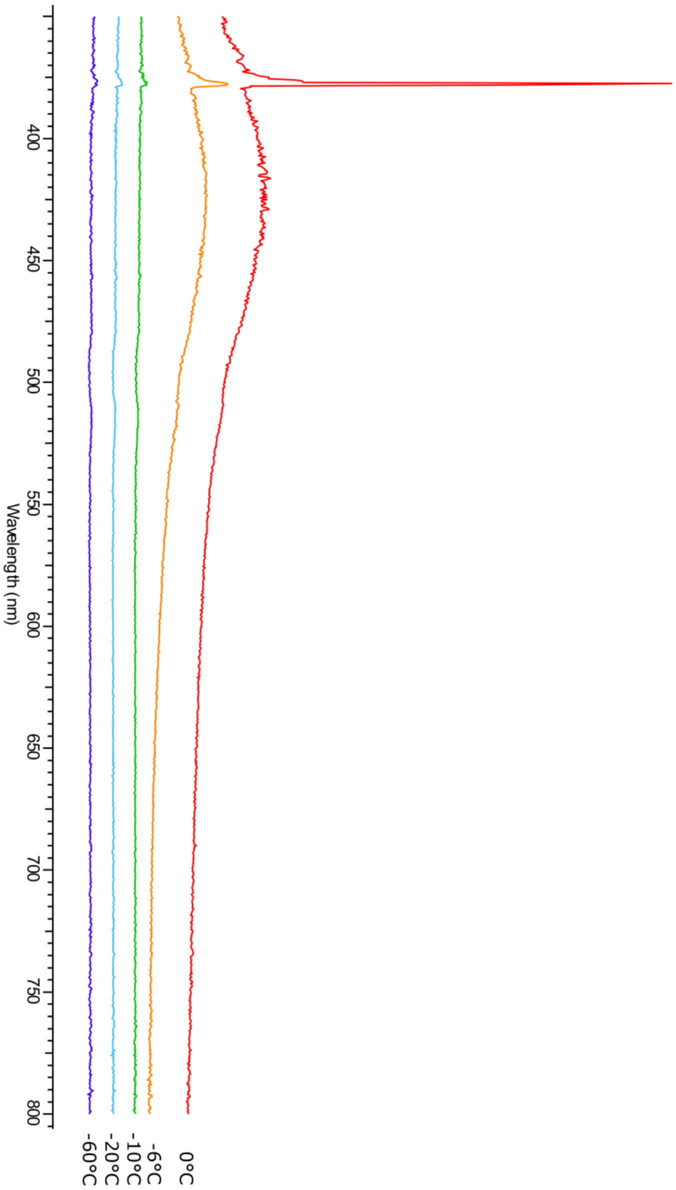

Figure S 15: Vis spectra of **1**·**[Al{OCH(CF_3)$_2$}$_4$]** in CH_2Cl_2 solution measured at different temperatures show the decomposition of **1**·**[Al{OCH(CF_3)$_2$}$_4$]** into an unidentified yellow/orange product.

8 EPR – Spectrum of 1·[Al{OCH(CF_3)$_2$}$_4$]

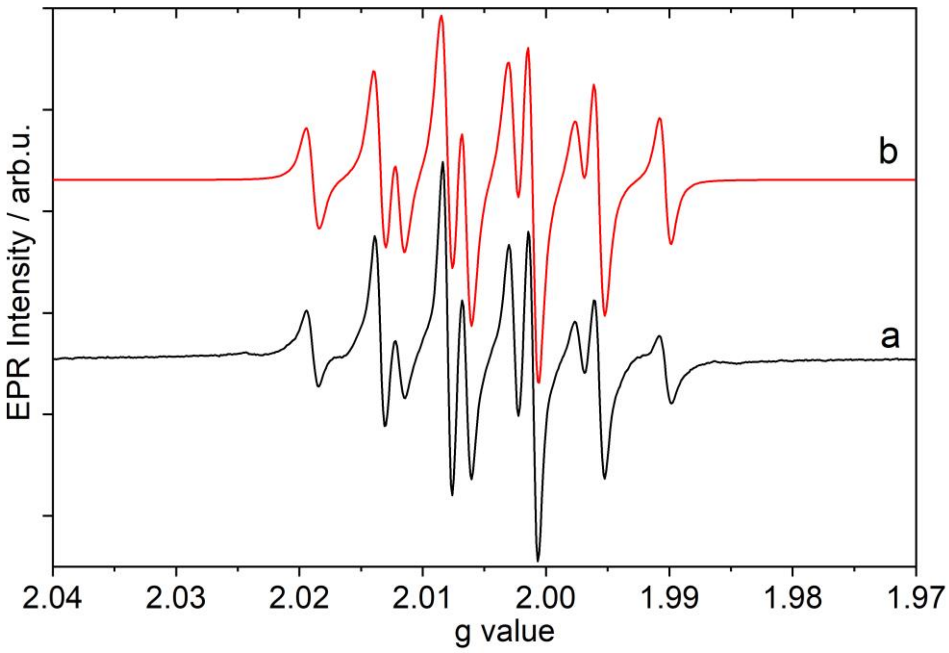

Figure S 16: EPR-Spectrum of **1·[Al{OCH(CF_3)$_2$}$_4$]** at 200K in CH_2Cl_2, a) measured and b) simulated using the following parameter g= 2.0045, A_H=11.7 G, 2x A_N=9.1G, line width ΔB=1.5G.

For EPR analysis, isolated crystals were dissolved at a temperature below -40°C and transferred to a cooled EPR tube at this temperature. As far as possible, the sample was kept strictly below the estimated decomposition temperature, at 300K/RT the compound is not stable, thus the EPR measurements were performed at 200K. The simulation was performed using the EPRsim32 Software developed by Sojka *et. al.*[11].

9 Computational Details

9.1 General remarks

All calculations were carried out with the Gaussian 09 package of molecular orbital programs.[12] The structures (pre-optimization) were optimized within the DFT approach at the M06[13] level with an aug-cc-pVDZ basis set.[14] Vibrational frequencies were also computed, to include zero-point vibrational energies in thermodynamic parameters and to characterize all structures as minima on the potential energy surface. In a second step we performed a small bench mark between the DFT approaches M06 and PBE0 with the def2tzvpp[15] basis set to find the method of choice for further bond analysis and to investigate the influence of an empirical dispersion correction (gd3 or gd3bj)[16] on the optimized the structures of the neutral and cationic dinitrogen fragments (**1**, $\mathbf{1}^{\cdot+\cdot}$, $\mathbf{2}^{+}$, $\mathbf{3}^{+}$). On this basis, the structures from the M06/def2tzvpp optimizations show the largest and best agreement. Therefore, unless otherwise stated, all computational data discussed refer to this level of theory. The bond angles in all these molecules are also rather independent of the choice of basis sets but strongly depend on the experimental environment. [17] A natural bond orbital analysis (NBO 6.0) was performed at the same level, to study the charge distribution, bond polarization and hybridization effects.

QT-AIM, ELF and Wiberg bond index calculations/visualization were performed using MultiWfn 3.5 employing Gaussian09 formatted checkpoint files.[18] For the visualization of 3D-quantum chemical results we used GaussView 6.1.1.[19] In addition to the Supporting Information, we provide a multi-structure xyz file with all calculated molecules.

For a better understanding and a more intuitive view of the calculated 3D structures, we strongly recommend using this file e.g. with the free program MERCURY.[20]

It should be emphasized that the computation was carried out for a single, isolated (gas-phase) species and in the case of ($\mathbf{1}^{\cdot+}$, $\mathbf{2}^{+}$, and $\mathbf{3}^{+}$*) only for the cation. There may well be significant differences among gas-phase, solution, and solid-state data.*

9.2 Optimized Structures

In this section we present the details of the former described benchmark calculations to find the method of choice to correctly describe the binding situation in the dinitrogen containing fragments of the here investigated molecules. We optimized the structures of the neutral and cationic dinitrogen fragments (**1**, **1**$^{\cdot+}$, **2**$^{+}$, and **3**$^{+}$) on the DFT approaches M06 and PBE0 with the def2tzvpp[15] basis set and compared the goodness of the description of the binding situations in comparison with the experimental data from the single-crystal X-ray diffraction (SC-XRD) measurements in detail (***Table S 12*** - ***Table S 16***). Moreover, we optimized these structures considering an empirical dispersion correction in both cases and included these results in our comparison (gd3 or gd3bj).[16] On this basis, the structures from the M06/def2tzvpp optimizations show the largest and best agreement. Therefore, unless otherwise stated, all computational data discussed refer to this level of theory.

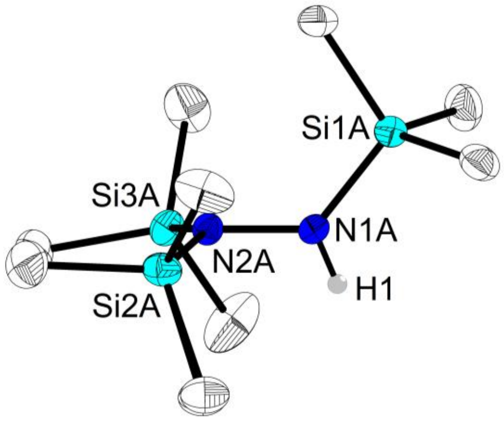

Figure S 17: Molecular structure of compound **1**. Thermal ellipsoids correspond to 30% probability. Hydrogen atoms (except H1) and the second position of the disordered molecule are omitted for clarity.

Table S 12: Comparison of selected binding parameters of the SC-XRD measurement of (**1**) and four different optimized structures. The italicized values are the differences between experimental and calculated values. Light green values show the best matches, yellow highlighted are good matches and pale red show moderate matches.

Value	SC-XRD[a]	M06		M06/gd3		pbe0		pbe0/gd3bj	
N1-N2	1.458	1.437	*-0.021*	1.437	*-0.021*	1.435	*-0.023*	1.433	*-0.025*
N1-Si1	1.719	1.737	*0.018*	1.737	*0.018*	1.742	*0.023*	1.739	*0.020*
N2-N1-Si1	128.1	126.85	*-1.25*	126.78	*-1.32*	128.23	*0.130*	126.24	*-1.86*
Si3-N2-N1-Si	96.9	89.59	*-7.31*	89.78	*-7.12*	81.38	*-15.52*	79.47	*-17.43*

[a] Values for A part of the disordered molecule.

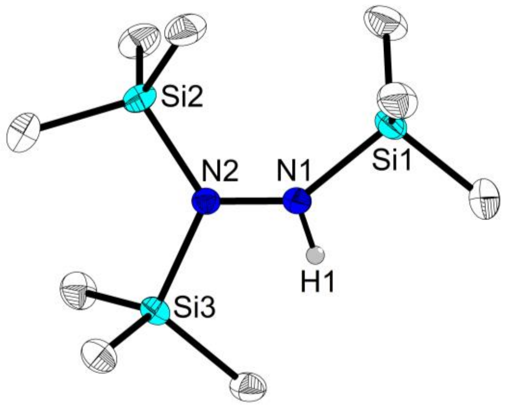

Figure S 18: Molecular structure of radical cation **1**$^{\cdot+}$. Thermal ellipsoids correspond to 30% probability. Hydrogen atoms (except H1) and the counter anion are omitted for clarity.

Table S 13: Comparison of selected binding parameters of the SC-XRD measurement of (**1**$^{\cdot+}$) and four different optimized structures. The italicized values are the differences between experimental and calculated values. Light green values show the best matches, yellow highlighted are good matches and pale red show moderate matches.

Value	SC-XRD	M06		M06/gd3		pbe0		pbe0/gd3bj	
N1-N2	1.343	1.332	*-0.011*	1.332	*-0.011*	1.332	*-0.011*	1.33	*-0.013*
N1-Si1	1.8224	1.839	*0.0166*	1.838	*0.0156*	1.837	*0.0146*	1.83	*0.0076*
N2-N1-Si1	140.43	140.38	*-0.05*	140.46	*0.03*	140.71	*0.28*	140.05	*-0.38*
Si3-N2-N1-Si	179.05	173.09	*-5.96*	172.49	*-6.56*	178.45	*-0.6*	176.59	*-2.46*

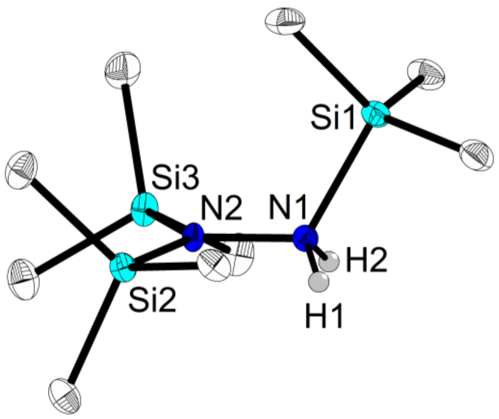

Figure S 19: Molecular structure of cation **2**⁺. Thermal ellipsoids correspond to 30% probability. Hydrogen atoms (except H1 and H2) and the counter anion are omitted for clarity.

Table S 14: Comparison of selected binding parameters of the SC-XRD measurement of (**2**⁺) and four different optimized structures. The italicized values are the differences between experimental and calculated values. Light green values show the best matches, yellow highlighted are good matches and pale red show moderate matches.

Value	SC-XRD	M06		M06/gd3		pbe0		pbe0/gd3bj	
N1-N2	1.467	1.444	*-0.023*	1.443	*-0.024*	1.442	*-0.025*	1.438	*-0.029*
N1-Si1	1.891	1.915	*0.024*	1.915	*0.024*	1.917	*0.026*	1.908	*0.017*
N2-N1-Si1	119.42	119.38	*-0.04*	119.29	*-0.13*	121.02	*1.6*	119.69	*0.27*
Si3-N2-N1-Si	85.6	85.15	*-0.45*	85.12	*-0.48*	84.79	*-0.81*	84.59	*-1.01*

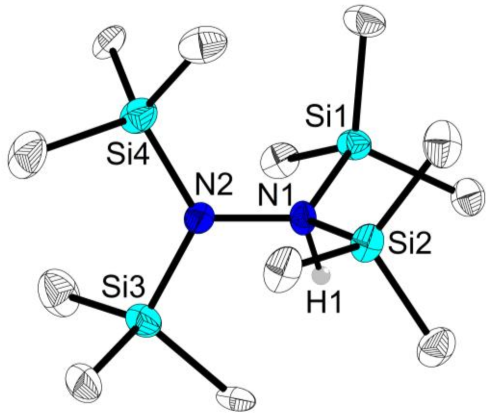

Figure S 20: Molecular structure of cation **3⁺**. Thermal ellipsoids correspond to 30% probability. Hydrogen atoms (except H1) and the counter anion are omitted for clarity.

Table S 15: Comparison of selected binding parameters of the SC-XRD measurement of (**3⁺**) and four different optimized structures. The italicized values are the differences between experimental and calculated values. Light green values show the best matches, yellow highlighted are good matches and pale red show moderate matches.

Value	SC-XRD	M06		M06/gd3		pbe0		pbe0/gd3bj	
N1-N2	1.499	1.465	*-0.034*	1.464	*-0.035*	1.465	*-0.034*	1.459	*-0.040*
N1-Si1	1.875	1.892	*0.017*	1.891	*0.016*	1.900	*0.025*	1.887	*0.012*
N2-N1-Si1	116.5	115.49	*-1.01*	115.47	*-1.03*	116.04	*-0.46*	115.65	*-0.85*
Si3-N2-N1-Si	104.8	107.21	*2.41*	107.3	*2.5*	107.95	*3.15*	107.83	*3.03*

Table S 16: Conclusion of the four compared structures. The DFT M06 method without empirical dispersion shows over 16 values the best matches and less moderate matches.

	Best matches	good matches	moderate matches
M06	8	6	2
M06/gd3	4	9	2
pbe0	5	3	8
pbe0/gd3bj	3	5	8

9.3 Bonding analysis

In this chapter, we summarize the most important results from a series of different bonding analyses to gain a deeper insight into the electronic situation of the hydrazine, hydrazinyl and hydrazinium structures. These calculations were performed on the molecular structures optimized at M06/def2tzvpp level of theory.

Based on the calculated Wiberg Bond Indices (WBI) and Localized Molecular Orbital Bond Orders (LMO-BO) of the NN bonds in the investigated compounds the NN bonds in **1**, $\mathbf{2}^{+}$ and $\mathbf{3}^{+}$ are best described as classical single σ-bonds (***Table S 17***). In contrast, the diazenium cation $\mathbf{5}^{+}$ NN bond is best described by a double bond consisting of a classical σ- and π-bond system. The values in between both types of bond description for the here investigated hydrazinyl radical cation $\mathbf{1}^{\cdot+}$ are well in line with the description of a three electron π-bond and a classical σ-bond in the NN unit.

Table S 17: Calculated Wiberg Bond Indices (WBI) and Localized Molecular Orbital Bond Orders (LMO-BO) of the NN bonds in the investigated compounds.

Molecule/Cation	WBI (NN)	LMO-BO (NN)
**1**	1.20	0.99
$\mathbf{1}^{\cdot+\,a}$	1.68	1.44
$\mathbf{2}^{+}$	1.17	0.90
$\mathbf{3}^{+}$	1.16	0.91
$\mathbf{5}^{+}$	2.29	1.78

a = Values for alpha and beta spin were summed.

9.3.1 NBO analysis and Natural Molecular Orbitals NLMOs

9.3.1.1 NBO / NLMO Analysis of 1

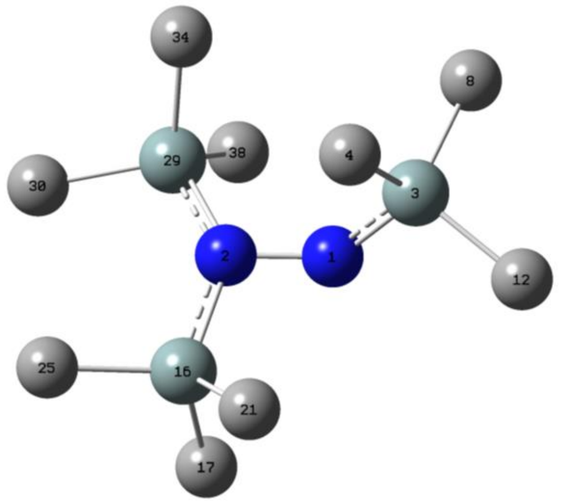

Figure S 21: Numbering of **1** hydrogens are omitted for clarity H42 is connected at N1.

Table S 18: Summary of selected NBO analysis of **1**.

27. (1.89858) LP (1) N 1<br><br>s(1.08%)p91.45(98.87%)d 0.02(0.02%) f 0.02(0.02%)	28. (1.85762) LP (1) N 2<br><br>s(1.84%)p53.18(98.10%)d 0.02(0.04%) f 0.01(0.02%)
29. (1.98947) BD (1) N 1- N 2<br><br>(50.46%) 0.7103* N 1 s(29.28%)p 2.41(70.65%)d 0.00(0.05%) f 0.00(0.02%)<br><br>(49.54%) 0.7039* N 2 s(22.55%)p 3.43(77.35%)d 0.00(0.08%) f 0.00(0.01%)	30. (1.97966) BD (1) N 1-Si 3<br><br>(81.02%) 0.9001* N 1 s(43.92%)p 1.27(55.91%)d 0.00(0.16%) f 0.00(0.01%)<br><br>(18.98%) 0.4356*Si 3 s(21.36%)p 3.64(77.64%)d 0.05(0.98%) f 0.00(0.02%)
31. (1.98704) BD (1) N 1- H 42	32. (1.97320) BD (1) N 2-Si 16

(69.20%) 0.8319* N 1 s(25.71%)p 2.88(74.15%)d 0.01(0.13%) f 0.00(0.01%)<br><br>(30.80%) 0.5550* H 42 s(99.87%)p 0.00(0.13%)d 0.00(0.01%)	(81.86%) 0.9048* N 2 s(37.75%)p 1.65(62.13%)d 0.00(0.12%) f 0.00(0.01%)<br><br>(18.14%) 0.4259*Si 16 s(21.92%)p 3.52(77.09%)d 0.04(0.97%) f 0.00(0.02%)
33. (1.97512) BD (1) N 2-Si 29<br><br>(81.65%) 0.9036* N 2 s(37.92%)p 1.63(61.95%)d 0.00(0.12%) f 0.00(0.01%)<br><br>(18.35%) 0.4284*Si 29 s(22.07%)p 3.49(76.96%)d 0.04(0.94%) f 0.00(0.02%)	34. (1.97949) BD (1)Si 3- C 4<br><br>(27.82%) 0.5274*Si 3 s(26.81%)p 2.71(72.61%)d 0.02(0.57%) f 0.00(0.01%)<br><br>(72.18%) 0.8496* C 4 s(29.74%)p 2.35(69.98%)d 0.01(0.27%) f 0.00(0.02%)

Table S 19: Summary of selected NLMOs of **1** all hydrogen atoms are omitted for clarity.

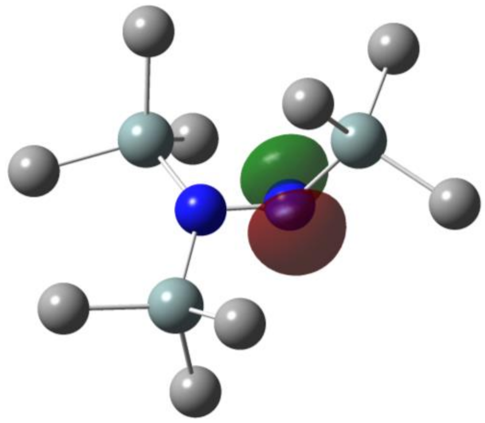	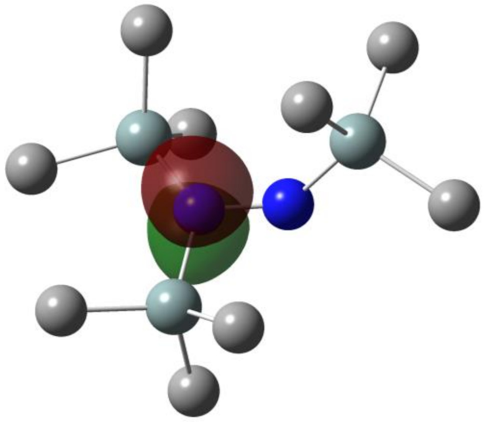
Shortened NLMO Analysis of LP at N1 (threshold > 1.3%)<br><br>27. (2.00000) 94.8633% LP (1) N 1<br><br>94.863% N 1 s(1.07%)p92.16(98.88%)d 0.02(0.02%) f 0.02(0.02%)<br><br>2.456% Si 3 s(0.13%)p99.99(89.33%)d78.13(10.11%) f 3.32(0.43%)	Shortened NLMO Analysis of LP at N2 (threshold > 1.3%)<br><br>28. (2.00000) 92.7903% LP (1) N 2<br><br>92.793% N 2 s(1.72%)p57.03(98.22%)d 0.02(0.04%) f 0.01(0.02%)<br><br>1.985% Si 16 s(0.18%)p99.99(88.79%)d58.55(10.61%) f 2.27(0.41%)

	2.054% Si 29 s(0.12%)p99.99(89.08%)d85.78(10.45%) f 2.85(0.35%)
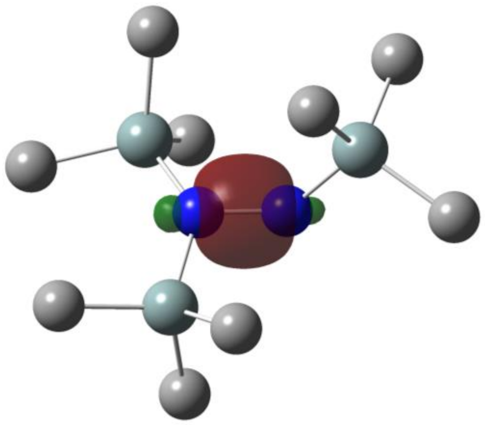	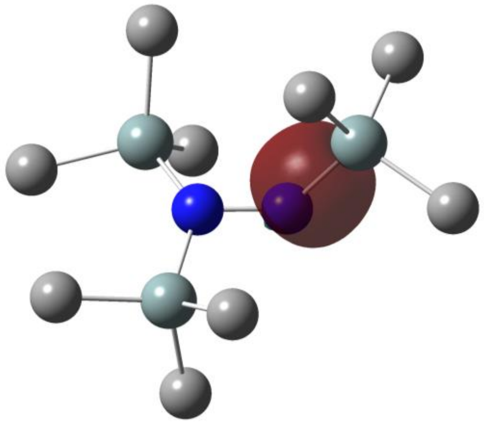
Shortened NLMO Analysis of N1-N2 σ-bond (threshold > 1.3%)<br><br>29. (2.00000) 99.4725% BD (1) N 1- N 2<br><br>50.190% N 1 s(29.38%)p 2.40(70.56%)d 0.00(0.05%) f 0.00(0.02%)<br>49.286% N 2 s(21.99%)p 3.54(77.91%)d 0.00(0.08%) f 0.00(0.01%)	Shortened NLMO Analysis of N1-Si3 σ-bond (threshold > 1.3%)<br><br>30. (2.00000) 98.9787% BD (1) N 1-Si 3<br><br>80.197% N 1 s(43.65%)p 1.29(56.18%)d 0.00(0.16%) f 0.00(0.01%)<br><br>19.045% Si 3 s(31.61%)p 2.14(67.51%)d 0.03(0.85%) f 0.00(0.02%)
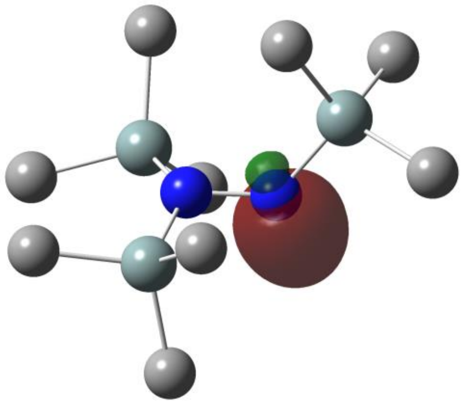	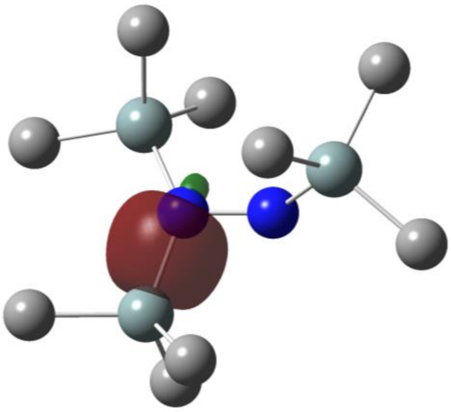
Shortened NLMO Analysis of N1-H42 σ-bond (threshold > 1.3%)<br><br>31. (2.00000) 99.3500% BD (1) N 1- H 42	Shortened NLMO Analysis of N2-Si16 σ-bond (threshold > 1.3%)<br><br>32. (2.00000) 98.6535% BD (1) N 2-Si 16

68.752% N 1 s(25.33%)p 2.94(74.53%)d 0.01(0.13%) f 0.00(0.01%)<br><br>30.607% H 42 s(99.87%)p 0.00(0.13%)d 0.00(0.01%)	80.770% N 2 s(36.44%)p 1.74(63.43%)d 0.00(0.12%) f 0.00(0.01%)<br><br>18.181% Si 16 s(32.76%)p 2.03(66.39%)d 0.03(0.82%) f 0.00(0.02%)
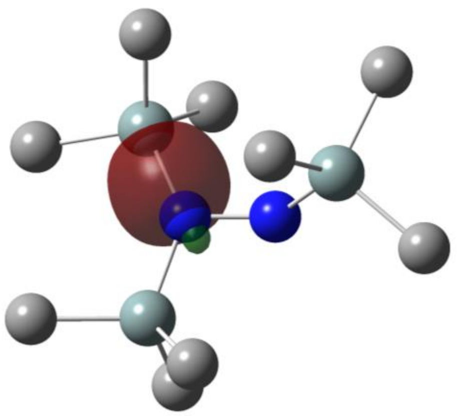	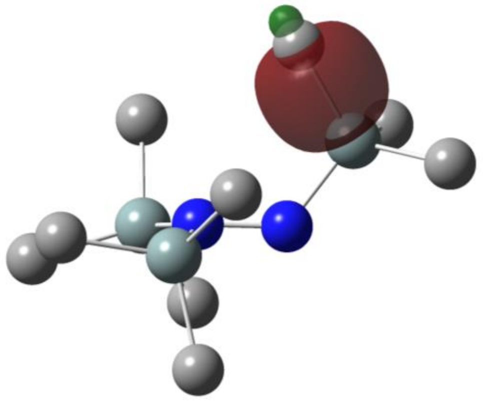
Shortened NLMO Analysis of N2-Si29 σ-bond (threshold > 1.3%)<br><br>33. (2.00000) 98.7495% BD (1) N 2-Si 29<br><br>80.660% N 2 s(36.44%)p 1.74(63.43%)d 0.00(0.12%) f 0.00(0.01%)<br><br>18.395% Si 29 s(33.01%)p 2.00(66.16%)d 0.02(0.80%) f 0.00(0.02%)	Shortened NLMO Analysis of Si3-C4 σ-bond (threshold > 1.3%)<br><br>34. (2.00000) 98.9699% BD (1)Si 3- C 4<br><br>27.849% Si 3 s(36.58%)p 1.72(62.92%)d 0.01(0.49%) 0.00(0.02%)<br>71.456% C 4 s(28.18%)p 2.54(71.52%)d 0.01(0.28%) f 0.00(0.02%)

Table S 20: Summary of individual LMO bond orders greater than 0.002 in magnitude for the NN bond in **1**.

Atom I	Atom J	NLMO	Bond Order	Hybrid Overlap
1	2	27	0.0028439	0.2462495
1	2	28	0.0094739	0.2223293
1	2	29	0.9857161	0.6906727
1	2	30	0.0027163	0.0284976

1	2	31	-0.0027274	-0.1310898
1	2	32	-0.0030034	-0.0651868
1	2	33	-0.0026556	-0.0703621
1	2	36	-0.0023588	-0.6315123
1	2	48	-0.0023156	-0.7265613
1	2	58	-0.0023896	-0.7095127
	sum of NN Bond Order =		0.9853	

Table S 21: Selected second order perturbation theory analysis of Fock matrix in NBO basis, of the nitrogen lone pairs (LP) of **1**.

Donor (L) NBO	Acceptor (NL) NBO	E(2) [kcal/mol]	E(NL)-E(L) [a.u.]	F(L,NL) [a.u.]
27. LP (1) N 1	73. BD*(1) N 2-Si 16	0.82	0.54	0.019
27. LP (1) N 1	74. BD*(1) N 2-Si 29	2.36	0.54	0.032
27. LP (1) N 1	76. BD*(1)Si 3- C 8	9.22	0.53	0.062
27. LP (1) N 1	77. BD*(1)Si 3- C 12	4.88	0.53	0.045
27. LP (1) N 1	89. BD*(1)Si 16- C 25	2.21	0.53	0.031
27. LP (1) N 1	99. BD*(1)Si 29- C 30	1.44	0.53	0.025
28. LP (1) N 2	71. BD*(1) N 1-Si 3	1.7	0.54	0.027
28. LP (1) N 2	72. BD*(1) N 1- H 42	8.59	0.69	0.069
28. LP (1) N 2	87. BD*(1)Si 16- C 17	8.42	0.52	0.059
28. LP (1) N 2	88. BD*(1)Si 16- C 21	3.86	0.53	0.04
28. LP (1) N 2	100. BD*(1)Si 29- C 34	7.24	0.52	0.055
28. LP (1) N 2	101. BD*(1)Si 29- C 38	5.57	0.52	0.048

9.3.1.2 NBO / NLMO Analysis of $1^{\cdot+}$

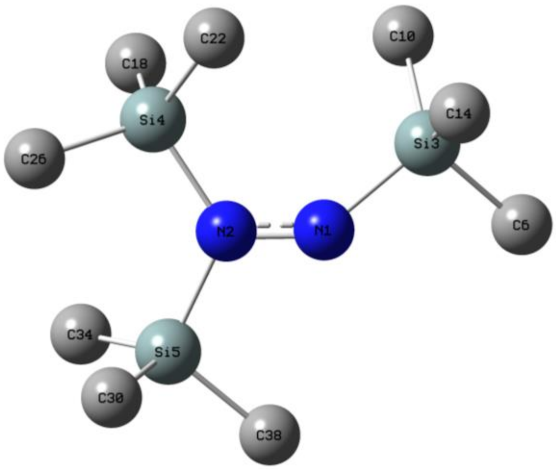

Figure S 22: Numbering of $\mathbf{1}^{\cdot+}$ Hydrogen atoms are omitted for clarity H42 is connected at N1.

Table S 22: Summary of selected NBO analysis of $\mathbf{1}^{\cdot+}$; α-spin.

27. (0.96974) LP (1) N 1<br><br>s(0.01%)p99.99(99.95%)d 1.11(0.01%) f 2.39(0.02%)	28. (0.95766) LP (1) N 2<br><br>s(0.23%)p99.99(99.75%)d 0.02(0.00%) f 0.07(0.01%)
29. (0.99843) BD (1) N 1- N 2<br><br>(50.79%) 0.7127* N 1 s(33.08%)p 2.02(66.83%)d 0.00(0.06%) f 0.00(0.03%)<br><br>(49.21%) 0.7015* N 2 s(29.20%)p 2.42(70.66%)d 0.00(0.12%) f 0.00(0.02%)	30. (0.98750) BD (1) N 1-Si 3<br><br>(83.42%) 0.9133* N 1 s(41.86%)p 1.39(58.05%)d 0.00(0.08%) f 0.00(0.01%)<br><br>(16.58%) 0.4072*Si 3 s(15.48%)p 5.39(83.37%)d 0.07(1.11%) f 0.00(0.04%)
31. (0.99136) BD (1) N 1- H 42<br><br>(71.25%) 0.8441* N 1 s(25.28%)p 2.95(74.59%)d 0.00(0.12%) f 0.00(0.01%)	32. (0.98395) BD (1) N 2-Si 4<br><br>(83.54%) 0.9140* N 2 s(36.65%)p 1.73(63.27%)d 0.00(0.08%) f 0.00(0.01%)

(28.75%) 0.5362* H 42 s(99.88%)p 0.00(0.11%)d 0.00(0.01%)	(16.46%) 0.4057*Si 4 s(16.42%)p 5.02(82.46%)d 0.07(1.09%) f 0.00(0.03%)
33. (0.98088) BD (1) N 2-Si 5<br><br>(83.47%) 0.9136* N 2 s(34.13%)p 1.93(65.79%)d 0.00(0.08%) f 0.00(0.01%)<br><br>(16.53%) 0.4065*Si 5 s(16.16%)p 5.12(82.69%)d 0.07(1.12%) f 0.00(0.03%)	34. (0.98631) BD (1)Si 3- C 6<br><br>(28.79%) 0.5366*Si 3 s(26.60%)p 2.74(72.85%)d 0.02(0.54%) f 0.00(0.01%)<br><br>(71.21%) 0.8438* C 6 s(29.79%)p 2.34(69.85%)d 0.01(0.35%) f 0.00(0.02%)

Table S 23: Summary of selected NBO analysis of **1**$^{\cdot+}$; β-spin. No LP orbital was found !

27. (0.99834) BD (1) N 1- N 2<br><br>(50.77%) 0.7126* N 1 s(33.64%)p 1.97(66.25%)d 0.00(0.08%) f 0.00(0.03%)<br><br>(49.23%) 0.7016* N 2 s(29.87%)p 2.34(69.98%)d 0.00(0.13%) f 0.00(0.02%)	28. (0.98502) BD (2) N 1- N 2<br><br>(52.47%) 0.7244* N 1 s(0.00%)p 1.00(99.89%)d 0.00(0.06%) f 0.00(0.05%)<br><br>(47.53%) 0.6894* N 2 s(0.03%)p99.99(99.85%)d 2.53(0.08%) f 1.13(0.04%)
29. (0.98694) BD (1) N 1-Si 3<br><br>(82.58%) 0.9087* N 1 s(41.49%)p 1.41(58.42%)d 0.00(0.08%) f 0.00(0.01%)<br><br>(17.42%) 0.4174*Si 3 s(15.98%)p 5.19(82.94%)d 0.06(1.03%) f 0.00(0.04%)	30. (0.99103) BD (1) N 1- H 42<br><br>(69.94%) 0.8363* N 1 s(25.03%)p 2.99(74.82%)d 0.01(0.13%) f 0.00(0.01%)<br><br>(30.06%) 0.5483* H 42 s(99.89%)p 0.00(0.10%)d 0.00(0.01%)
31. (0.98298) BD (1) N 2-Si 4<br><br>(82.64%) 0.9091* N 2 s(36.34%)p 1.75(63.57%)d 0.00(0.08%) f 0.00(0.01%)<br><br>(17.36%) 0.4167*Si 4 s(16.96%)p 4.83(82.00%)d 0.06(1.01%) f 0.00(0.03%)	32. (0.97971) BD (1) N 2-Si 5<br><br>(82.55%) 0.9086* N 2 s(33.89%)p 1.95(66.02%)d 0.00(0.09%) f 0.00(0.01%)<br><br>(17.45%) 0.4177*Si 5 s(16.65%)p 4.94(82.28%)d 0.06(1.04%) f 0.00(0.03%)
33. (0.98560) BD (1)Si 3- C 6	

(28.80%) 0.5366*Si 3 s(26.48%)p 2.76(72.97%)d 0.02(0.53%) f 0.00(0.01%)<br><br>(71.20%) 0.8438* C 6 s(29.79%)p 2.34(69.84%)d 0.01(0.35%) f 0.00(0.02%)	

Table S 24: Summary of selected NLMOs of $\mathbf{1}^{\cdot+}$; α-spin, all hydrogen atoms are omitted for clarity.

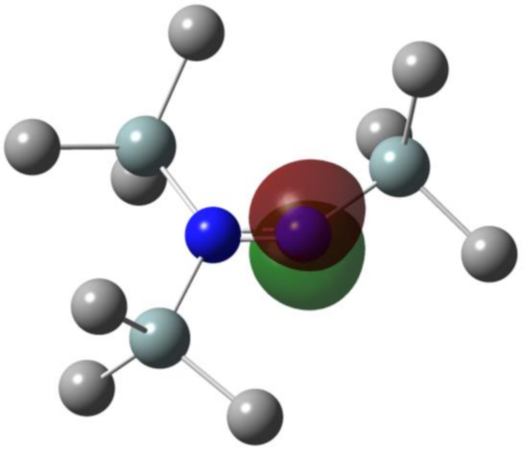	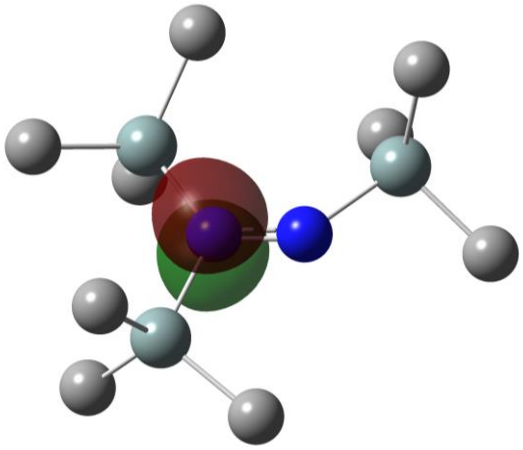
Shortened NLMO Analysis of LP at N1 (threshold > 1.3%)<br><br>27. (1.00000) 96.9341% LP (1) N 1<br><br>96.934% N 1 s(0.01%)p99.99(99.95%)d 1.07(0.01%) f 2.20(0.02%)<br><br>1.457% Si 3 s(0.03%)p99.99(88.16%)d99.99(11.00%) f28.22(0.81%)	Shortened NLMO Analysis of LP at N2 (threshold > 1.3%)<br><br>28. (1.00000) 95.7148% LP (1) N 2<br><br>95.716% N 2 s(0.21%)p99.99(99.77%)d 0.02(0.00%) f 0.07(0.01%)

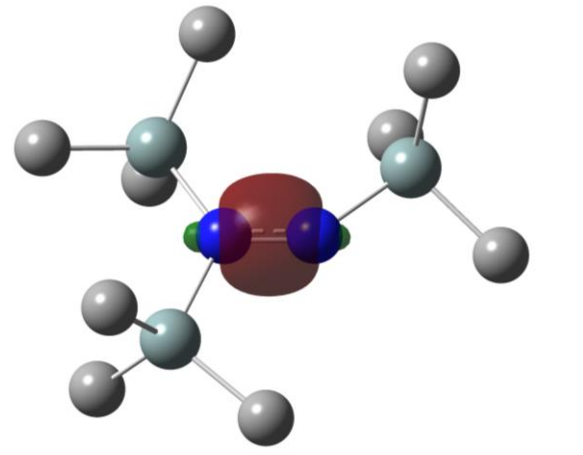	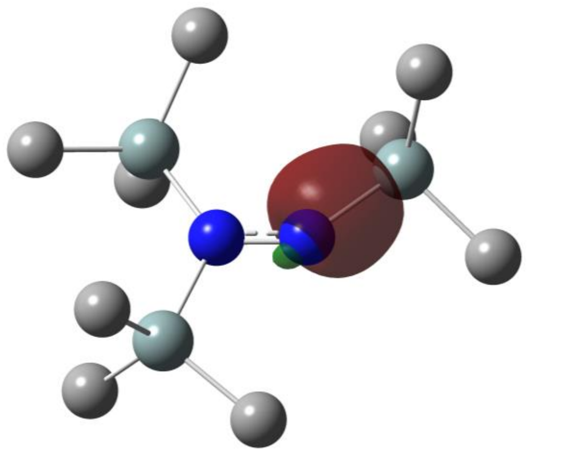
Shortened NLMO Analysis of N1-N2 σ-bond (threshold > 1.3%)<br><br>29. (1.00000) 99.8434% BD (1) N 1- N 2<br><br>50.709% N 1 s(32.96%)p 2.03(66.95%)d 0.00(0.06%) f 0.00(0.03%)<br><br>49.135% N 2 s(29.00%)p 2.44(70.86%)d 0.00(0.12%) f 0.00(0.02%)	Shortened NLMO Analysis of N1-Si3 σ-bond (threshold > 1.3%)<br><br>30. (1.00000) 98.7416% BD (1) N 1-Si 3<br><br>82.370% N 1 s(41.58%)p 1.40(58.33%)d 0.00(0.08%) f 0.00(0.01%)<br><br>16.721% Si 3 s(27.18%)p 2.64(71.84%)d 0.03(0.93%) f 0.00(0.04%)
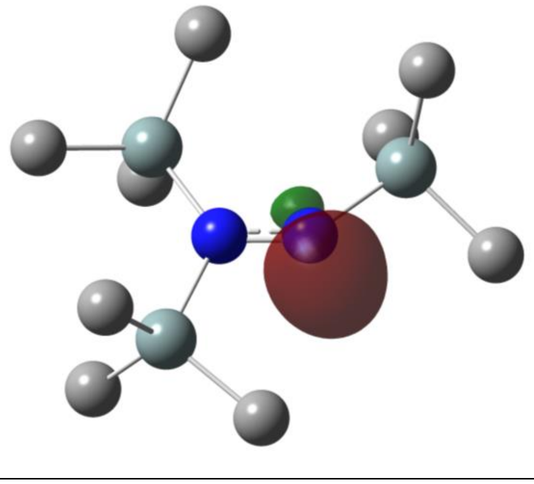	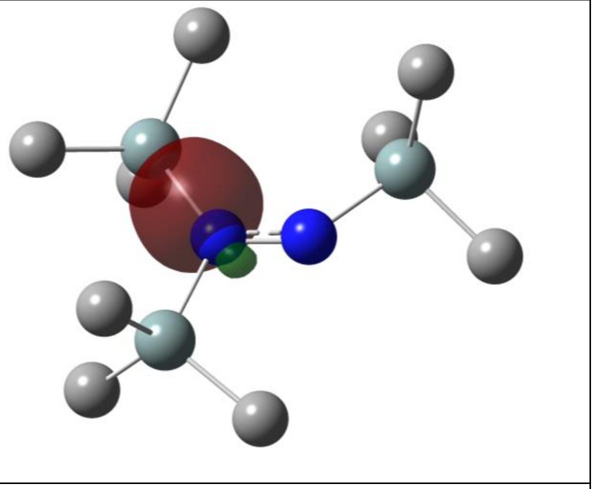
Shortened NLMO Analysis of N1-H42 σ-bond (threshold > 1.3%)<br><br>31. (1.00000) 99.1320% BD (1) N 1- H 42	Shortened NLMO Analysis of N2-Si4 σ-bond (threshold > 1.3%)<br><br>32. (1.00000) 98.3833% BD (1) N 2-Si 4

70.627% N 1 s(24.98%)p 3.00(74.89%)d 0.00(0.12%) f 0.00(0.01%)<br><br>28.506% H 42 s(99.88%)p 0.00(0.11%)d 0.00(0.01%)	82.205% N 2 s(35.89%)p 1.78(64.03%)d 0.00(0.08%) f 0.00(0.01%)<br><br>16.532% Si 4 s(28.41%)p 2.49(70.66%)d 0.03(0.90%) f 0.00(0.03%)
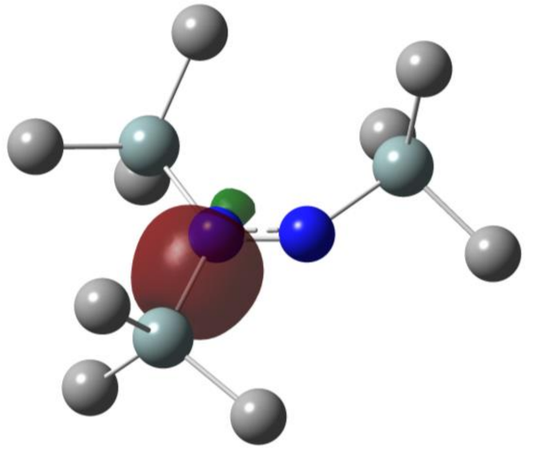	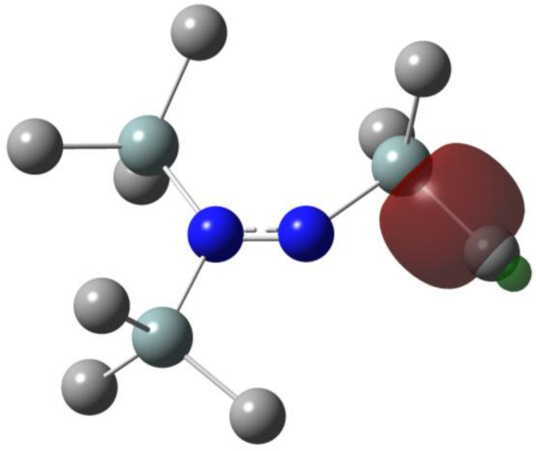
Shortened NLMO Analysis of N2-Si5 σ-bond (threshold > 1.3%)<br><br>33. (1.00000) 98.0789% BD (1) N 2-Si 5<br><br>81.897% N 2 s(33.67%)p 1.97(66.24%)d 0.00(0.08%) f 0.00(0.01%)<br><br>16.549% Si 5 s(28.38%)p 2.49(70.67%)d 0.03(0.92%) f 0.00(0.03%)	Shortened NLMO Analysis of Si3-C6 σ-bond (threshold > 1.3%)<br><br>34. (1.00000) 98.6240% BD (1)Si 3- C 6<br><br>28.675% Si 3 s(35.52%)p 1.80(64.00%)d 0.01(0.46%) f 0.00(0.02%)<br><br>70.246% C 6 s(28.55%)p 2.49(71.07%)d 0.01(0.36%) f 0.00(0.02%)

Table S 25: Summary of selected NLMOs of $\mathbf{1}^{\cdot+}$ β-spin, all hydrogen atoms are omitted for clarity..

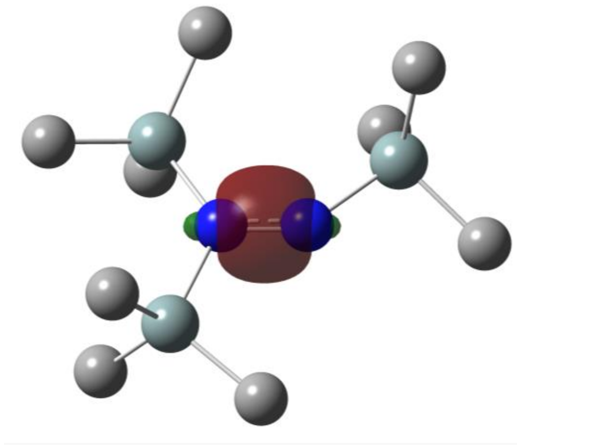	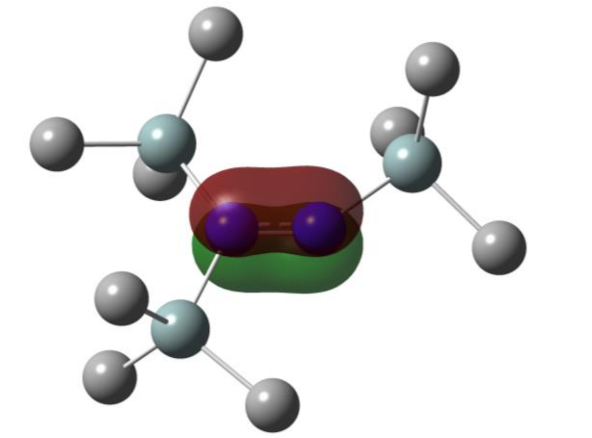
Shortened NLMO Analysis of N1-N2 σ-bond (threshold > 1.3%)<br><br>27. (1.00000) 99.8343% BD (1) N 1- N 2<br><br>50.689% N 1 s(33.35%)p 2.00(66.54%)d 0.00(0.08%) f 0.00(0.03%)<br><br>49.147% N 2 s(29.49%)p 2.39(70.36%)d 0.00(0.13%) f 0.00(0.02%)	Shortened NLMO Analysis of N1-N2 π-bond (threshold > 1.3%)<br><br>28. (1.00000) 98.4965% BD (2) N 1- N 2<br><br>51.715% N 1 s(0.00%)p 1.00(99.89%)d 0.00(0.06%) f 0.00(0.05%)<br><br>46.783% N 2 s(0.04%)p99.99(99.84%)d 2.08(0.08%) f 0.92(0.04%)
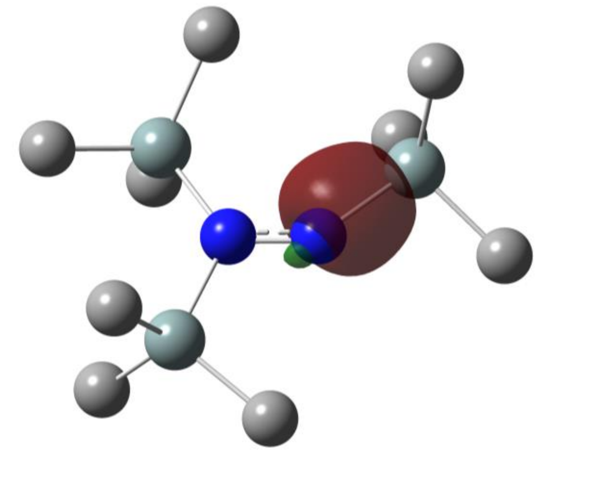	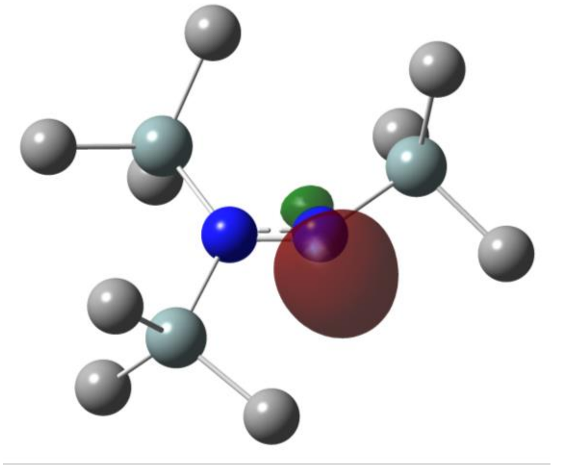
Shortened NLMO Analysis of N1-Si3 σ-bond (threshold > 1.3%)<br><br>29. (1.00000) 98.6842% BD (1) N 1-Si 3	Shortened NLMO Analysis of N1-H42 σ-bond (threshold > 1.3%)<br><br>30. (1.00000) 99.0988% BD (1) N 1- H 42

81.491% N 1 s(41.08%)p 1.43(58.83%)d 0.00(0.08%) f 0.00(0.01%)<br><br>17.554% Si 3 s(27.68%)p 2.58(71.41%)d 0.03(0.86%) f 0.00(0.04%)	69.303% N 1 s(24.59%)p 3.06(75.26%)d 0.01(0.13%) f 0.00(0.01%)<br><br>29.798% H 42 s(99.89%)p 0.00(0.10%)d 0.00(0.01%)
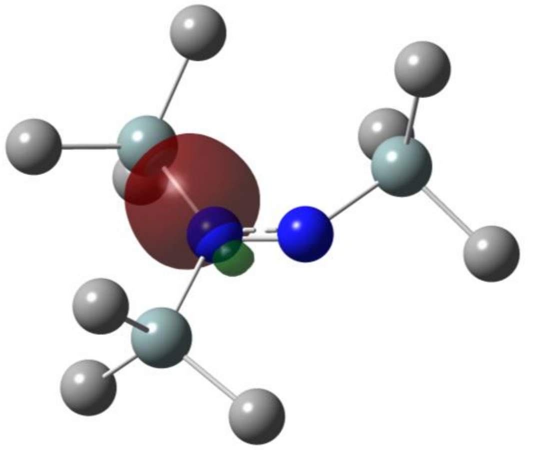	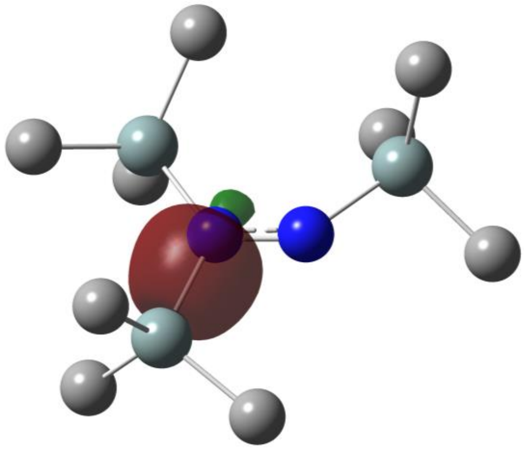
Shortened NLMO Analysis of N2-Si4 σ-bond (threshold > 1.3%)<br><br>31. (1.00000) 98.2842% BD (1) N 2-Si 4<br><br>81.232% N 2 s(35.39%)p 1.82(64.52%)d 0.00(0.08%) f 0.00(0.01%)<br><br>17.435% Si 4 s(29.20%)p 2.39(69.94%)d 0.03(0.83%) f 0.00(0.03%)	Shortened NLMO Analysis of N2-Si5 σ-bond (threshold > 1.3%)<br><br>32. (1.00000) 97.9599% BD (1) N 2-Si 5<br><br>80.896% N 2 s(33.30%)p 2.00(66.61%)d 0.00(0.09%) f 0.00(0.01%)<br><br>17.450% Si 5 s(28.96%)p 2.42(70.15%)d 0.03(0.85%) f 0.00(0.03%)
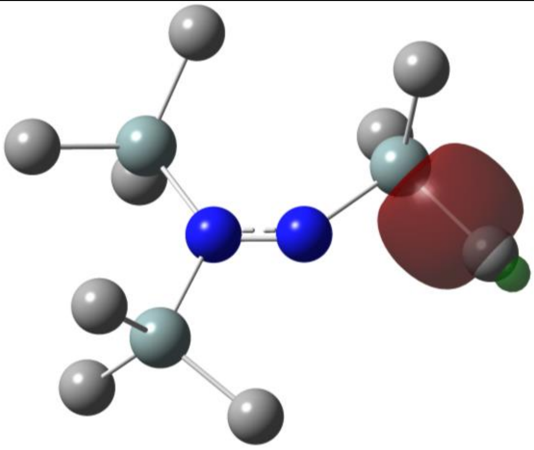	

Shortened NLMO Analysis of Si3-C6 σ-bond (threshold > 1.3%)<br><br>33. (1.00000) 98.5541% BD (1)Si 3- C 6<br><br>28.641% Si 3 s(35.24%)p 1.82(64.28%)d 0.01(0.46%) f 0.00(0.02%)<br><br>70.197% C 6 s(28.56%)p 2.49(71.06%)d 0.01(0.36%) f 0.00(0.02%)	

Table S 26: Summary of individual LMO bond orders greater than 0.002 in magnitude for the NN bond in **1**$^{\cdot+}$.

Atom I	Atom J	NLMO	Bond Order	Hybrid Overlap
1	2	27 (α-spin)	-0.0042187	-0.0627504
1	2	28 (α-spin)	-0.0057865	-0.1068890
1	2	29 (α-spin)	0.4913482	0.7389151
1	2	32 (α-spin)	0.0026983	0.1716471
1	2	33 (α-spin)	0.0028373	0.1515704
1	2	27 (β-spin)	0.4914706	0.7420062
1	2	28 (β-spin)	0.4678331	0.3725124
1	2	31 (β-spin)	0.0028669	0.2109669
1	2	32 (β-spin)	0.0029867	0.1998670
1	2	33 (β-spin)	-0.0023624	-0.5696438
1	2	35 (β-spin)	-0.0033519	-0.3226394
1	2	36 (β-spin)	-0.0041209	-0.3239629

1	2	39 (β-spin)	-0.0028384	-0.3471536
1	2	40 (β-spin)	-0.0032327	-0.3677863
	Sum of NN Bond Order =		1.43613	

Table S 27: Selected second order perturbation theory analysis of Fock matrix in NBO basis, of **1**$^{\cdot+}$.

Donor (L) NBO	Acceptor (NL) NBO	E(2) [kcal/mol]	E(NL)-E(L) [a.u.]	F(L,NL) [a.u.]
27. LP (1) N 1 (α)	76. BD*(1)Si 3- C 10	1.73	0.59	0.04
27. LP (1) N 1 (α)	77. BD*(1)Si 3- C 14	2.81	0.59	0.052
28. LP (1) N 2 (α)	78. BD*(1)Si 4- C 18	2.52	0.59	0.048
28. LP (1) N 2 (α)	79. BD*(1)Si 4- C 22	0.92	0.58	0.029
28. LP (1) N 2 (α)	80. BD*(1)Si 4- C 26	0.43	0.59	0.02
28. LP (1) N 2 (α)	81. BD*(1)Si 5- C 30	1.95	0.58	0.043
28. LP (1) N 2 (α)	82. BD*(1)Si 5- C 34	1.49	0.58	0.037
28. BD (2) N 1- N 2 (β)	76. BD*(1)Si 3- C 10	0.47	0.64	0.022
28. BD (2) N 1- N 2 (β)	77. BD*(1)Si 3- C 14	0.79	0.65	0.029
28. BD (2) N 1- N 2 (β)	78. BD*(1)Si 4- C 18	0.78	0.64	0.028
28. BD (2) N 1- N 2 (β)	81. BD*(1)Si 5- C 30	0.84	0.64	0.029
28. BD (2) N 1- N 2 (b)	82. BD*(1)Si 5- C 34	0.66	0.64	0.026

9.3.1.3 NBO / NLMO Analysis of 2$^+$

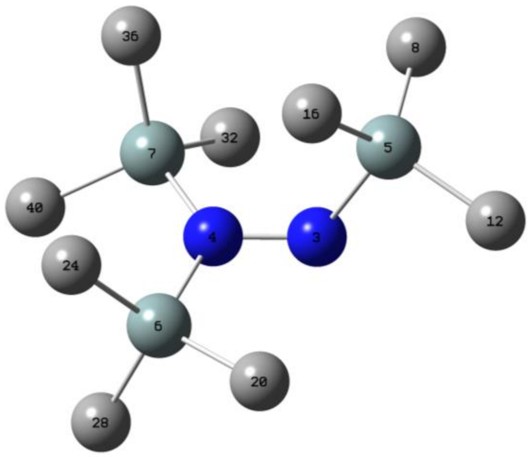

Figure S 23: Numbering of **2**$^+$, Hydrogen atoms are omitted for clarity H1 and H2 are connected at N3

Table S 28: Summary of selected NBO analysis of **2**$^+$.

27. (1.88024) LP (1) N 4<br><br>s(0.04%)p99.99(99.93%)d 0.06(0.00%) f 0.48(0.02%)	28. (1.98935) BD (1) H 1- N 3<br><br>(28.09%) 0.5300* H 1 s(99.88%)p 0.00(0.12%)d 0.00(0.01%)<br><br>(71.91%) 0.8480* N 3 s(21.48%)p 3.65(78.35%)d 0.01(0.16%) f 0.00(0.00%)
29. (1.98969) BD (1) H 2- N 3<br><br>(28.24%) 0.5314* H 2 s(99.88%)p 0.00(0.12%)d 0.00(0.01%)<br><br>(71.76%) 0.8471* N 3 s(21.28%)p 3.69(78.54%)d 0.01(0.18%) f 0.00(0.00%)	30. (1.99462) BD (1) N 3- N 4<br><br>(55.19%) 0.7429* N 3 s(27.56%)p 2.63(72.38%)d 0.00(0.05%) f 0.00(0.01%)<br><br>(44.81%) 0.6694* N 4 s(21.99%)p 3.54(77.93%)d 0.00(0.07%) f 0.00(0.01%)
31. (1.97574) BD (1) N 3-Si 5<br><br>(84.55%) 0.9195* N 3 s(29.85%)p 2.35(70.06%)d 0.00(0.08%) f 0.00(0.00%)<br><br>(15.45%) 0.3930*Si 5 s(13.55%)p 6.29(85.22%)d 0.09(1.18%) f 0.00(0.05%)	32. (1.96832) BD (1) N 4-Si 6<br><br>(82.78%) 0.9098* N 4 s(39.19%)p 1.55(60.72%)d 0.00(0.09%) f 0.00(0.01%)<br><br>(17.22%) 0.4150*Si 6 s(18.59%)p 4.32(80.33%)d 0.06(1.06%) f 0.00(0.03%)
33. (1.96951) BD (1) N 4-Si 7<br><br>(82.62%) 0.9089* N 4 s(38.88%)p 1.57(61.02%)d 0.00(0.09%) f 0.00(0.01%)<br><br>(17.38%) 0.4169*Si 7 s(18.71%)p 4.29(80.21%)d 0.06(1.05%) f 0.00(0.03%)	41. (1.97915) BD (1)Si 7- C 36<br><br>(27.98%) 0.5289*Si 7 s(27.52%)p 2.61(71.87%)d 0.02(0.60%) f 0.00(0.02%)<br><br>(72.02%) 0.8487* C 36 s(30.25%)p 2.30(69.42%)d 0.01(0.31%) f 0.00(0.02%)

Table S 29: Summary of selected NLMOs of **2^+** all hydrogen atoms are omitted for clarity..

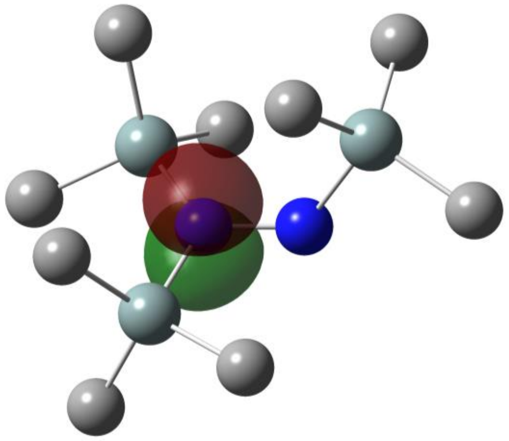	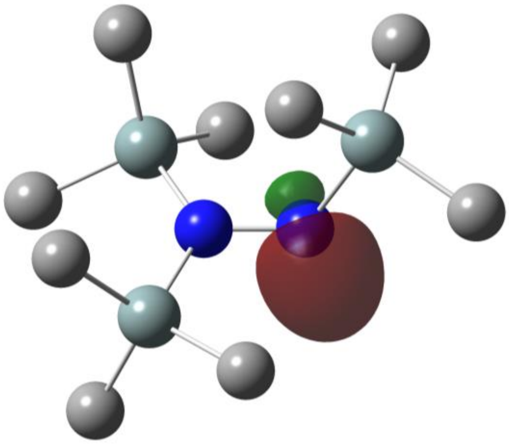
Shortened NLMO Analysis of LP at N4 (threshold > 1.3%)<br><br>27. (2.00000) 93.9505% LP (1) N 4<br><br>93.951% N 4 s(0.04%)p99.99(99.94%)d 0.08(0.00%) f 0.59(0.02%)<br><br>1.527% Si 6 s(0.03%)p99.99(86.82%)d99.99(12.58%) f21.30(0.57%)<br><br>1.574% Si 7 s(0.15%)p99.99(86.99%)d82.37(12.40%) f 3.08(0.46%)	Shortened NLMO Analysis of H1-N3 σ-bond (threshold > 1.3%)<br><br>28. (2.00000) 99.4658% BD (1) H 1- N 3<br><br>27.944% H 1 s(99.88%)p 0.00(0.12%)d 0.00(0.01%)<br><br>71.526% N 3 s(20.85%)p 3.79(78.98%)d 0.01(0.16%) f 0.00(0.00%)
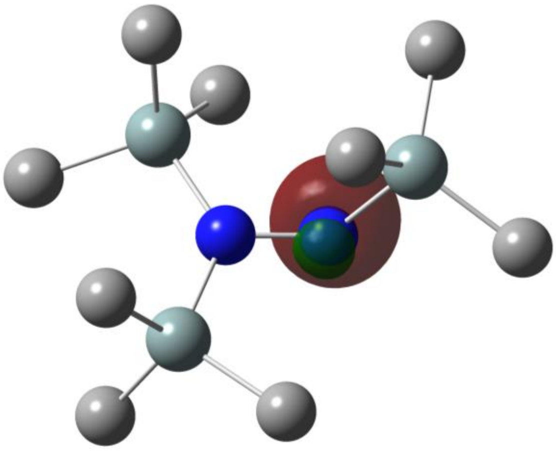	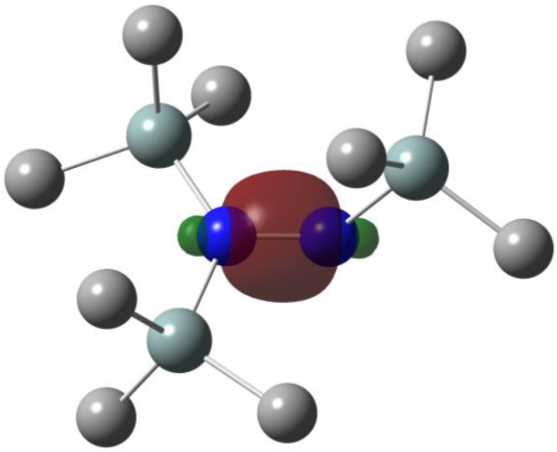
Shortened NLMO Analysis of H2-N3 σ-bond (threshold > 1.3%)	Shortened NLMO Analysis of N3-N4 σ-bond (threshold > 1.3%)

29. (2.00000) 99.4838% BD (1) H 2- N 3<br><br>28.100% H 2 s(99.88%)p 0.00(0.12%)d 0.00(0.01%)<br><br>71.387% N 3 s(20.78%)p 3.80(79.04%)d 0.01(0.18%) f 0.00(0.00%)	30. (2.00000) 99.7310% BD (1) N 3- N 4<br><br>55.039% N 3 s(27.38%)p 2.65(72.56%)d 0.00(0.05%) f 0.00(0.01%)<br><br>44.693% N 4 s(22.02%)p 3.54(77.90%)d 0.00(0.07%) f 0.00(0.01%)
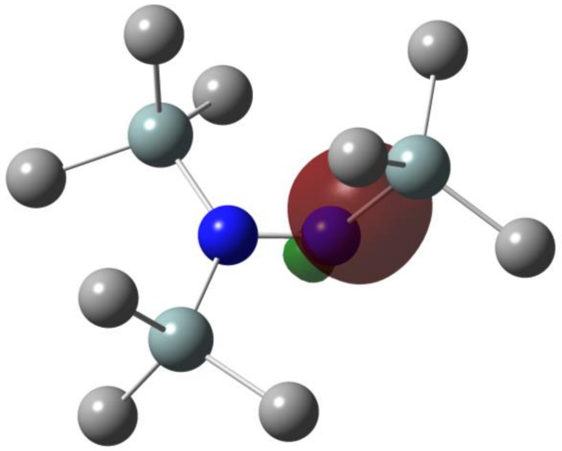	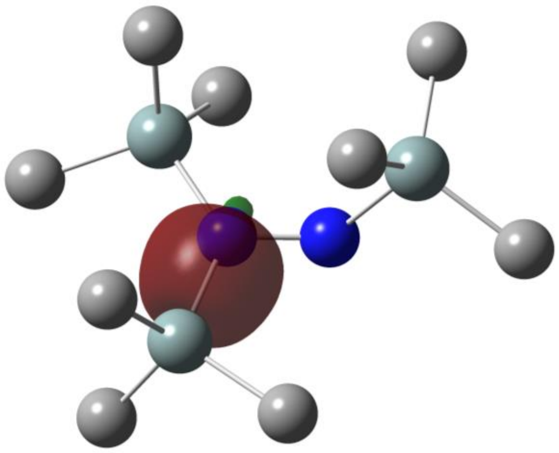
Shortened NLMO Analysis of N3-Si5 σ-bond (threshold > 1.3%)<br><br>31. (2.00000) 98.7777% BD (1) N 3-Si 5<br><br>83.507% N 3 s(28.86%)p 2.46(71.05%)d 0.00(0.09%) f 0.00(0.00%)<br><br>15.710% Si 5 s(26.55%)p 2.73(72.45%)d 0.04(0.95%) f 0.00(0.05%)	Shortened NLMO Analysis of N4-Si6 σ-bond (threshold > 1.3%)<br><br>32. (2.00000) 98.4082% BD (1) N 4-Si 6<br><br>81.484% N 4 s(38.41%)p 1.60(61.49%)d 0.00(0.09%) f 0.00(0.01%)<br><br>17.260% Si 6 s(30.19%)p 2.28(68.90%)d 0.03(0.88%) f 0.00(0.03%)

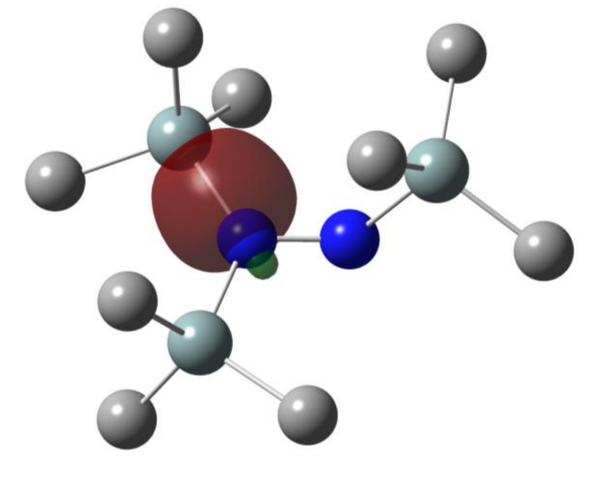	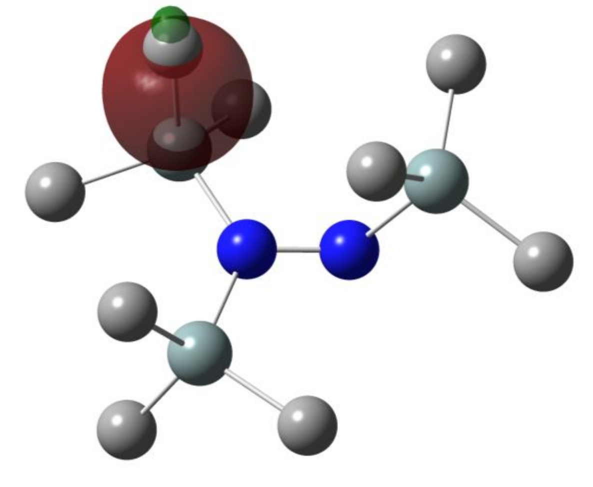
Shortened NLMO Analysis of N4-Si7 σ-bond (threshold > 1.3%)<br><br>33. (2.00000) 98.4662% BD (1) N 4-Si 7<br><br>81.375% N 4 s(37.95%)p 1.63(61.95%)d 0.00(0.09%) f 0.00(0.01%)<br><br>17.411% Si 7 s(29.94%)p 2.31(69.15%)d 0.03(0.88%) f 0.00(0.03%)	Shortened NLMO Analysis of Si7-C36 σ-bond (threshold > 1.3%)<br><br>41. (2.00000) 98.9514% BD (1)Si 7- C 36<br><br>28.061% Si 7 s(38.20%)p 1.60(61.28%)d 0.01(0.50%) f 0.00(0.02%)<br><br>71.273% C 36 s(28.83%)p 2.46(70.83%)d 0.01(0.32%) f 0.00(0.02%)

Table S 30: Summary of individual LMO bond orders greater than 0.002 in magnitude for the NN bond in **2**$^{+}$.

Atom I	Atom J	NLMO	Bond Order	Hybrid Overlap
3	4	27	0.0090677	0.2456945
3	4	29	-0.0021096	-0.0813479
3	4	30	0.8938511	0.6817415
3	4	31	-0.0026793	-0.0375771
3	4	32	0.0035086	0.1190772
3	4	33	0.0037732	0.1235674
3	4	38	-0.0020480	-0.6259214
3	4	42	-0.0033816	-0.6771377
	Sum of NN Bond Order =		0.899982	

Table S 31: Selected second order perturbation theory analysis of Fock matrix in NBO basis, of the lone pair at N4 in the **2**$^{+}$.

Donor (L) NBO	Acceptor (NL) NBO	E(2) [kcal/mol]	E(NL)-E(L) [a.u.]	F(L,NL) [a.u.]
27. LP (1) N 4	70. BD*(1) H 1- N 3	1.27	0.59	0.024
27. LP (1) N 4	71. BD*(1) H 2- N 3	3.63	0.59	0.041
27. LP (1) N 4	73. BD*(1) N 3-Si 5	3.21	0.42	0.033
27. LP (1) N 4	80. BD*(1)Si 6- C 24	4.24	0.54	0.043
27. LP (1) N 4	81. BD*(1)Si 6- C 28	6.34	0.55	0.052
27. LP (1) N 4	82. BD*(1)Si 7- C 32	2.12	0.53	0.03
27. LP (1) N 4	83. BD*(1)Si 7- C 36	7.24	0.54	0.056
27. LP (1) N 4	84. BD*(1)Si 7- C 40	1.79	0.54	0.028

9.3.1.4 NBO / NLMO Analysis of 3^+

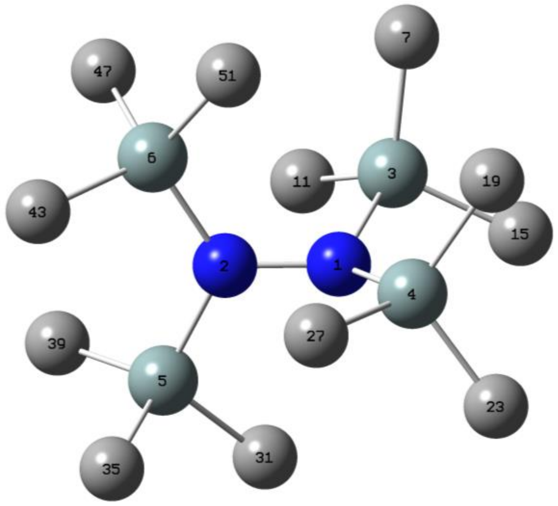

Figure S 24: Numbering of $\mathbf{3}^+$, Hydrogen atoms are omitted for clarity H55 is connected at N1.

Table S 32: Summary of selected NBO analysis of $\mathbf{3}^+$.

35. (1.88174) LP (1) N 2<br><br>s(0.09%)p99.99(99.88%)d 0.04(0.00%) f 0.22(0.02%)	36. (1.99207) BD (1) N 1- N 2<br><br>(53.77%) 0.7333* N 1 s(24.90%)p 3.01(75.03%)d 0.00(0.06%) f 0.00(0.01%)<br><br>(46.23%) 0.6799* N 2 s(23.60%)p 3.24(76.35%)d 0.00(0.04%) f 0.00(0.01%)
37. (1.96844) BD (1) N 1-Si 3<br><br>(84.88%) 0.9213* N 1 s(28.31%)p 2.53(71.59%)d 0.00(0.11%) f 0.00(0.00%)<br><br>(15.12%) 0.3888*Si 3 s(15.56%)p 5.35(83.24%)d 0.08(1.17%) f 0.00(0.03%)	38. (1.96785) BD (1) N 1-Si 4<br><br>(84.88%) 0.9213* N 1 s(27.99%)p 2.57(71.91%)d 0.00(0.10%) f 0.00(0.00%)<br><br>(15.12%) 0.3889*Si 4 s(15.44%)p 5.40(83.36%)d 0.08(1.16%) f 0.00(0.04%)
39. (1.97639) BD (1) N 1- H 55	40. (1.96641) BD (1) N 2-Si 5

(73.66%) 0.8582* N 1 s(19.01%)p 4.25(80.79%)d 0.01(0.19%) f 0.00(0.00%)<br><br>(26.34%) 0.5132* H 55 s(99.89%)p 0.00(0.10%)d 0.00(0.01%)	(82.84%) 0.9102* N 2 s(37.13%)p 1.69(62.76%)d 0.00(0.10%) f 0.00(0.01%)<br><br>(17.16%) 0.4143*Si 5 s(19.59%)p 4.05(79.25%)d 0.06(1.13%) f 0.00(0.02%)
41. (1.96976) BD (1) N 2-Si 6<br><br>(83.01%) 0.9111* N 2 s(39.28%)p 1.54(60.63%)d 0.00(0.09%) f 0.00(0.01%)<br><br>(16.99%) 0.4121*Si 6 s(19.61%)p 4.04(79.21%)d 0.06(1.16%) f 0.00(0.02%)	47. (1.97700) BD (1)Si 4- C 27<br><br>(28.48%) 0.5336*Si 4 s(29.12%)p 2.42(70.35%)d 0.02(0.53%) f 0.00(0.01%)<br><br>(71.52%) 0.8457* C 27 s(29.94%)p 2.32(69.59%)d 0.01(0.45%) f 0.00(0.02%)

Table S 33: Summary of selected NLMOs of **3**⁺ all hydrogen atoms are omitted for clarity.

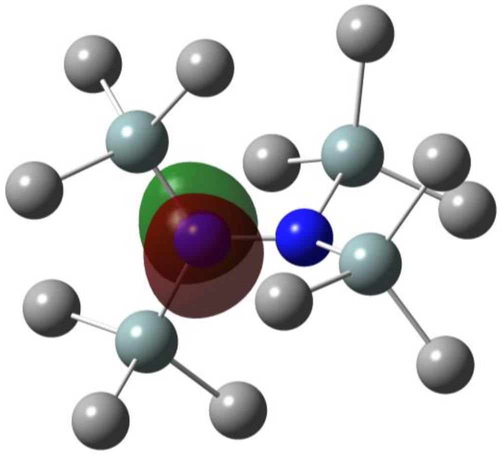	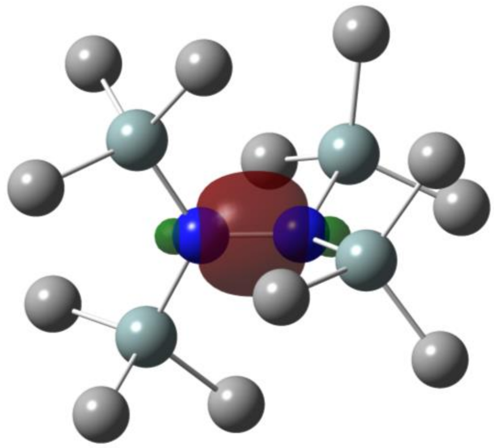
Shortened NLMO Analysis of LP at N2 (threshold > 1.3%)<br><br>35. (2.00000) 94.0122% LP (1) N 2<br><br>94.013% N 2 s(0.08%)p99.99(99.89%)d 0.04(0.00%) f 0.24(0.02%)<br><br>1.673% Si 5 s(0.04%)p99.99(87.73%)d99.99(11.69%) f14.59(0.54%)	Shortened NLMO Analysis of N1-N2 σ-bond (threshold > 1.3%)<br><br>36. (2.00000) 99.6034% BD (1) N 1- N 2<br><br>53.559% N 1 s(24.44%)p 3.09(75.49%)d 0.00(0.06%) f 0.00(0.01%)<br><br>46.047% N 2 s(23.37%)p 3.28(76.58%)d 0.00(0.04%) f 0.00(0.01%)

1.653% Si 6 s(0.01%)p99.99(87.32%)d99.99(12.18%) f34.75(0.48%)	
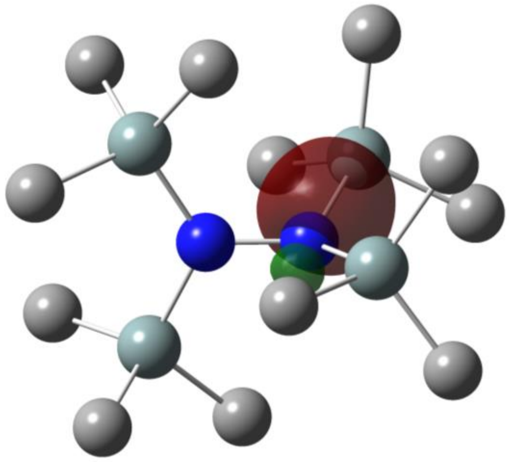	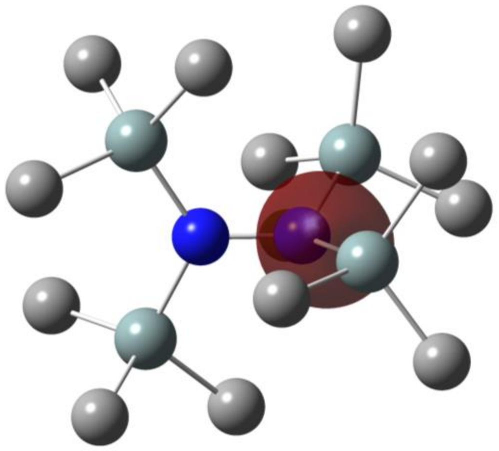
Shortened NLMO Analysis of N1-Si3 σ-bond (threshold > 1.3%)<br><br>37. (2.00000) 98.4122% BD (1) N 1-Si 3<br><br>83.546% N 1 s(27.05%)p 2.69(72.84%)d 0.00(0.11%) f 0.00(0.00%)<br><br>15.326% Si 3 s(29.42%)p 2.37(69.63%)d 0.03(0.93%) f 0.00(0.03%)	Shortened NLMO Analysis of N1-Si4 σ-bond (threshold > 1.3%)<br><br>38. (2.00000) 98.3813% BD (1) N 1-Si 4<br><br>83.507% N 1 s(26.71%)p 2.74(73.19%)d 0.00(0.10%) f 0.00(0.00%)<br><br>15.340% Si 4 s(29.44%)p 2.36(69.61%)d 0.03(0.91%) f 0.00(0.04%)
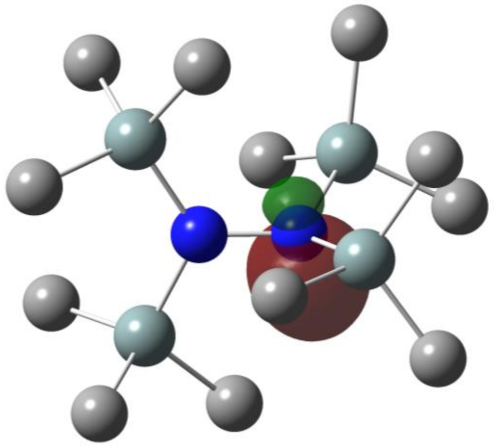	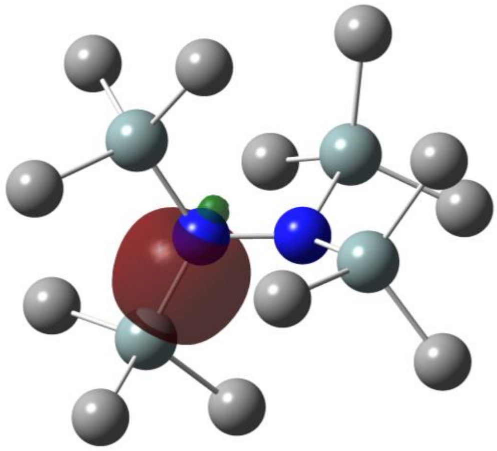
Shortened NLMO Analysis of N1-H55 σ-bond (threshold > 1.3%)	Shortened NLMO Analysis of N2-Si5 σ-bond (threshold > 1.3%)

<table>
<tr><td>39. (2.00000) 98.8156% BD (1) N 1- H 55

72.778% N 1 s(17.75%)p 4.62(82.05%)d 0.01(0.20%) f 0.00(0.00%)

26.058% H 55 s(99.89%)p 0.00(0.10%)d 0.00(0.01%)</td><td>40. (2.00000) 98.3122% BD (1) N 2-Si 5

81.448% N 2 s(36.23%)p 1.76(63.66%)d 0.00(0.10%) f 0.00(0.01%)

17.240% Si 5 s(32.10%)p 2.09(66.95%)d 0.03(0.92%) f 0.00(0.03%)</td></tr>
<tr><td>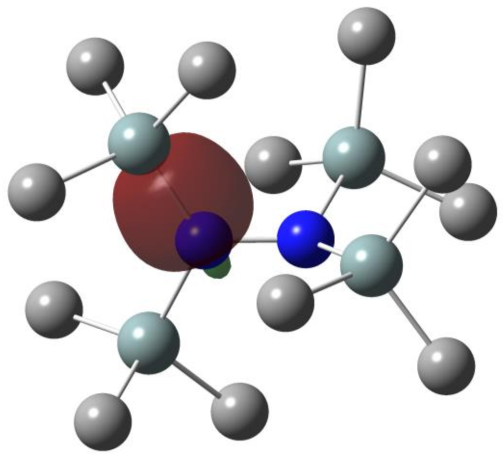</td><td>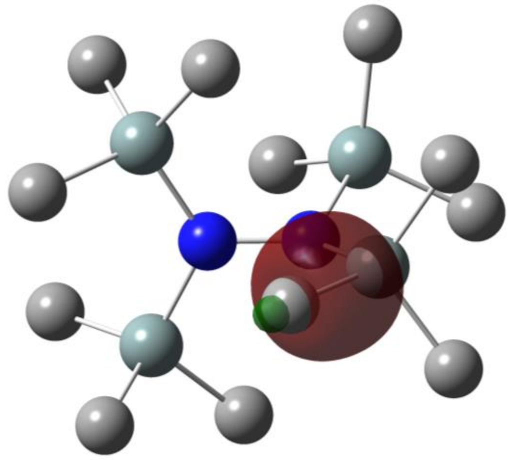</td></tr>
<tr><td>Shortened NLMO Analysis of N2-Si6 σ-bond (threshold > 1.3%)

41. (2.00000) 98.4742% BD (1) N 2-Si 6

81.765% N 2 s(37.99%)p 1.63(61.91%)d 0.00(0.09%) f 0.00(0.01%)

17.067% Si 6 s(31.78%)p 2.12(67.24%)d 0.03(0.95%) f 0.00(0.02%)</td><td>Shortened NLMO Analysis of Si4-C27 σ-bond (threshold > 1.3%)

47. (2.00000) 98.8433% BD (1)Si 4- C 27

28.534% Si 4 s(39.92%)p 1.49(59.63%)d 0.01(0.44%) f 0.00(0.01%)

70.715% C 27 s(28.56%)p 2.48(70.96%)d 0.02(0.46%) f 0.00(0.02%)</td></tr>
</table>

Table S 34: Summary of individual LMO bond orders greater than 0.002 in magnitude for the NN bond in **3**$^{+}$.

Atom I	Atom J	NLMO	Bond Order	Hybrid Overlap
1	2	35	0.0049173	0.2783937
1	2	36	0.9209392	0.6694015
1	2	37	-0.0029239	-0.0481298
1	2	38	-0.0030511	-0.0662175
1	2	39	-0.0023052	-0.0528606
1	2	40	0.0029409	0.0882527
1	2	41	0.0032016	0.1111315
1	2	44	-0.0030807	-0.6435852
1	2	50	-0.0021004	-0.6040764
1	2	51	-0.0041714	-0.7225036
	Sum of NN Bond Order =		0.914366	

Table S 35: Selected second order perturbation theory analysis of Fock matrix in NBO basis, of the **3**$^{+}$.

Donor (L) NBO	Acceptor (NL) NBO	E(2) [kcal/mol]	E(NL)-E(L) [a.u.]	F(L,NL) [a.u.]
35. LP (1) N 2	91. BD*(1) N 1-Si 3	2.32	0.43	0.028
35. LP (1) N 2	92. BD*(1) N 1-Si 4	2.61	0.43	0.03
35. LP (1) N 2	98. BD*(1)Si 3- C 15	0.62	0.51	0.016
35. LP (1) N 2	103. BD*(1)Si 5- C 35	6.39	0.53	0.052
35. LP (1) N 2	104. BD*(1)Si 5- C 39	4.1	0.53	0.042
35. LP (1) N 2	105. BD*(1)Si 6- C 43	2.17	0.54	0.031
35. LP (1) N 2	106. BD*(1)Si 6- C 47	7.19	0.54	0.055
35. LP (1) N 2	107. BD*(1)Si 6- C 51	1.49	0.53	0.025
39. BD (1) N 1- H 55	91. BD*(1) N 1-Si 3	1.38	0.83	0.03

39. BD (1) N 1- H 55	92. BD*(1) N 1-Si 4	1.18	0.83	0.028
39. BD (1) N 1- H 55	95. BD*(1) N 2-Si 6	3.27	0.92	0.049
39. BD (1) N 1- H 55	96. BD*(1)Si 3- C 7	1.06	0.91	0.028
39. BD (1) N 1- H 55	127. BD*(1) C 31- H 33	0.55	1.07	0.022

9.3.1.5 NBO / NLMO Analysis of 5^+

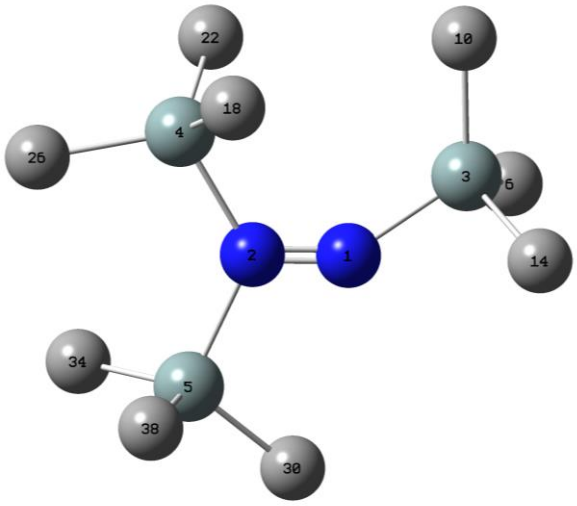

Figure S 25: Numbering of **5⁺**, Hydrogen atoms are omitted for clarity.

Table S 36: Summary of selected NBO analysis of **5⁺**.

27. (1.89915) LP (1) N 1<br><br>s(25.96%)p 2.84(73.86%)d 0.01(0.16%) f 0.00(0.01%)	28. (1.99662) BD (1) N 1- N 2<br><br>(48.28%) 0.6948* N 1 s(37.03%)p 1.69(62.76%)d 0.00(0.17%) f 0.00(0.04%)<br><br>(51.72%) 0.7192* N 2 s(34.58%)p 1.89(65.20%)d 0.01(0.19%)f 0.00(0.04%)
29. (1.98376) BD (2) N 1- N 2<br><br>(41.55%) 0.6446* N 1 s(0.00%)p 1.00(99.73%)d 0.00(0.23%) f 0.00(0.04%)<br><br>(58.45%) 0.7645* N 2 s(0.01%)p 1.00(99.84%)d 0.00(0.12%) f 0.00(0.03%)	30. (1.96222) BD (1) N 1-Si 3<br><br>(81.55%) 0.9031* N 1 s(37.26%)p 1.68(62.61%)d 0.00(0.12%) f 0.00(0.02%)<br><br>(18.45%) 0.4295*Si 3 s(16.54%)p 4.98(82.38%)d 0.06(1.04%) f 0.00(0.04%)
31. (1.97035) BD (1) N 2-Si 4	32. (1.95743) BD (1) N 2-Si 5

(82.98%) 0.9110* N 2 s(33.50%)p 1.98(66.42%)d 0.00(0.07%) f 0.00(0.01%)<br><br>(17.02%) 0.4125*Si 4 s(14.94%)p 5.62(84.04%)d 0.07(0.98%) f 0.00(0.03%)	(83.59%) 0.9143* N 2 s(32.24%)p 2.10(67.69%)d 0.00(0.07%) f 0.00(0.01%)<br><br>(16.41%) 0.4051*Si 5 s(14.55%)p 5.80(84.34%)d 0.07(1.07%) f 0.00(0.03%)

Table S 37: Summary of selected NLMOs of **5**$^+$ all hydrogen atoms are omitted for clarity.

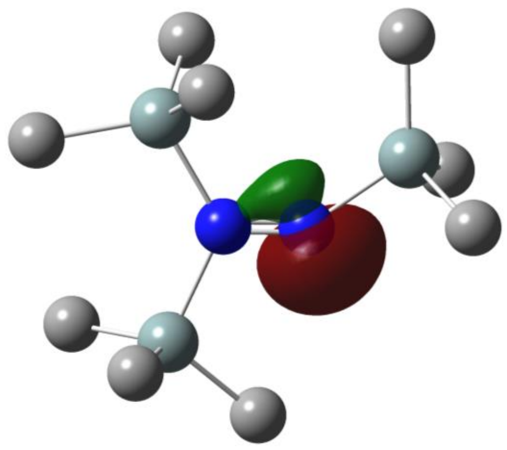	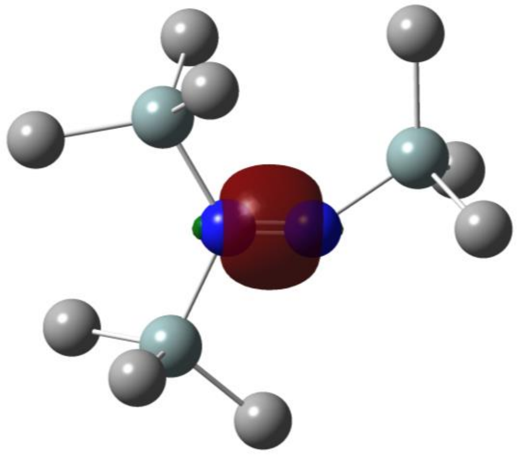
Shortened NLMO Analysis of LP at N1 (threshold > 1.3%)<br><br>27. (2.00000) 94.8837% LP (1) N 1<br><br>94.890% N 1 s(25.54%)p 2.91(74.29%)d 0.01(0.16%) f 0.00(0.01%)<br><br>1.617% Si 3 s(0.29%)p99.99(91.25%)d27.85(7.98%) f 1.70(0.49%)<br><br>1.528% Si 4 s(34.69%)p 1.85(64.26%)d 0.02(0.85%) f 0.01(0.19%)	Shortened NLMO Analysis of N1-N2 σ-bond (threshold > 1.3%)<br><br>28. (2.00000) 99.8310% BD (1) N 1- N 2<br><br>48.198% N 1 s(36.72%)p 1.72(63.07%)d 0.00(0.17%) f 0.00(0.04%)<br><br>51.636% N 2 s(33.93%)p 1.94(65.85%)d 0.01(0.19%) f 0.00(0.04%)

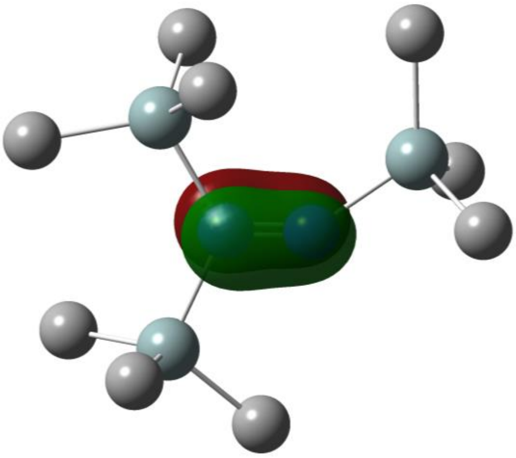<br>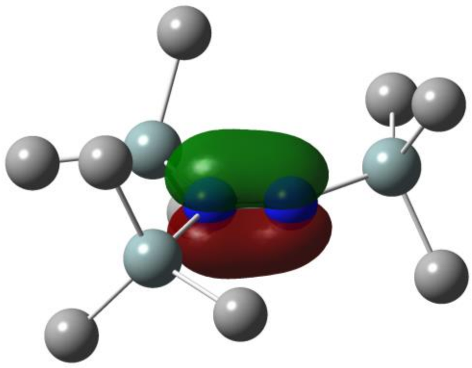	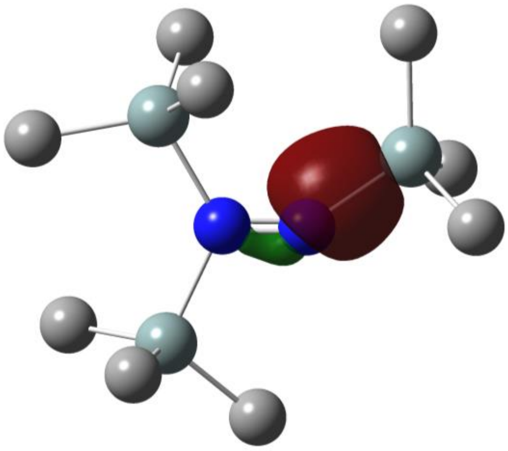
Shortened NLMO Analysis of N1-SN2 π-bond (threshold > 1.3%)<br><br>29. (2.00000) 99.1864% BD (2) N 1- N 2<br><br>41.184% N 1 s(0.00%)p 1.00(99.73%)d 0.00(0.23%) f 0.00(0.04%)<br><br>58.002% N 2 s(0.01%)p 1.00(99.84%)d 0.00(0.12%) f 0.00(0.03	Shortened NLMO Analysis of N1-Si3 σ-bond (threshold > 1.3%)<br><br>30. (2.00000) 98.0872% BD (1) N 1-Si 3<br><br>80.020% N 1 s(37.91%)p 1.63(61.95%)d 0.00(0.12%) f 0.00(0.02%)<br><br>18.422% Si 3 s(27.70%)p 2.58(71.36%)d 0.03(0.89%) f 0.00(0.04%)

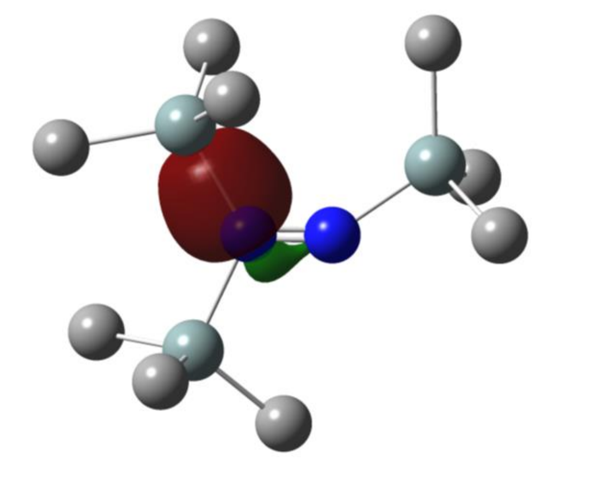	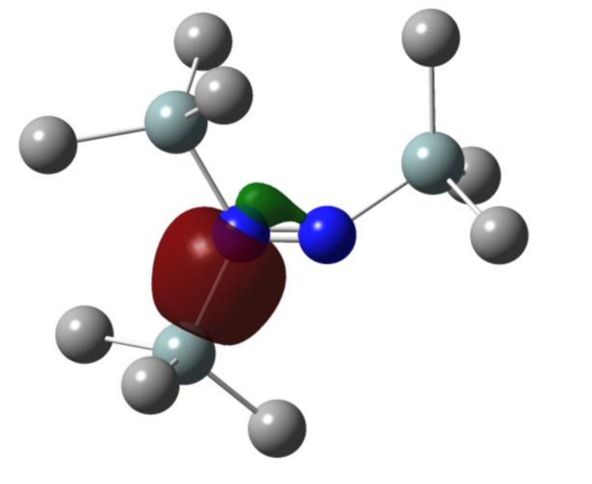
Shortened NLMO Analysis of N2-Si4 σ-bond (threshold > 1.3%)<br><br>31. (2.00000) 98.5069% BD (1) N 2-Si 4<br><br>81.746% N 2 s(33.06%)p 2.02(66.86%)d 0.00(0.07%) f 0.00(0.01%)<br><br>17.159% Si 4 s(27.22%)p 2.64(71.93%)d 0.03(0.81%) f 0.00(0.04%)	Shortened NLMO Analysis of N2-Si5 σ-bond (threshold > 1.3%)<br><br>32. (2.00000) 97.8492% BD (1) N 2-Si 5<br><br>81.787% N 2 s(31.84%)p 2.14(68.08%)d 0.00(0.07%) f 0.00(0.01%)<br><br>16.436% Si 5 s(26.55%)p 2.73(72.53%)d 0.03(0.89%) f 0.00(0.04%)

Table S 38: Summary of individual LMO bond orders greater than 0.002 in magnitude for the NN bond in **5**$^{+}$.

Atom I	Atom J	NLMO	Bond Order	Hybrid Overlap
1	2	27	0.0121960	0.4623529
1	2	28	0.9639609	0.7789600
1	2	29	0.8236847	0.4236410
1	2	30	0.0065884	0.3235033
1	2	31	0.0060599	0.0569015
1	2	32	0.0091003	0.1535768
1	2	33	-0.0117506	-0.4156810
1	2	35	-0.0101176	-0.4260082
1	2	36	-0.0039695	-0.2383439
1	2	37	-0.0037860	-0.2215925
1	2	40	-0.0038560	-0.3947066
1	2	41	-0.0063577	-0.3320277
1	2	43	-0.0027971	-0.3248954
1	2	49	-0.0024376	-0.3258328
	sum of NN bond order =		1.776518	

9.3.2 QT-AIM analysis

9.3.2.1 QT-AIM and WBI analysis of 1

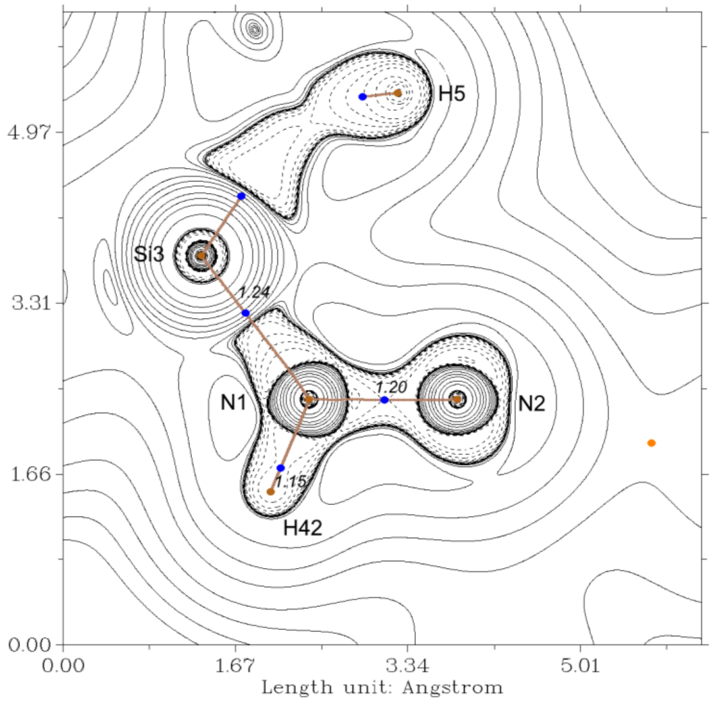

Figure S 26: Contour plot of the Laplacian of the electron density $\nabla^2 r$ of starting material **1** in the Si3-N1-N2 plane. Dashed lines indicate negative (local charge concentration), solid lines indicate positive values (local charge depletion). The Laplacian plot is overlaid with the molecular graph from QT-AIM analysis and Wiberg bond indices (italic small numbers). Brown lines indicate bonding paths, blue dots correspond to bond critical points. Density from m06/def2tzvp calculation.

9.3.2.2 QT-AIM and WBI analysis of 1$^{\cdot+}$

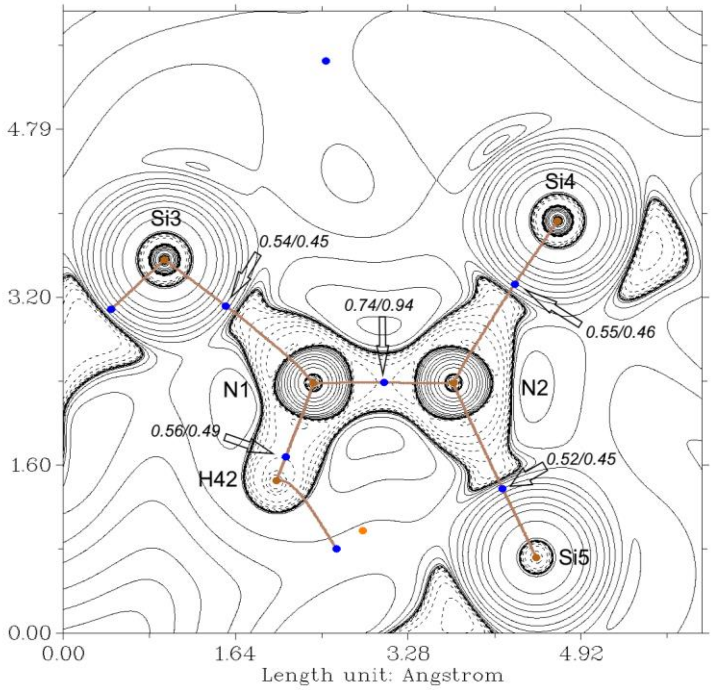

Figure S 27: Contour plot of the Laplacian of the electron density $\nabla^2 r$ of the hydrazinyl radical cation **1**$^{\cdot+}$ in the Si3-N1-N2 plane. Dashed lines indicate negative (local charge concentration), solid lines indicate positive values (local charge depletion). The Laplacian plot is overlaid with the molecular graph from QT-AIM analysis and Wiberg bond indices (italic small numbers; values of the bond order matrix for alpha and beta electrons are reported). Brown lines indicate bonding paths, blue dots correspond to bond critical points. Density from m06/def2tzvp calculation.

9.3.2.3 QT-AIM and WBI analysis of 2⁺

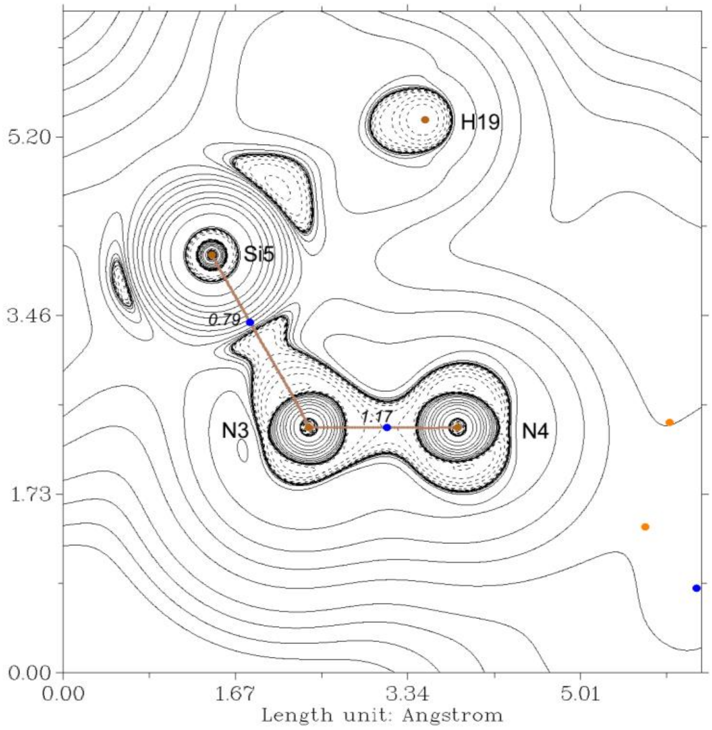

Figure S 28: Contour plot of the Laplacian of the electron density $\nabla^2 r$ of cation **2⁺** in the Si5-N3-N4 plane. Dashed lines indicate negative (local charge concentration), solid lines indicate positive values (local charge depletion). The Laplacian plot is overlaid with the molecular graph from QT-AIM analysis and Wiberg bond indices (italic small numbers). Brown lines indicate bonding paths, blue dots correspond to bond critical points. Density from m06/def2tzvp calculation.

9.3.2.4 QT-AIM and WBI analysis of 3^+

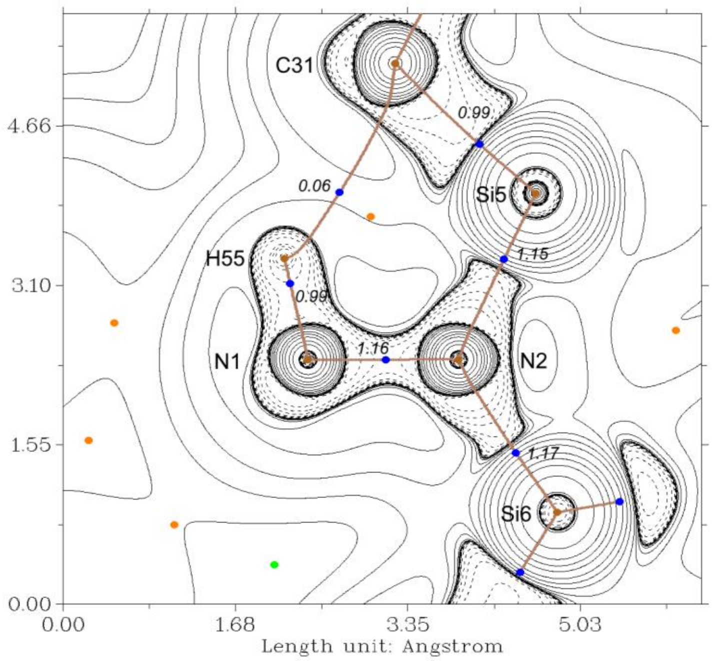

Figure S 29: Contour plot of the Laplacian of the electron density $\nabla^2 r$ of cation $\mathbf{3^+}$ in the H55-N1-N2 plane. Dashed lines indicate negative (local charge concentration), solid lines indicate positive values (local charge depletion). The Laplacian plot is overlaid with the molecular graph from QT-AIM analysis and Wiberg bond indices (italic small numbers). Brown lines indicate bonding paths, blue dots correspond to bond critical points. Density from m06/def2tzvp calculation.

9.3.2.5 QT-AIM and WBI analysis of $(Me_3Si)_2NN(SiMe_3)^+$ (5^+)

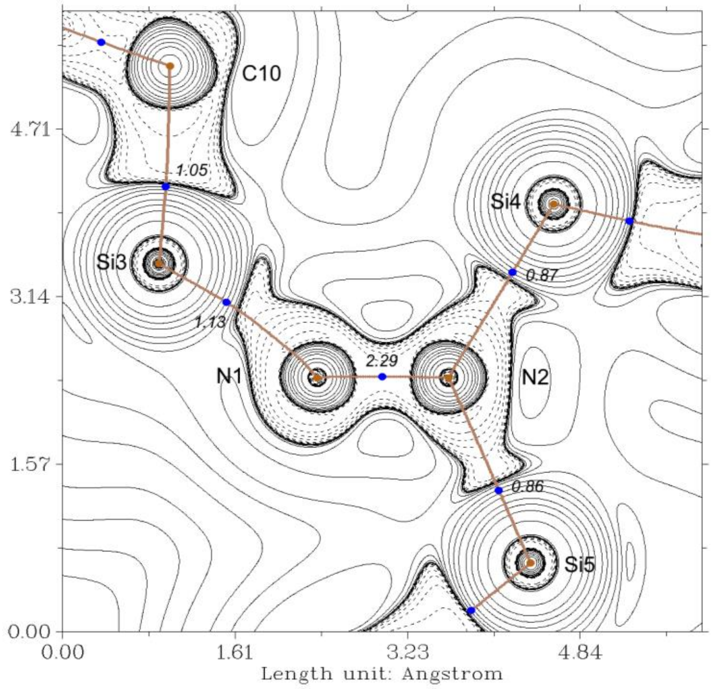

Figure S 30: Contour plot of the Laplacian of the electron density $\nabla^2 r$ of diazenium cation $\mathbf{5}^+$ in the Si3-N1-N2 plane. Dashed lines indicate negative (local charge concentration), solid lines indicate positive values (local charge depletion). The Laplacian plot is overlaid with the molecular graph from QT-AIM analysis and Wiberg bond indices (italic small numbers). Brown lines indicate bonding paths, blue dots correspond to bond critical points. Density from m06/def2tzvp calculation.

9.3.3 Electron Localisation Function (ELF) analysis

9.3.3.1 ELF analysis of 1

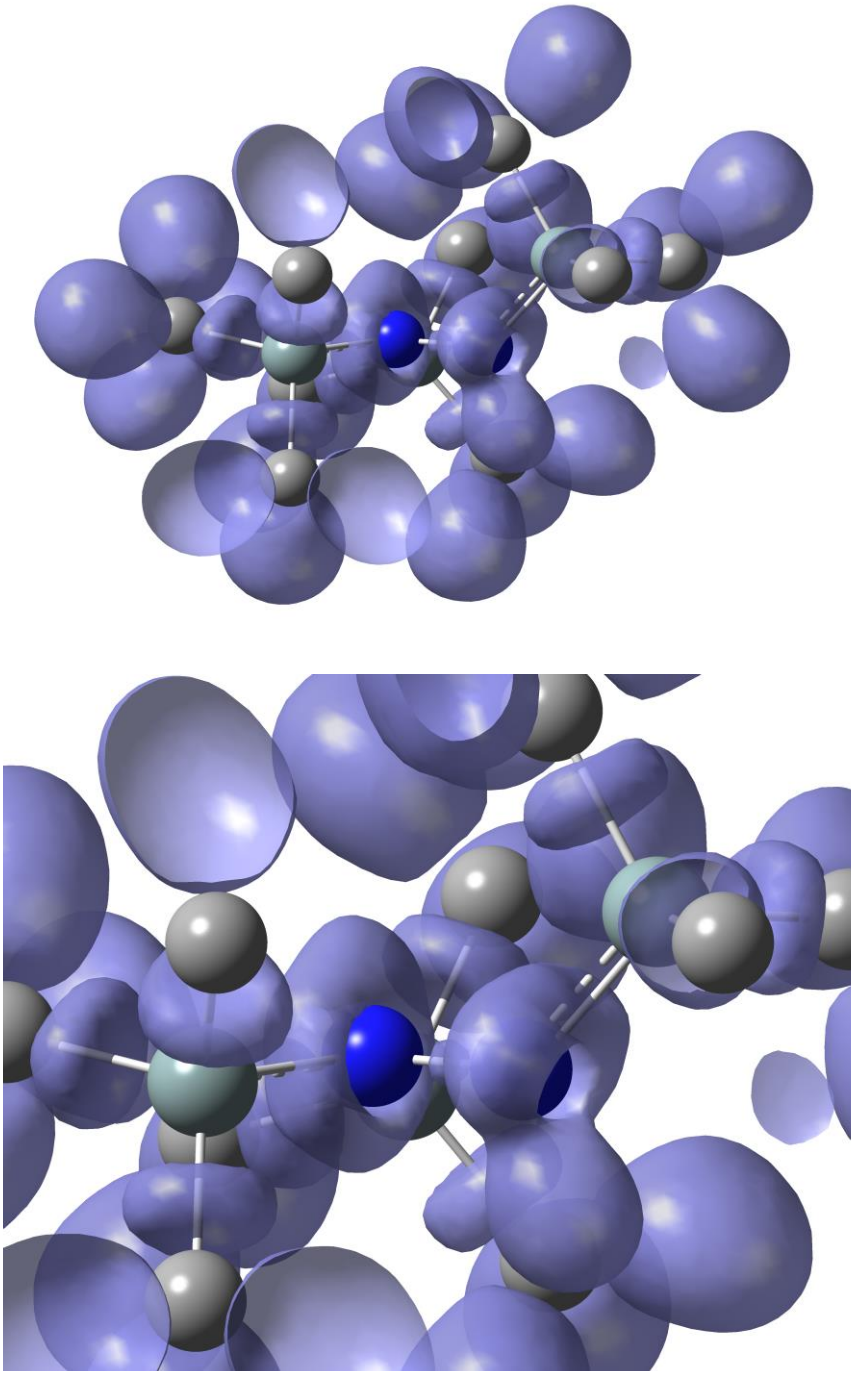

Figure S 31: 3D-ELF plot of **1** (whole molecule top, zoomed view to the NN unit bottom) hydrogen atoms were omitted for clarity, and z-clip function was used for a better understanding.

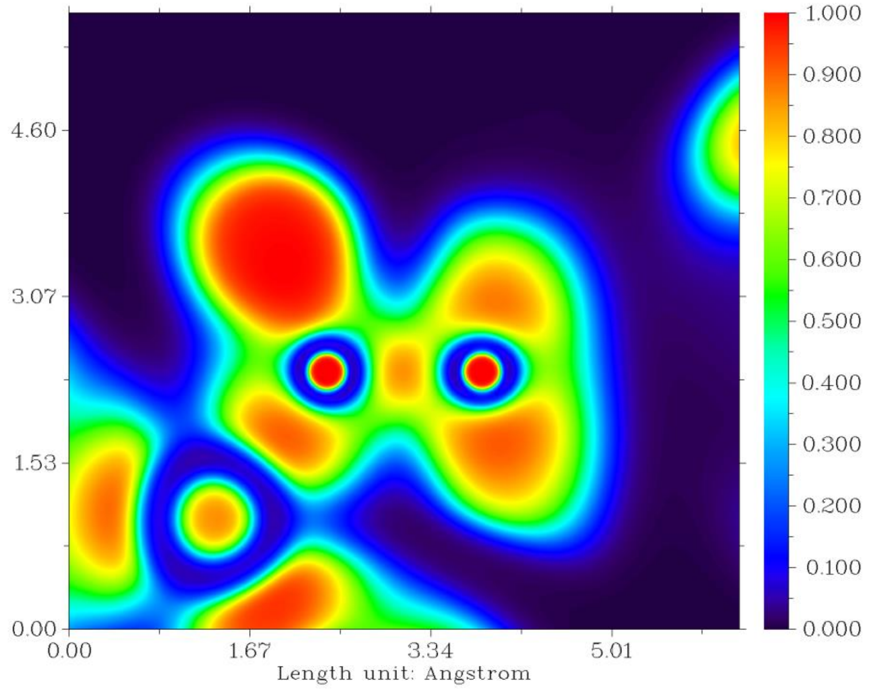

Figure S 32: 2D ELF diagram of **1** plotted in the N2-N1-H42 plane.

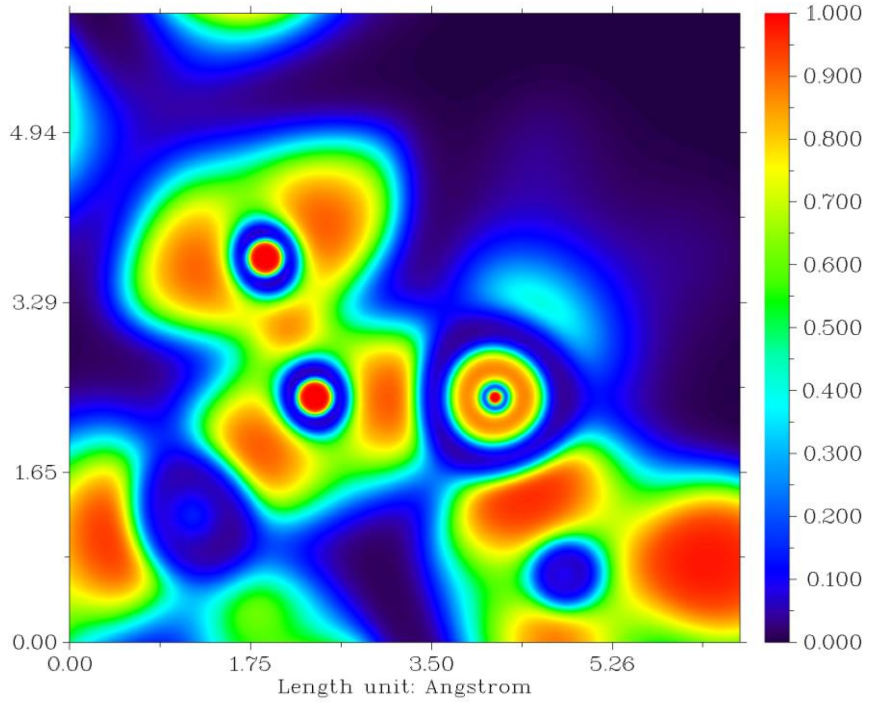

Figure S 33: 2D ELF diagram of **1** plotted in the Si1-N2-N1 plane.

9.3.3.2 ELF analysis of $1^{\cdot+}$

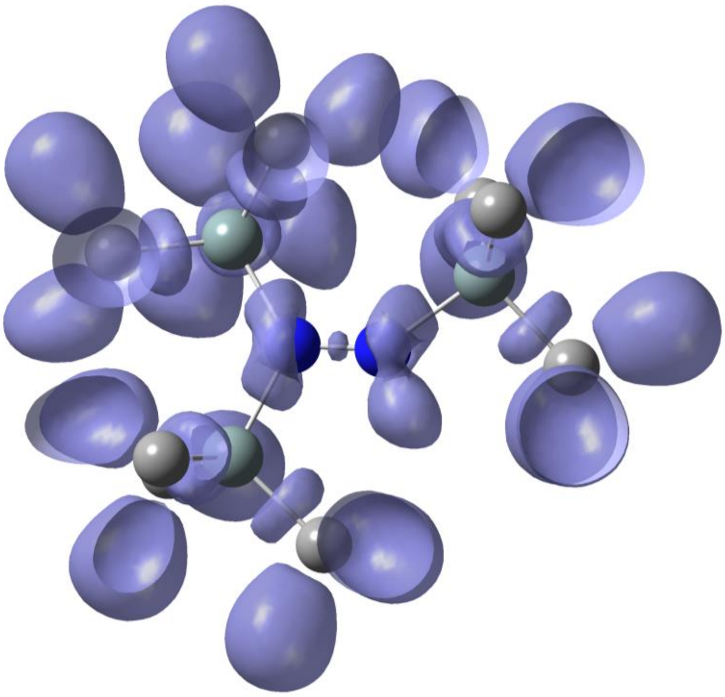

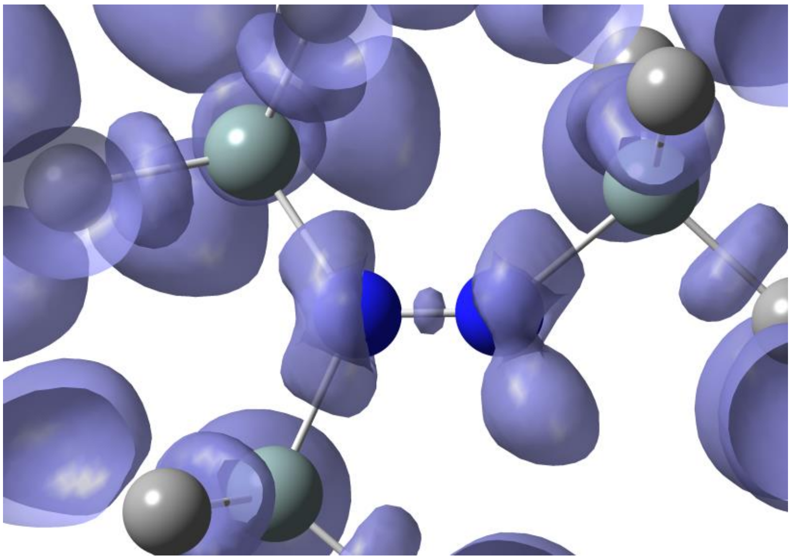

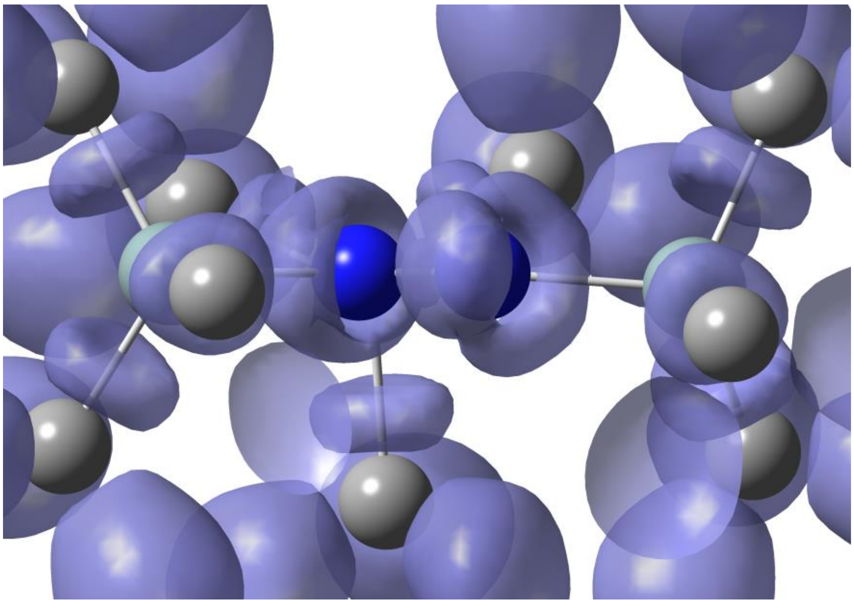

Figure S 34: 3D-ELF plot of **1**$^{\cdot+}$ (whole cation top, zoomed view's to the NN unit middle and bottom) hydrogen atoms were omitted for clarity, and z-clip function was used for a better understanding.

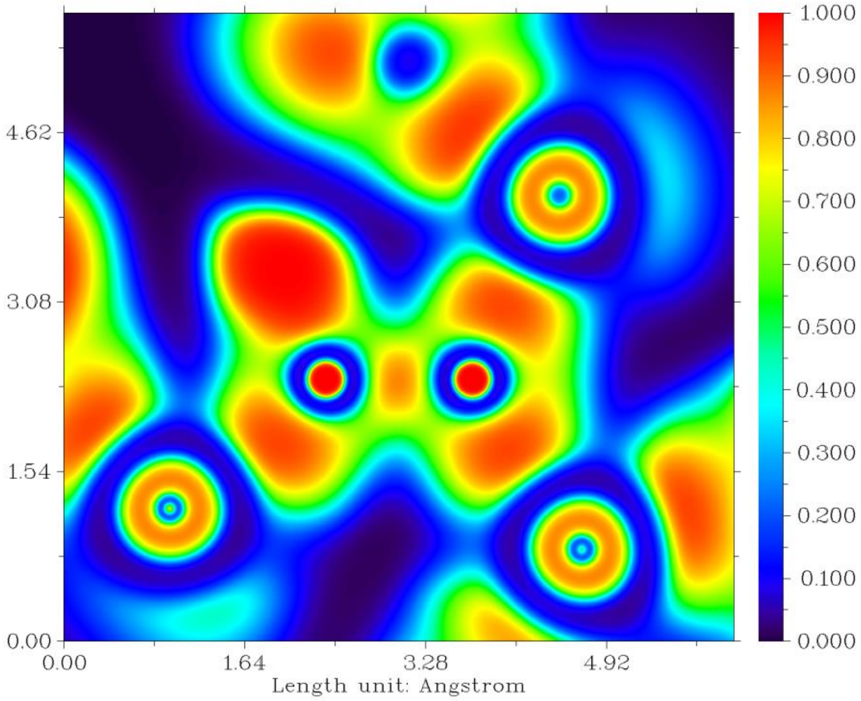

Figure S 35: 2D ELF diagram of **1**$^{\cdot+}$ plotted in the N2-N1-H42 plane.

9.3.3.3 ELF analysis of 2^+

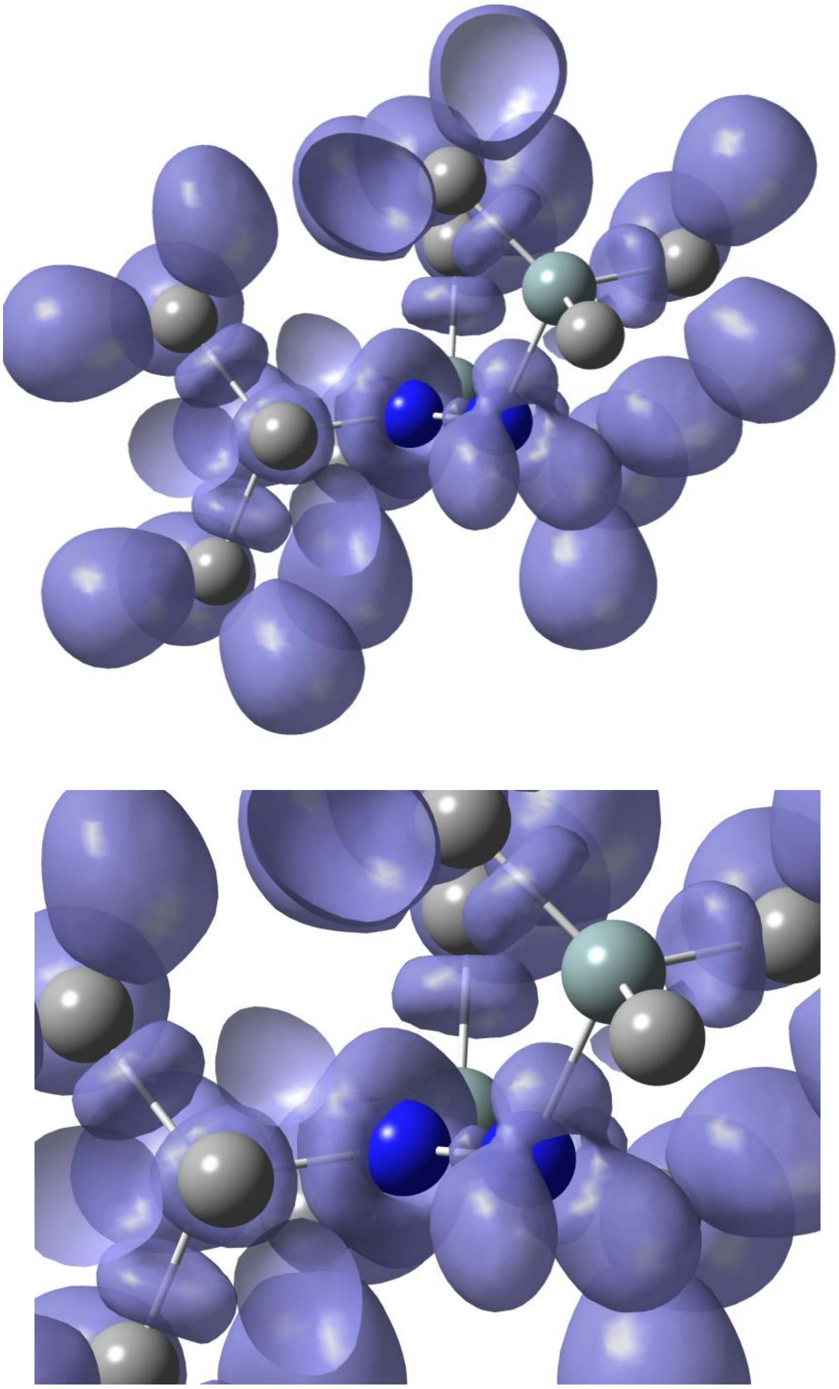

Figure S 36: 3D-ELF plot of **2**$^+$ (whole cation top, zoomed view to the NN unit bottom) hydrogen atoms were omitted for clarity, and z-clip function was used for a better understanding.

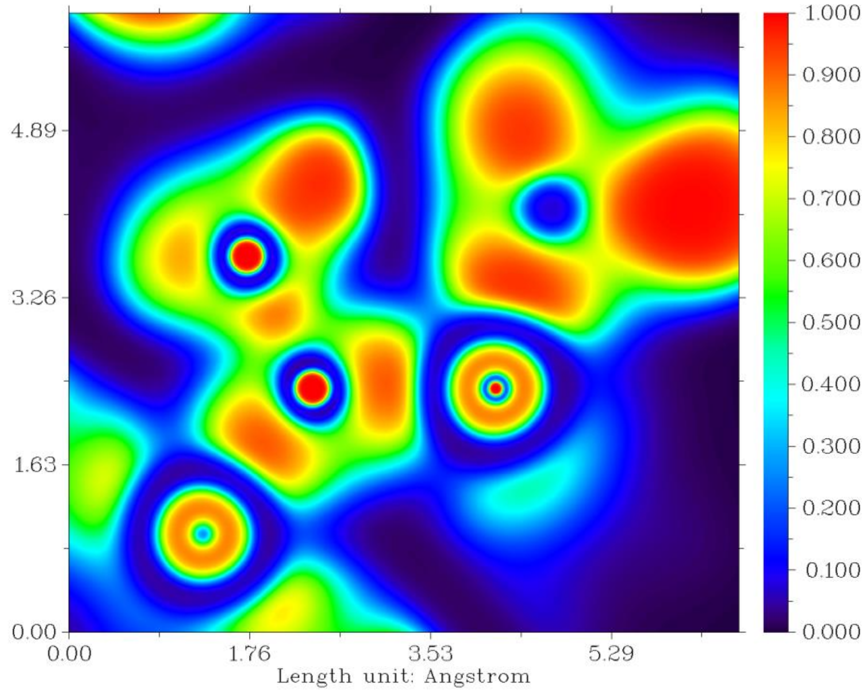

Figure S 37: 2D ELF diagram of **2**$^{+}$ plotted in the Si6-N4-N3 plane.

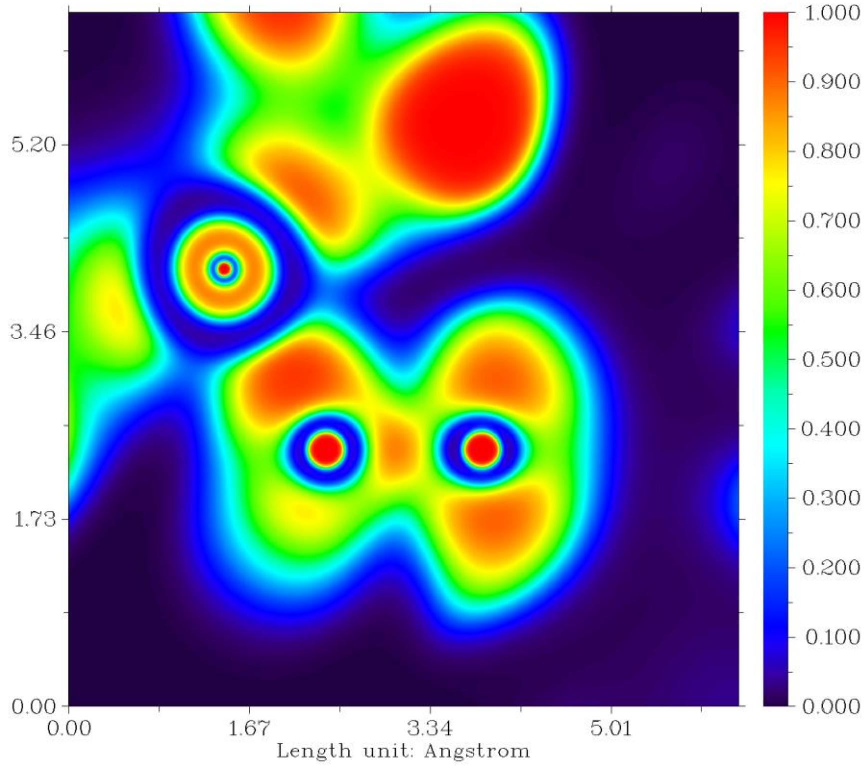

Figure S 38: 2D ELF diagram of **2**$^{+}$ plotted in the N4-N3-Si5 plane.

9.3.3.4 ELF analysis of 3^+

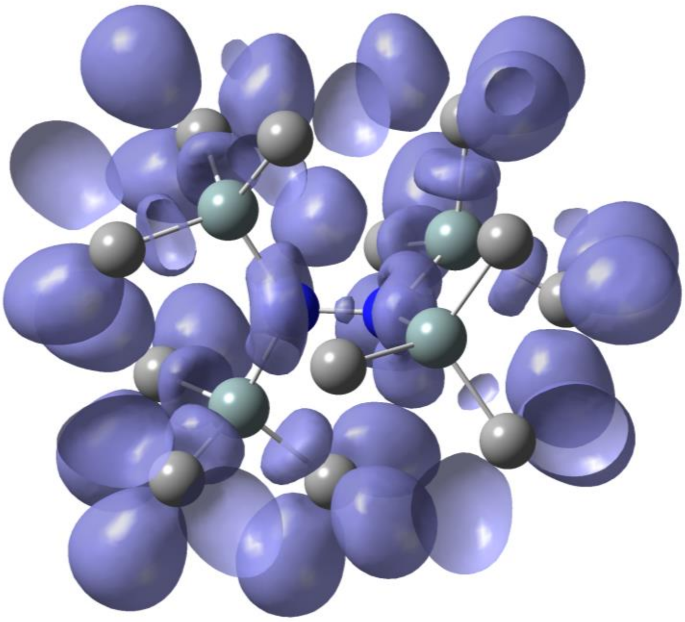

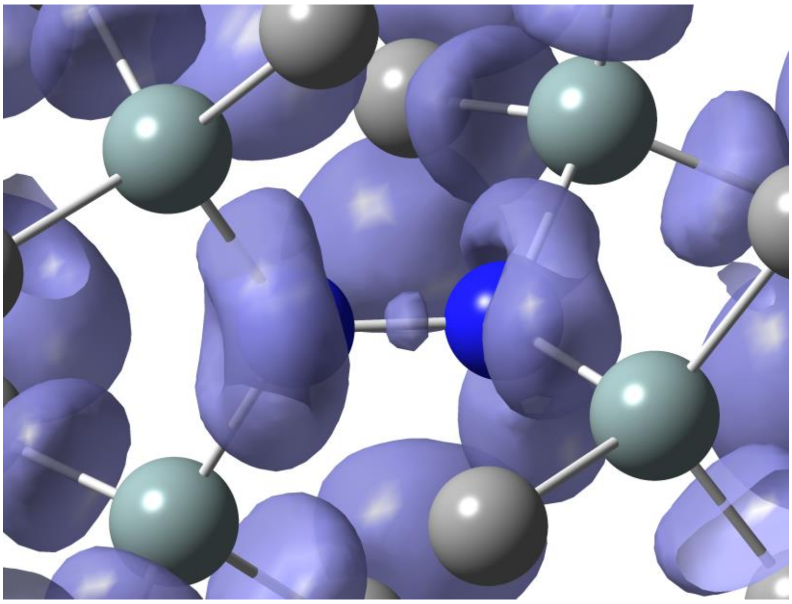

Figure S 39: 3D-ELF plot of $\mathbf{3^+}$ (whole cation top, zoomed view to the NN unit bottom) hydrogen atoms were omitted for clarity, and z-clip function was used for a better understanding.

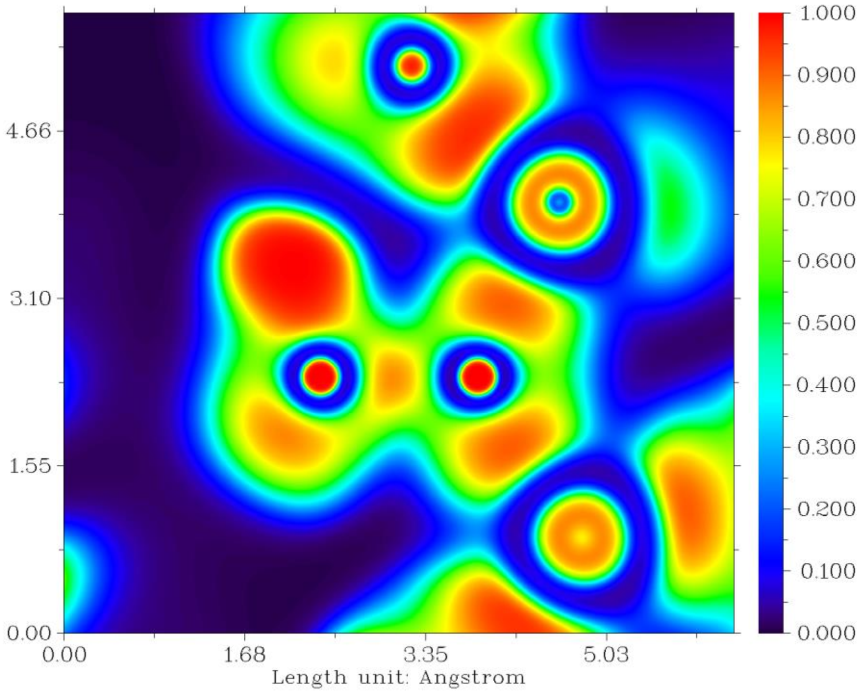

Figure S 40: 2D ELF diagram of **3**$^+$ plotted in the N2-N1-H55 plane.

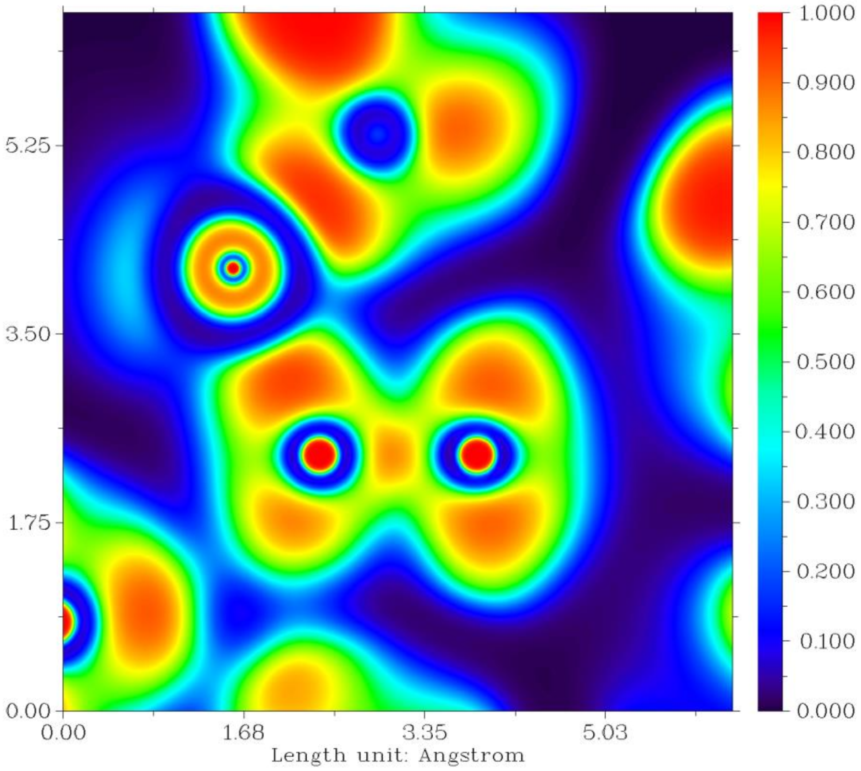

Figure S 41: 2D ELF diagram of **3**$^+$ plotted in the N2-N1-Si4 plane.

9.3.4 Visualisation of Molecular Orbitals (MO’s)

9.3.4.1 Selected MO’s of 1

Table S 39: Visualization of selected molecular orbitals of **1**, focusing on orbitals involving the NN unit.

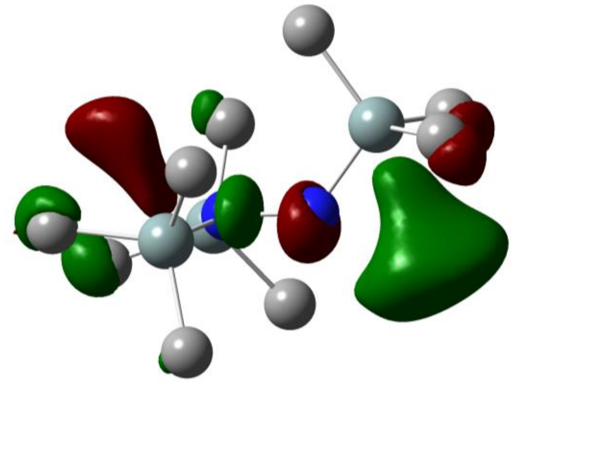	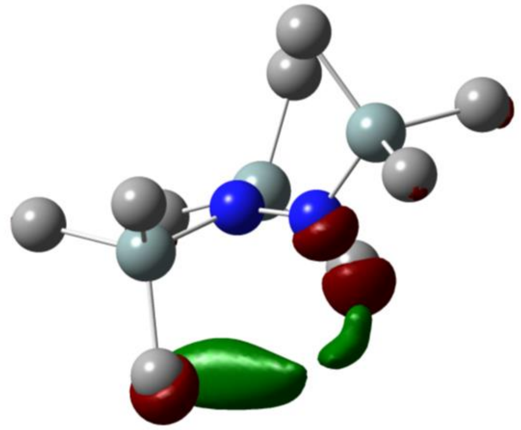
LUMO +1 (+0.819 eV) iso = 0.04	LUMO (+0.354 eV) iso = 0.04
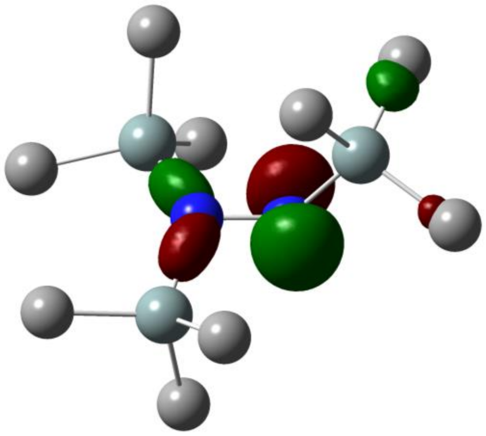	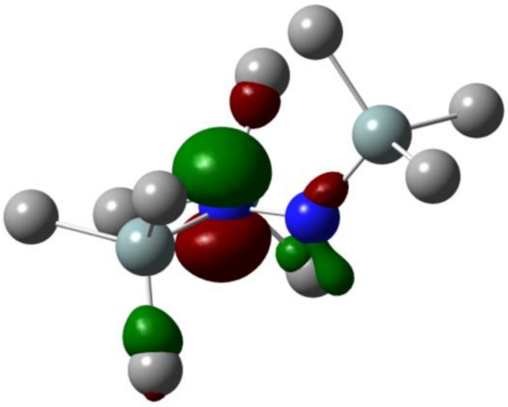
HOMO (-6.429 eV) iso = 0.08	HOMO-1 (-6.657 eV) iso = 0.08

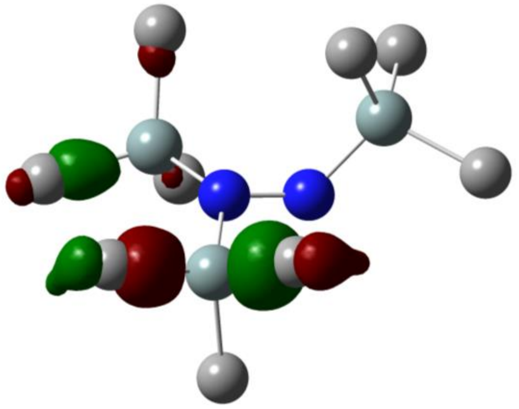	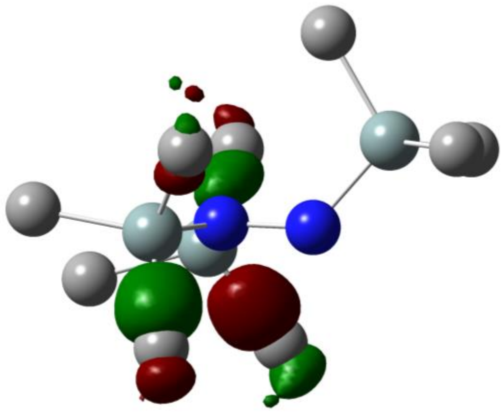
HOMO-2 (-8.235 eV) iso = 0.08	HOMO-3 (-8.348 eV) iso = 0.08
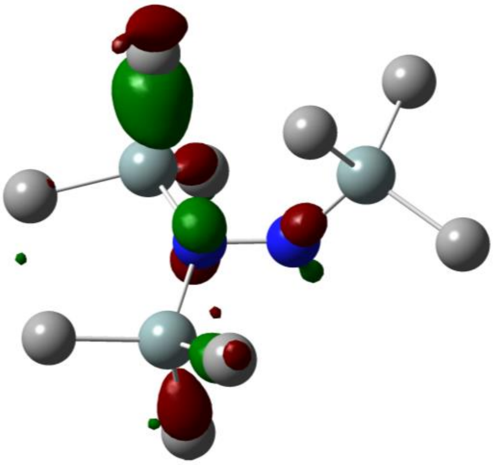	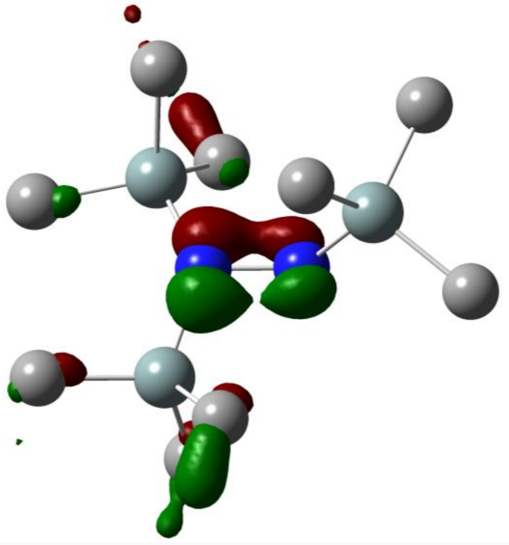
HOMO-7 (-8.733 eV) iso = 0.08	HOMO-8 (-9.958 eV) iso = 0.08
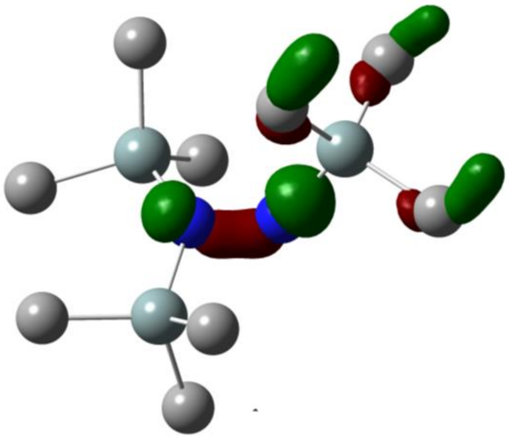	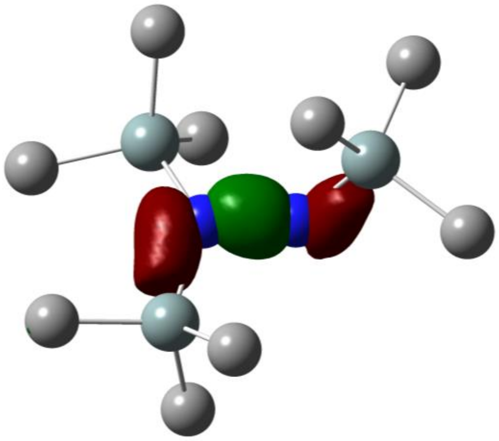
HOMO-9 (-10.211 eV) iso = 0.08	HOMO-29 (-13.462 eV) iso = 0.08

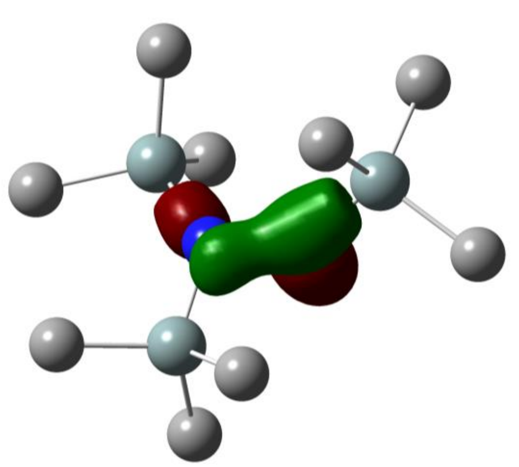	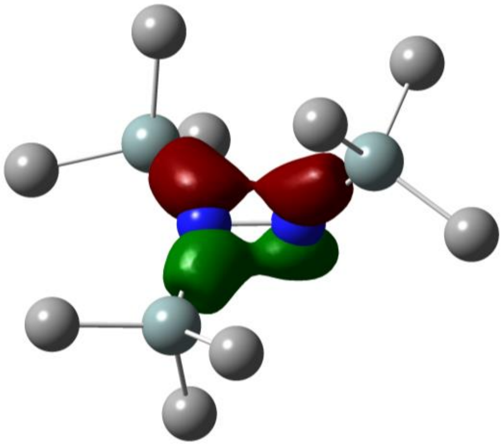
HOMO-30 (-13.907 eV) iso = 0.08	HOMO-31 (-14.006 eV) iso = 0.08
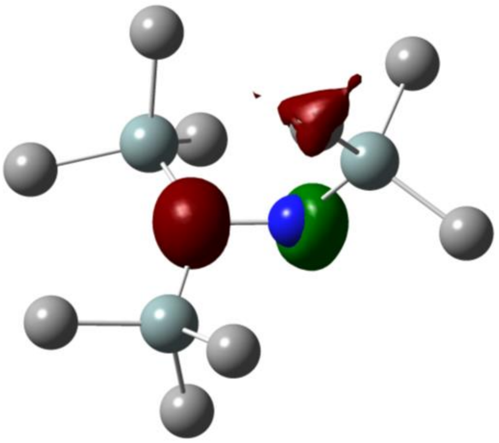	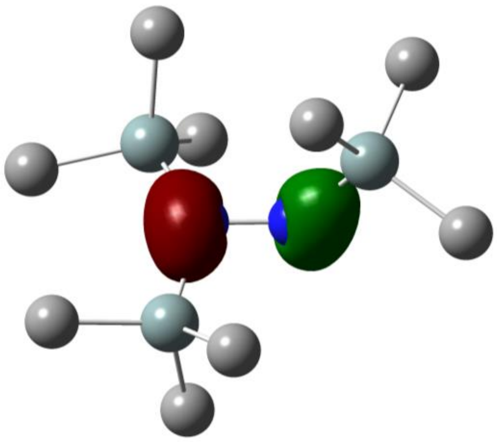
HOMO-38 (-19.044 eV) iso = 0.08	HOMO-41 (-20.459 eV) iso = 0.08
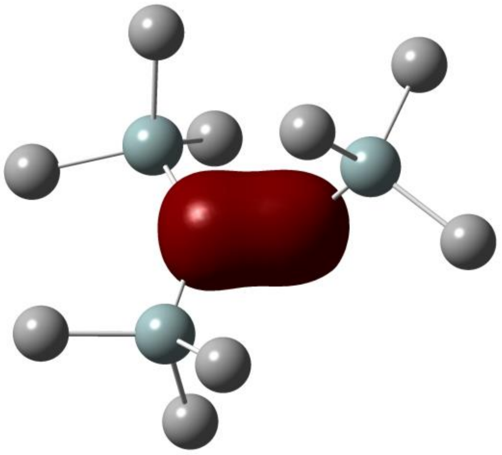	
HOMO-42 (-25.655eV) iso = 0.08	

9.3.4.2 Selected MO's of $1^{\cdot+}$

Table S 40: Visualization of selected α-molecular orbitals of $\mathbf{1}^{\cdot+}$, focusing on orbitals involving the NN unit.

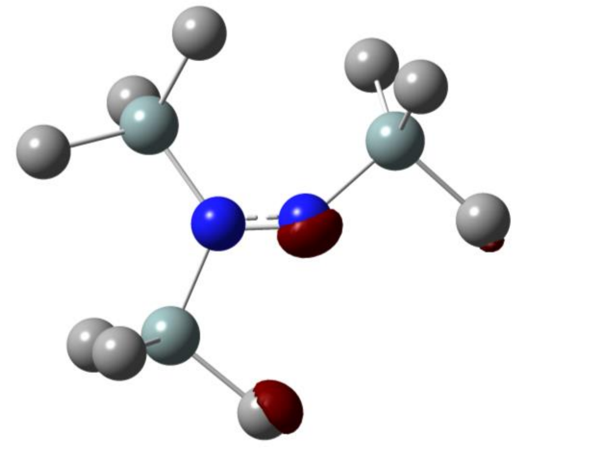	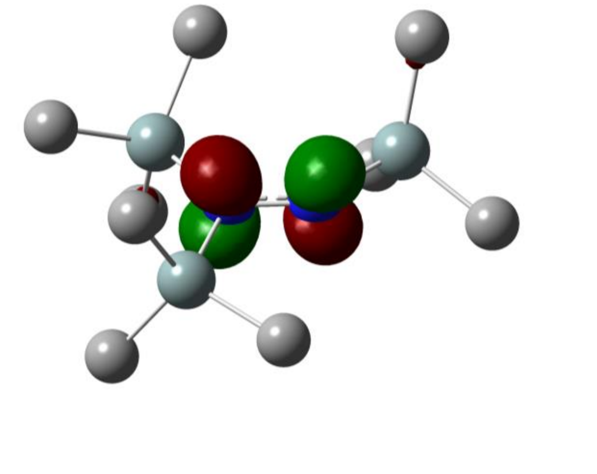
LUMO (-3.014 eV) iso = 0.06	SOMO (-10.107 eV) iso = 0.08
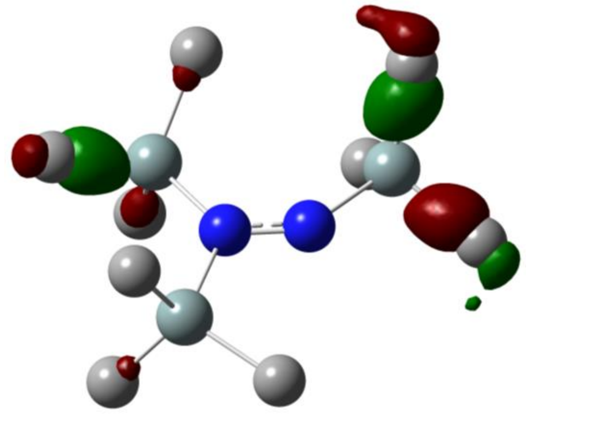	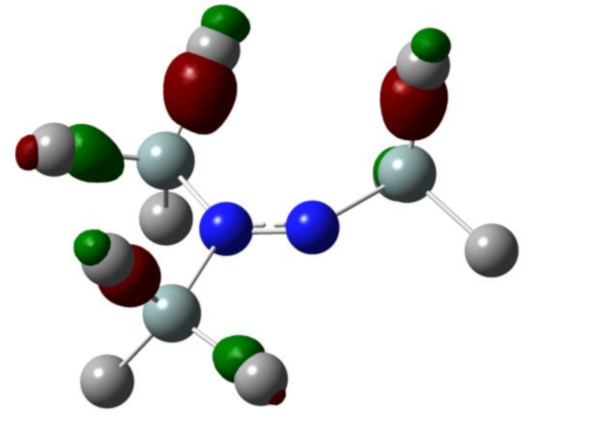
HOMO-1 (-12.188 eV) iso = 0.08	HOMO-2 (-12.205 eV) iso = 0.08
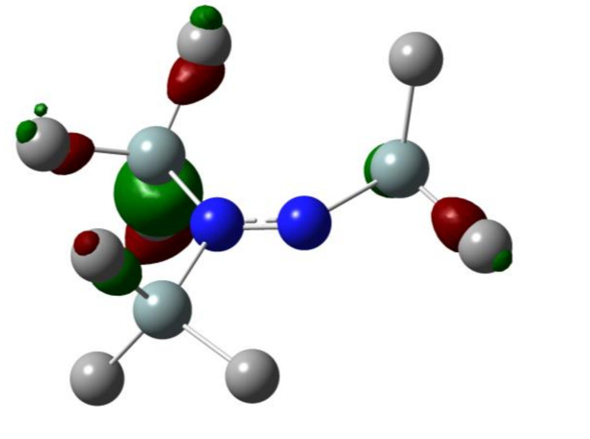	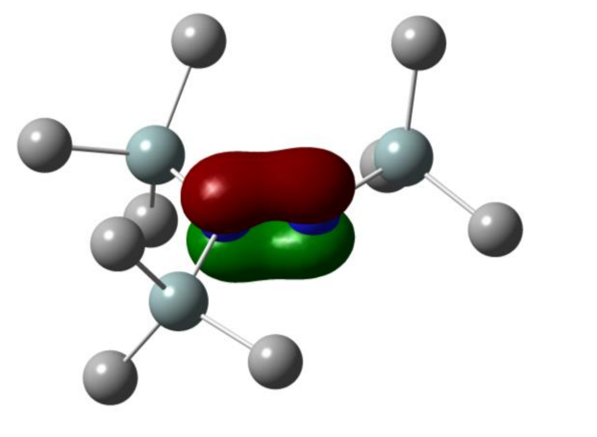
HOMO-3 (-12.279 eV) iso = 0.08	HOMO-28 (-16.524 eV) iso = 0.08

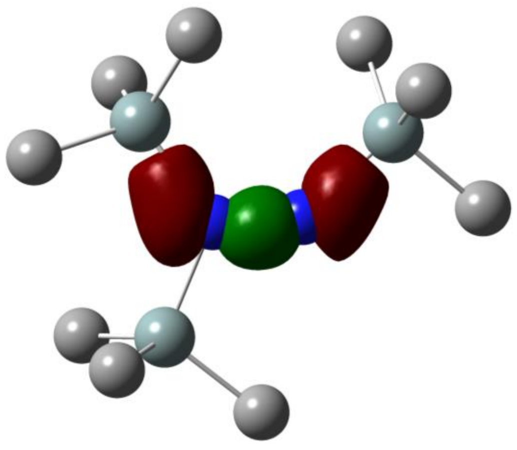	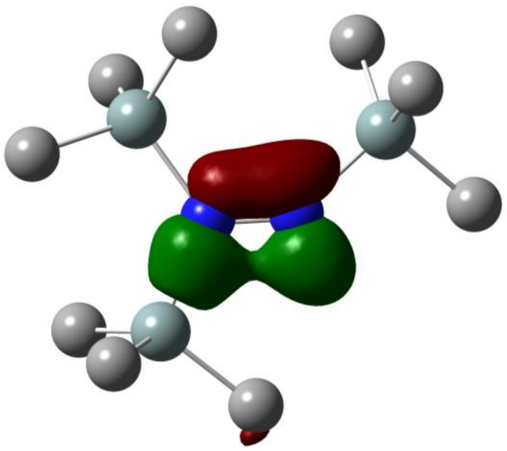
HOMO-30 (-19.038 eV) iso = 0.08	HOMO-31 (-19.477 eV) iso = 0.08
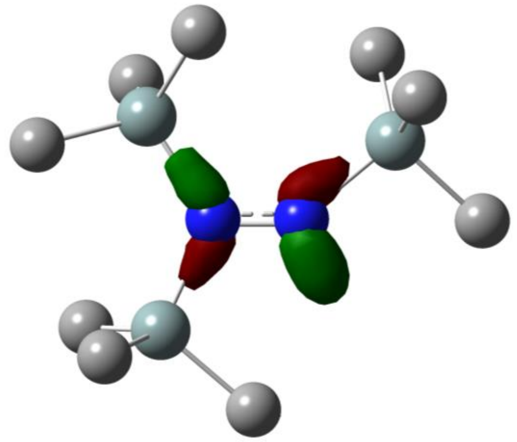	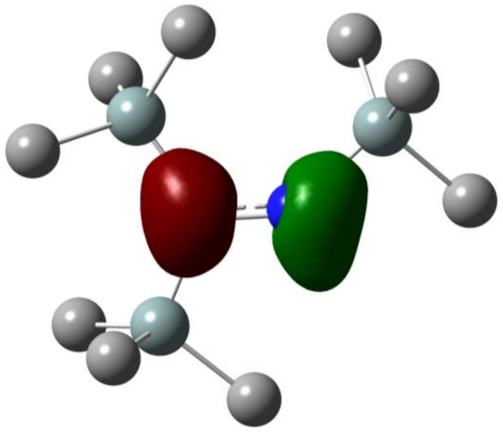
HOMO-40 (-16.912 eV) iso =0.08	HOMO-41 (-25.236 eV) iso = 0.08
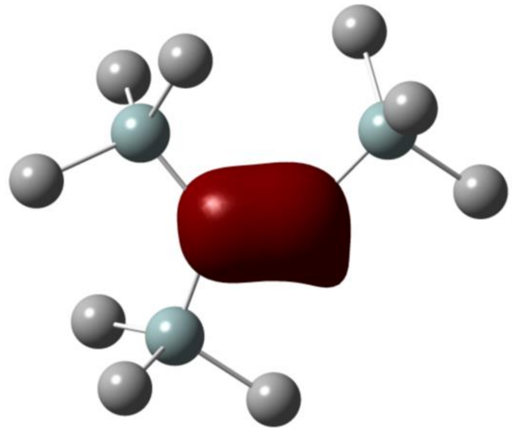	
HOMO-42 (-32.075 eV) iso = 0.08	

Table S 41: Visualization of spin SCF density of **1**$^{\cdot+}$.

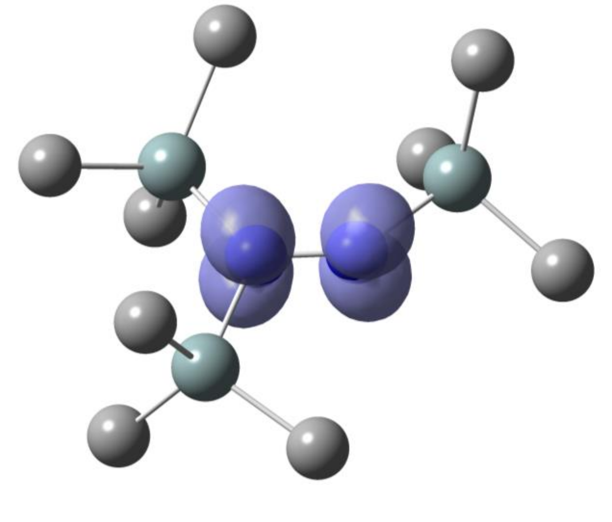

Spin density (iso = 0.008)

Table S 42: Selected Mulliken spin densities and charges compared to Natural spin densities and charges taken from NBO analysis of **1**$^{\cdot+}$.

Atom	Mulliken spin density	Natural spin density	Mulliken charge	Natural charge
N1	0.434	0.461	-0.160	-0.605
N2	0.452	0.492	-0.058	-0.743
Si3	0.023	-0.005	0.377	1.779
Si4	0.019	-0.007	0.279	1.805
Si5	0.019	-0.010	0.302	1.804

10 References

[1] W. L. F. Armarego, C. L. L. Chai, **2013**, *7*, 141; Purification of Laboratory Chemicals. Oxford: Butterworth-Heinemann.

[2] a) G. B. Dunks, K. Palmer-Ordonez, *Inorg. Chem.* **1978**, *17*, 1514–1516; b) G. B. Dunks, K. Barker, E. Hedaya, C. Hefner, K. Palmer-Ordonez, P. Remec, *Inorg. Chem.* **1981**, *20*, 1692–1697; c) A. Franken, B. T. King, J. Rudolph, P. Rao, B. C. Noll, J. Michl, Collect. Czech. *Chem. Commun.* **2001**, *66*, 1238–1249; d) Z. Xie, T. Jelinek, R. Bau, C. A. Ree, *J. Am. Chem. Soc.* **1994**, *116*, 1907–1913.

[3] G. M. Whitesides, F. D. Gutowski, *J. Org. Chem.* **1976**, *41*, 2882–2825.

[4] I. Krossing, *Chem. Eur. J.* **2001**, *7*, 490–502.

[5] M. Farooq Ibad, A. Schulz, A. Villinger *Organometallics* **2019**, *38*, 1445–1458.

[6] H. Schmidbaur, W. Bublak, B. Huber, G. Reber, G. Miiller, *Angew. Chem.* **1986**, *98*, 1108–1109.

[7] Sheldrick, G. M.: SHELXS-97: Program for the Solution of Crystal Structures, University of Göttingen, Germany **1997**.

[8] Sheldrick, G. M.: SHELXL-97: Program for the Refinement of Crystal Structures, University of Göttingen, Germany **1997**.

[9] Sheldrick, G. M.: SADABS. Version 2. University of Göttingen, Germany **2004**.

[10] W. Baumann, D. Michalik, F. Reiß, A. Schulz and A. Villinger, *Angew. Chem. Int. Ed.*, 2014, **53**, 3250-3253.

[11] T. Spałek, P. Pietrzyk, Z. Sojka, *J. Chem. Inf. Model.* **2005**, *45*, 18–29.

[12] Gaussian 09, Revision C.01, M. J. Frisch, G. W. Trucks, H. B. Schlegel, G. E. Scuseria, M. A. Robb, J. R. Cheeseman, G. Scalmani, V. Barone, B. Mennucci, G. A. Petersson, H. Nakatsuji, M. Caricato, X. Li, H. P. Hratchian, A. F. Izmaylov, J. Bloino, G. Zheng, J. L. Sonnenberg, M. Hada, M. Ehara, K. Toyota, R. Fukuda, J. Hasegawa, M. Ishida, T. Nakajima, Y. Honda, O. Kitao, H. Nakai, T. Vreven, J. A. Montgomery, Jr., J. E. Peralta, F. Ogliaro, M. Bearpark, J. J. Heyd, E. Brothers, K. N. Kudin, V. N. Staroverov, T. Keith, R. Kobayashi, J. Normand, K. Raghavachari, A. Rendell, J. C. Burant, S. S. Iyengar, J. Tomasi, M. Cossi, N. Rega, J. M. Millam, M. Klene, J. E. Knox, J. B. Cross, V. Bakken, C. Adamo, J. Jaramillo, R. Gomperts, R. E. Stratmann, O. Yazyev, A. J. Austin, R. Cammi, C. Pomelli, J. W. Ochterski, R. L. Martin, K. Morokuma, V. G. Zakrzewski, G. A. Voth, P. Salvador, J. J. Dannenberg, S. Dapprich, A. D. Daniels, O. Farkas, J. B. Foresman, J. V. Ortiz, J. Cioslowski, D. J. Fox, Gaussian, Inc., Wallingford CT, 2010.

[13] Y. Zhao, D. G. Truhlar, *Theor. Chem. Acc.* **2008**, 120, 215–41.

[14] (a) Y. Zhao, D. G. Truhlar, *Theor. Chem. Acc.* **2007**, *120*, 215–241; (b) T. H. Dunning, Jr. J. Chem. Phys. 1989, 90, 1007; (c) D.E. Woon and T.H. Dunning, Jr. *J. Chem. Phys.* **1993**, *98*, 1358; (d) K.A. Peterson and T.H. Dunning, Jr. *J. Chem. Phys.* **2002**, *117*, 10548.

[15] Weigend, F.; Ahlrichs, R. *Phys. Chem. Chem. Phys.* **2005**, *7*, 3297–3305.

[16] a) Grimme, S.; Antony, J.; Ehrlich, S.; Krieg, H., *J. Chem. Phys.* **2010**, *132*, 154104; b) Grimme, S.; Ehrlich, S.; Goerigk, L., *J. Comput. Chem.* **2011**, *32*, 1456–1465.

[17] For a more thorough discussion on the basis set dependence of molecular geometries, see Klapötke, T. M.; Schulz, A. Ab initio Methods in Main Group Chemistry with an invited Chapter by R. D. Harcourt about VB Theory, John Wiley & Sons, New York, **1998**.

[18] Lu, T.; Chen, F., *J. Comput. Chem.* **2012**, *33*, 580.

[19] GaussView, Version 6.1, Roy Dennington, Todd A. Keith, and John M. Millam, Semichem Inc., Shawnee Mission, KS, **2016**.

[20] Mercury: http://www.ccdc.cam.ac.uk/mercury/
